# Local changes in potassium ions regulate input integration in active dendrites

**DOI:** 10.1371/journal.pbio.3002935

**Published:** 2024-12-04

**Authors:** Malthe S. Nordentoft, Naoya Takahashi, Mathias S. Heltberg, Mogens H. Jensen, Rune N. Rasmussen, Athanasia Papoutsi

**Affiliations:** 1 Niels Bohr Institute, University of Copenhagen, Copenhagen, Denmark; 2 University of Bordeaux, CNRS, Interdisciplinary Institute for Neuroscience (IINS), UMR 5297, Bordeaux, France; 3 Center for Translational Neuromedicine, University of Copenhagen, Copenhagen, Denmark; 4 Institute of Molecular Biology and Biotechnology, Foundation for Research and Technology—Hellas, Crete, Greece; Institute of Science and Technology Austria, AUSTRIA

## Abstract

During neuronal activity, the extracellular concentration of potassium ions ([K^+^]_o_) increases substantially above resting levels, yet it remains unclear what role these [K^+^]_o_ changes play in the dendritic integration of synaptic inputs. We here used mathematical formulations and biophysical modeling to explore the role of synaptic activity-dependent K^+^ changes in dendritic segments of a visual cortex pyramidal neuron, receiving inputs tuned to stimulus orientation. We found that the spatial arrangement of inputs dictates the magnitude of [K^+^]_o_ changes in the dendrites: Dendritic segments receiving similarly tuned inputs can attain substantially higher [K^+^]_o_ increases than segments receiving diversely tuned inputs. These [K^+^]_o_ elevations in turn increase dendritic excitability, leading to more robust and prolonged dendritic spikes. Ultimately, these local effects amplify the gain of neuronal input–output transformations, causing higher orientation-tuned somatic firing rates without compromising orientation selectivity. Our results suggest that local, activity-dependent [K^+^]_o_ changes in dendrites may act as a “volume knob” that determines the impact of synaptic inputs on feature-tuned neuronal firing.

## Abstract 2

This study uses a computational modeling approach investigate how local changes in potassium concentration regulate dendritic integration of synaptic inputs, revealing that ’potassium hotspots’ act as a gain control mechanism.

## Introduction

Throughout the nervous system, neuronal activity and ionic changes in the extracellular environment are bidirectionally linked. Yet, extracellular ion changes are not traditionally considered an integral part of neuronal signaling and information processing. Amidst the activity-dependent fluctuations of extracellular ionic concentrations, K^+^ ions emerge as particularly intriguing due to their pivotal role in shaping neuronal excitability and membrane potential (V_m_). At rest, the extracellular concentration of K^+^ ([K^+^]_o_) in the brain is normally between 2.7 and 3.5 mM [1–3]. It has been experimentally shown that during sensory stimulation, motor network activity, sleep oscillations, or behavioral state transitions, [K^+^]_o_ increases by 0.25–2 mM [4–14], while it can rise up to 7–12 mM during hypersynchronous neuronal activity [15–17]. The [K^+^]_o_ increase weakens the outward K^+^ driving force, resulting in a less negative K^+^ reversal potential (*E*_K_^+^), which powerfully affects the V_m_, excitability, and firing patterns of neurons [11,14,18–25]. A major source contributing to the [K^+^]_o_ changes at the synaptic level is K^+^ efflux from excitatory glutamatergic receptors, and in particular from NMDA receptors, when compared to other calcium- or voltage-dependent potassium channels [16,21,26]. Importantly, previous work has shown that such postsynaptic NMDA receptor-mediated K^+^ efflux is highly localized, and can signal to presynaptic axons [21,27], pointing to local [K^+^]_o_ changes acting as a modulator of presynaptic transmission. Despite the experimental evidence that synaptic changes can be highly localized, we still lack the experimental tools to systematically disentangle the effect of activity-dependent [K^+^]_o_ changes at such a fine-scale level. In addition, whether the activity-mediated [K^+^]_o_ changes can modulate postsynaptic integration of synaptic inputs remains elusive.

Pyramidal neurons possess elaborate dendritic trees that contain a variety of voltage-dependent ion channels such as Na^+^, K^+^, and Ca^2+^ channels. These channels allow for complex, active responses to synaptic inputs, including the generation of local action potentials, called dendritic spikes [28–31]. The spatial arrangement of synaptic inputs is an important factor for determining dendritic spike initiation [32–38]. These dendritic spikes support the nonlinear summation of synaptic inputs, which in turn alter the neurons’ input–output function and can enhance sensory feature selectivity in vivo [39–42]. Interestingly, artificial, pharmacological manipulation of dendritic K^+^ currents affects dendritic excitability and dendritic spikes, indicating that K^+^ currents can act as a regulator of dendritic integration [43,44]. Based on the above findings, we here hypothesize that physiological, synaptic activity-mediated changes in [K^+^]_o_ can locally regulate the active dendritic properties and thus shape the nonlinear integration of inputs and sensory processing.

To test the hypothesis, we used proof-of-concept mathematical formulations and biophysical modeling to investigate the effect of the reported physiological [K^+^]_o_ changes. We focus on the organization of excitatory synaptic inputs tuned to the stimulus orientation on dendritic branches of a pyramidal neuron in the visual cortex [42], as we have previously experimentally shown that visual cortical responses are dynamically regulated by [K^+^]_o_ and brain state [14]. Using statistical analysis, as well as abstract and morphologically detailed biophysical models of dendrites and neurons, we show that the arrangement of tuned inputs determines the magnitude of activity-dependent [K^+^]_o_ changes in dendrites. Specifically, dendritic segments with similarly tuned synaptic inputs can attain substantially higher [K^+^]_o_ elevations than segments with diversely tuned inputs. These [K^+^]_o_ elevations in turn depolarize the *E*_K_^+^ which increases the reliability of dendritic spikes and prolongs their duration, but without compromising their stimulus selectivity. Ultimately, these local effects amplify the gain of neuronal input–output transformations, leading to higher firing rates at the soma without affecting feature selectivity. Overall, our results suggest a prominent role for local, activity-dependent, dendritic “[K^+^]_o_ hotspots” [21,27] in shaping dendritic integration of synaptic inputs. Importantly, using the visual cortex as a framework of study and grounded by experimental data, we provide a proof-of-concept that grouping of co-tuned inputs creates dendritic [K^+^]_o_ hotspots that regulate dendritic integration, a property can be generalized to other sensory features, brain regions, or animal species, with the requirement that synaptic inputs are clustered on a spatial and temporal scale [32,42,45–50].

## Results

### Extracellular space size, intracellular potassium concentration changes, and stimulus orientation shape *E*_K_^+^ shifts

**Fig 1:**
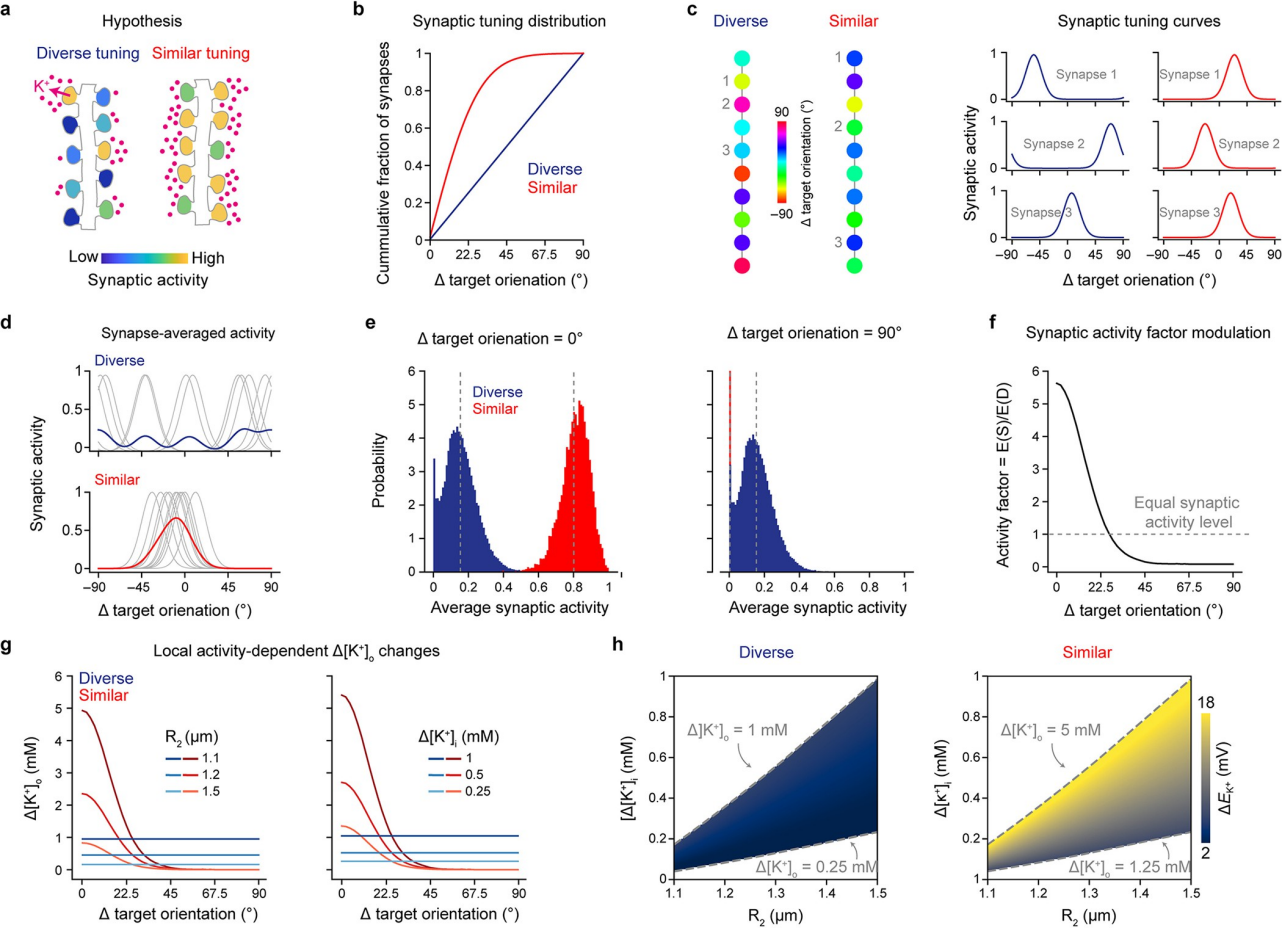
Dendritic segments with similarly tuned inputs support higher [K^+^]_o_ changes and *E*_K_^+^ shifts. (a) Diagram of the proposed hypothesis that dendritic segments with similarly tuned inputs can attain higher [K^+^]_o_ increases than segments with diversely tuned inputs. (b) Cumulative fraction of synapse orientation preferences relative to target orientation for dendritic segments with diverse or similar orientation tuning. Reproducing results of [42]. (c) Example dendritic segments with diverse and similar input tuning regimes (left). Synaptic tuning curves for different orientation-tuned stimuli, as per [55], normalized by the maximum activity (right). Note that the x-axis corresponds to stimulus orientation relative to target orientation. The resulting activity shows the tuning curves for the synapses indicated on the corresponding dendritic segment on the left. Based on the distributions of panel b and the synaptic tuning curves, we acquired a statistical approximation of the expected *E*_K_^+^ shifts without explicitly modeling membrane dynamics. (d) Example synapse-averaged tuning curves for dendritic segments from the diverse (top) and similar (bottom) tuning regimes. Tuning curves of individual synapses are in gray and average is in color. (e) Probability distributions of average synaptic activity for the diverse and similar tuning regimes for the target (left) and orthogonal to the target (right) orientations. Dotted lines indicate expectation value. (f) Synaptic activity factor, defined as the ratio between expected synaptic activity for similarly—**E** (S) and diversely—**E** (D) tuned segments as a function of stimulus orientation relative to target orientation. (g) Δ[K^+^]_o_ as a function of stimulus orientation relative to target orientation for the diverse and similar tuning regimes. Left: different extracellular space radii (R_2_) with constant [K^+^]_i_ reductions (Δ[K^+^]_i_ = 1 mM). Right: different Δ[K^+^]_i_ with constant R_2_ ($\sqrt{2\mu m}$). (h) Heat maps showing the range of Δ*E*_K_^+^ for the diverse (left) and similar (right) tuning regimes as a function of Δ[K^+^]_i_ and R_2_ at the target orientation. Dotted lines indicate the corresponding Δ[K^+^]_o_ range.

To identify the expected *E*_K_^+^ shifts under different conditions, we first undertook an analytical approach that did not include simulation of the membrane potential dynamics. We hypothesized that dendritic segments with similarly tuned inputs can attain higher [K^+^]_o_ changes and *E*_K_^+^ shifts than segments with diversely tuned inputs and that this could, in turn, affect dendritic excitability (**Fig 1a**). To investigate this, we sampled the synaptic activity of a hypothetical 10 μm segment, populated with 7 to 13 synapses, yielding a synaptic density comparable to the neocortex [51–53]. We chose this length scale because functional clusters, comprised of inputs with similar tuning preferences, typically are formed within 5 to 10 μm [32,45–47]. To model feature-tuned synaptic inputs, we used visual orientation tuning as our framework. Previous work identified dendritic segments receiving inputs with similar or diverse orientation tuning [42,54]. To capture this, we sampled synaptic orientation preferences from a circular normal distribution for the similarly tuned regime and from a uniform distribution for the diversely tuned regime [42], and assigned tuning curves to individual synapses (**Fig 1a and 1c**). From the sampled segments, we obtained synapse-averaged tuning curves and activity distributions (**Fig 1d and 1e**). Using these, we derived an activity factor for each orientation, signifying the ratio of expected synaptic activity in segments with similar versus diverse tuning preferences (**Fig 1f**; see Methods). This showed that for stimulus orientations close to the target orientation, here arbitrarily chosen as the somatic preferred orientation set at 0°, (Δtarget orientation: 0–20°), synaptic activity levels are 2 to 5 times higher in segments with similarly tuned inputs than in segments with diversely tuned inputs. Contrary, for orientations far from the target orientation (Δtarget orientation: 45–90°), activity ceases almost entirely in segments with similar tuning while it remains constant for segments with diverse tuning, yielding activity factors below 1.

We then asked how these synaptic activity patterns are manifested in [K^+^]_o_ and *E*_K_^+^ changes. Due to experimental limitations, there is a lack of knowledge about singular ionic flux over the multiple ionic channels that support the in vitro and in vivo documented potassium concentration changes. Thus, we adopted a mean-field approach that allowed us to relate the experimental recordings of extracellular potassium to the model while keeping to the number of assumptions at a minimum. Specifically, in our approach we assumed the following conditions to be true: (1) Local increases in [K^+^]_o_ are proportional to synaptic activity levels; (2) local increases in [K^+^]_o_ are caused by K^+^ efflux from the intracellular space; and (3) [K^+^]_o_ change is stable in space and time along the short dendritic segment. The latter is motivated by the fact that (a) K^+^ ions in the extracellular space, of the spatial and temporal scale considered here (approximately 10 μm and approximately 300 ms after synaptic activity onset, respectively), can be described as well-mixed due to the high K^+^ free diffusion rate ($D_{K_{free}^{+}}=1.96\frac{\mu m^{2}}{ms}$) [56,57]; and (b) extracellular potassium remains elevated for timescales of several hundreds of ms, as documented in in vitro experiments [58,59] (see Methods for detailed analysis). Following these assumptions, we approximated the [K^+^]_o_ changes of dendritic segments by multiplying the intracellular K^+^ concentration ([K^+^]_i_) change by the volume fraction between the intra- and extracellular space:

$$\Delta {\left[K^{+}\right]}_{o}=|\Delta [K^{+}{]}_{i}|\frac{V_{In}}{V_{Ext}}=|\Delta [K^{+}{]}_{i}|\frac{R_{1}^{2}}{R_{2}^{2}-R_{1}^{2}}$$ (1)

Here, Δ[K^+^]_o_ and Δ[K^+^]_i_ are the changes in [K^+^]_o_ and [K^+^]_i_, respectively. The dendritic segment is considered as a cylinder with radius R_1_, encapsulated by the extracellular space, also described as a cylinder with radius R_2_ (see Methods, Eq 5). By keeping the dendritic diameter constant (R_1_ = 1 μm) [60,61], we limit our parameter space to 2 parameters: Δ[K^+^]_i_ and R_2_. Although we do not have data on the local dendritic [K^+^]_i_ changes, nor do we know the exact extracellular space size in the vicinity of dendritic segments, we can use Eq 1 to estimate [K^+^]_o_ changes by varying Δ[K^+^]_i_ and R_2_ within realistic ranges [62–64]. Note that as R_2_→R_1_, Δ[K^+^]_o_ increases as $\frac{1}{R_{2}^{2}-R_{1}^{2}}$ approaching infinity, and becomes undefined for R_2_ = R_1_. As R_2_→∞, Δ[K^+^]_o_ goes towards zero asymptotically. By multiplying Δ[K^+^]_i_ for the diversely tuned input regime by the synaptic activity factor (**Fig 1f**), we also obtain relative estimates of [K^+^]_o_ changes in segments receiving similarly tuned inputs. Δ[K^+^]_o_ changes are positively correlated with the Δ[K^+^]_i_ (**Fig 1g, right**), inversely correlated with the size of the extracellular space (**Fig 1g, left**) and, for the similar input regime, higher for orientations close to the target orientation but lower for orientations far from the target (**Fig 1g**).

Focusing on activity for stimuli presented at Δtarget orientation = 0°, we next identified the Δ[K^+^]_i_ and R_2_ parameters that constrained the Δ[K^+^]_o_ for the diversely tuned inputs to a range of 0.25–1 mM (**Fig 1h, left**), to reflect the changes measured by in vivo [K^+^]_o_ measurements using microelectrodes [5–7,9,10,12–14,18,65], a technique which averages the measured concentration in space and time and likely underestimates true local [K^+^]_o_ changes [66–69]. This set of parameters when applied to the similarly tuned input regime results in Δ[K^+^]_o_ changes in the range 1.25–5 mM (**Fig 1h, right**). By converting the Δ[K^+^] into shifts in *E*_K_^+^ via the Nernst equation:

$$\Delta E_{K^{+}}=-26.7\;ln\frac{[K^{+}{]}_{i}-\Delta [K^{+}{]}_{i}}{[K^{+}{]}_{o}+\Delta [K^{+}{]}_{o}}-(-)26.7\;\ln\frac{[K^{+}{]}_{i}}{[K^{+}{]}_{o}}$$ (2)

and using this parameter set, we evaluated the range of the Δ*E*_K_^+^ shifts. For diverse inputs, the Δ*E*_K_^+^ is within the range of 1.6–5.9 mV above the resting *E*_K_^+^, and for similarly tuned input, the respective range is 6–18 mV (**Fig 1h**). Based on this analytical approach, in the following sections, we use these ranges in Δ*E*_K_^+^ as a proxy for changes in [K^+^]_o_, when we explore their impact on dendritic integration in biophysical models with different levels of abstraction. Together, these data suggest that dendritic segments receiving synaptic inputs with similar tuning preferences can attain substantially higher [K^+^]_o_ and *E*_K_^+^ changes compared with segments receiving diversely tuned inputs, yet in a stimulus-specific manner.

### Dendritic spike properties and orientation selectivity for different *E*_K_^+^ shifts

**Fig 2:**
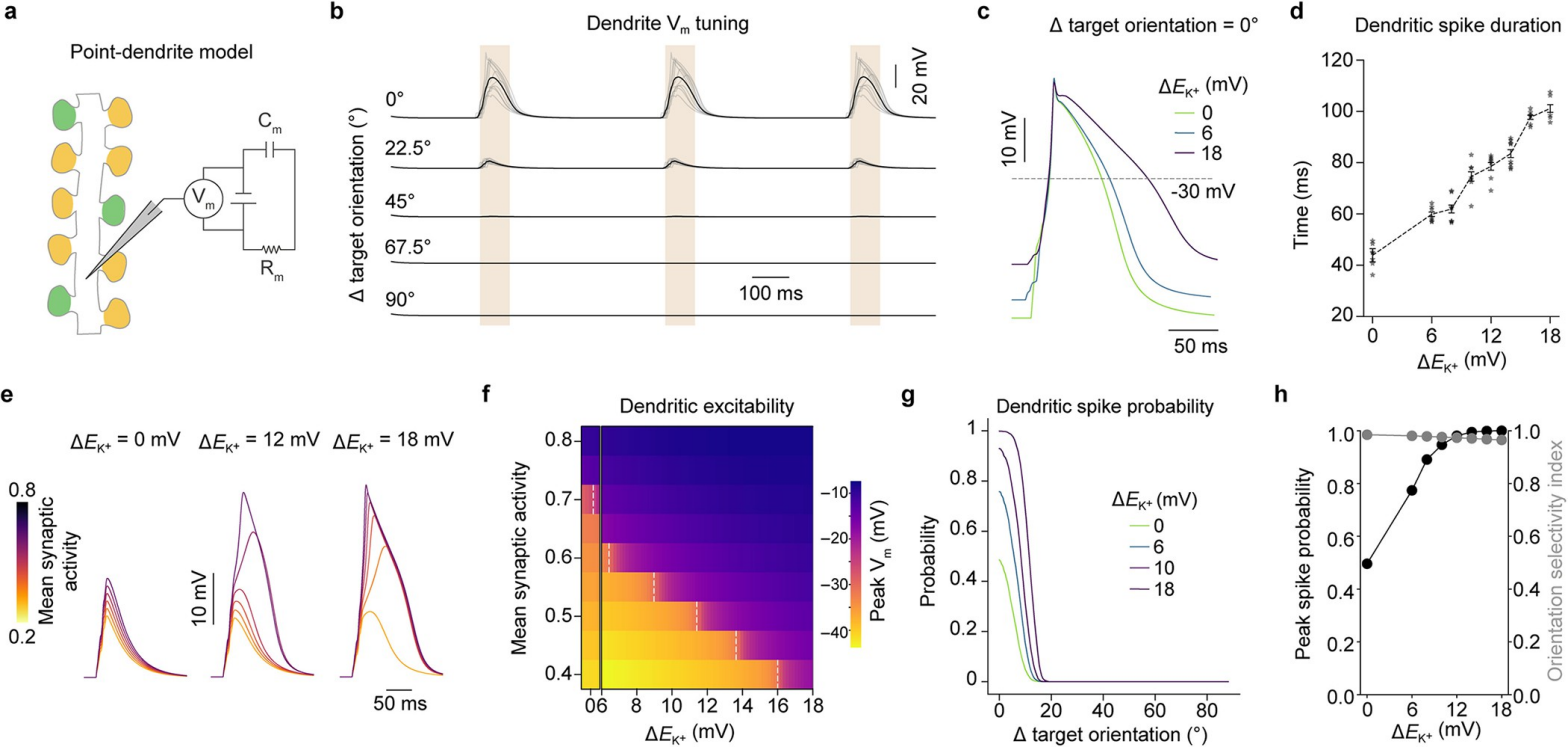
*E*_K_^+^ shifts regulate active dendritic properties. (a) Diagram of the biophysical point-dendrite model. To test the effect of local [K^+^]_o_ elevations, we imposed *E*_K_^+^ shifts within the interval of 0–18 mV. (b) Example dendrite V_m_ traces for the similar input tuning regime as a function of stimulus orientation relative to target orientation. Individual trials are in gray and average is in black. Shaded regions indicate synaptic stimulation timing. (c) Example V_m_ traces highlighting dendritic spike duration (time above –30 mV) as a function of Δ*E*_K_^+^ at the target orientation. The *E*_K_^+^ shift is induced following the first simulation event and is visible in the resting membrane potential of the dendritic segment. (d) Dendritic spike duration as a function of Δ*E*_K_^+^ at the target orientation. Error bars are mean ± SEM. (*N* = 10 simulations). (e) Example traces highlighting V_m_ response as a function of Δ*E*_K_^+^ and mean synaptic activity. (f) Heat map showing peak V_m_ depolarization as a function of Δ*E*_K_^+^ and mean synaptic activity. Dotted lines indicate the transition to dendritic spiking. Number of synapses used: *N* = 10. (g) Dendritic spike probability as a function of Δ*E*_K_^+^ and stimulus orientation relative to target orientation. Note that Δ*E*_K_^+^ values denote shifts at the target orientation and shifts for the rest of the orientations are calculated using the synaptic activity factor. (h) Dendritic spike probability at the target orientation (left axis) and orientation selectivity index (right axis) as a function of Δ*E*_K_^+^. See also **S3** and **S4 Figs** and **S1 Table**.

**Fig 3:**
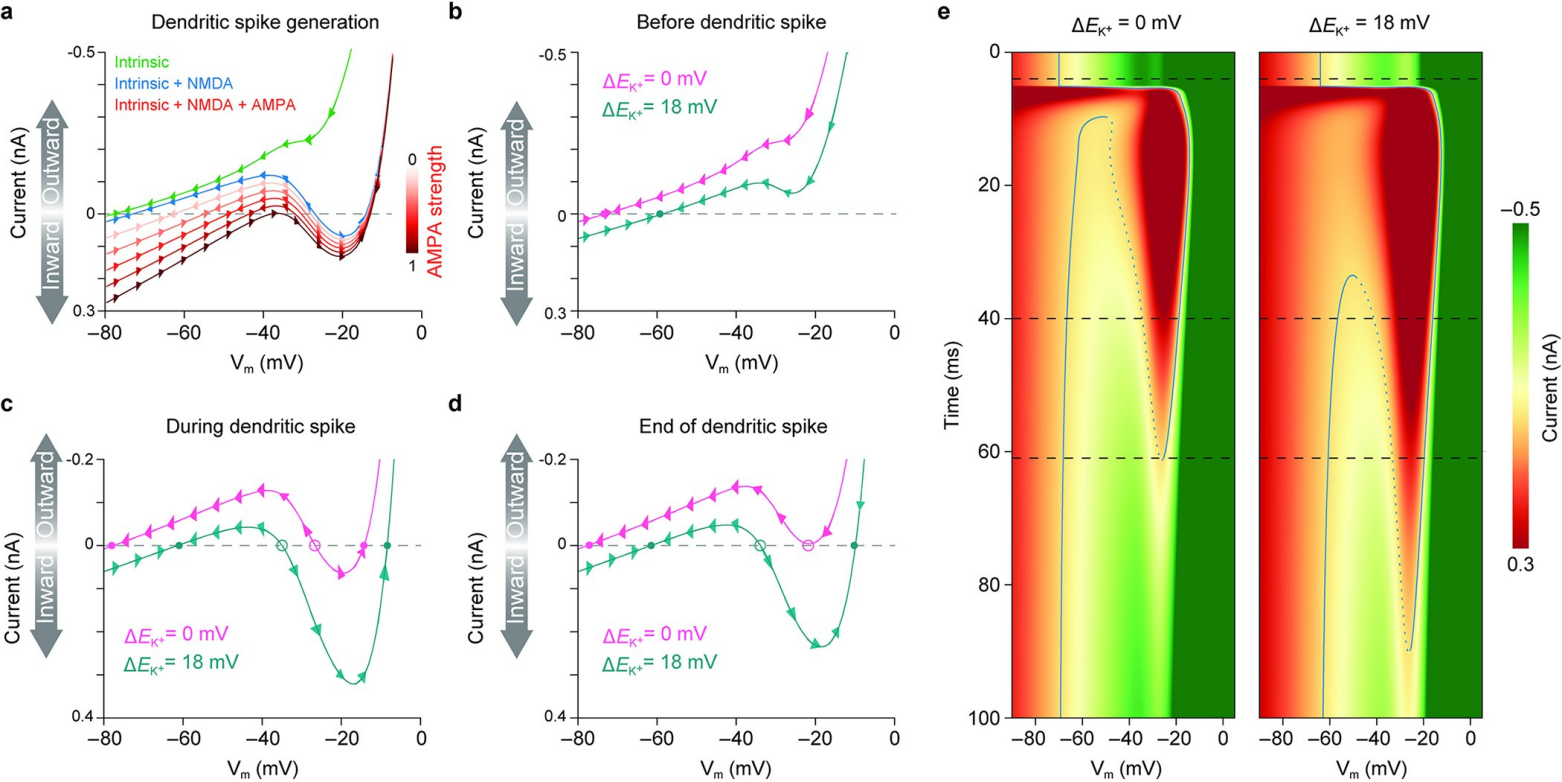
Current-voltage (I-V) relations and bistability with *E*_K_^+^ shift. (a) I-V curves during the generation of dendritic NMDA spike. Green: down-stable state with only intrinsic ion channels active. Blue: bistable state with intrinsic ion channels and NMDA receptor active. Red: Bi- and up-stable state with intrinsic ion channels and NMDA and AMPA receptors active. (b) Down-stable state before dendritic spike generation without and with *E*_K_^+^ shift. Solid points indicate stable fixed points. (c, d) Bistable states during and at the end of dendritic spike without and with *E*_K_^+^ shift. (c) Shows the hypothetical system with peak NMDA receptor conductance, without AMPA receptor activation, right before spike initiation, and (d) shows the system when outward and inward currents match for the system without *E*_K_^+^ shift around the end of the spike. Solid and open points indicate stable and unstable fixed points, respectively. (e) Heat maps showing the temporal evolution of the I-V landscape without and with *E*_K_^+^ shift. The corresponding I-V curves shown in (b–d) are indicated with black dotted lines, and full and dotted blue lines indicate stable and unstable fixed points, respectively. (a–c) Arrows indicate system flow direction. Note that positive inward current depolarizes the membrane, resulting in I–V plot similar to previous work (Major and colleagues [29]). See also **S1 Video**.

We then investigated the implications of the identified in **Fig 1** *E*_K_^+^ shifts might have on dendritic synaptic integration. We focused on dendritic segments with similarly tuned inputs as (1) we documented stimulus orientation-dependent and large (above 15 mV) *E*_K_^+^ shifts in these segments (**Fig 1g** and **1h**); and (2) previous work has shown that spatial clustering of co-active inputs is an important factor for dendritic spike initiation [32–38]. For this analysis, we implemented a biophysically detailed point model to simulate the V_m_ of a dendritic segment during synaptic input stimulation, referred to as a “point-dendrite” model due to its similarities with a conventional point-neuron model [70] (**Fig 2a**; see Methods). The point-dendrite model included an array of active ion channels found in the dendrites of V1, as well as AMPA and NMDA receptors [71–73] (**S1 Table**). The number of synapses and their orientation tuning were sampled as in **Fig 1** to simulate a dendritic segment receiving similarly tuned inputs. Synaptic inputs were activated by delivering a stimulation train consisting of 3 events in which AMPA and NMDA receptors were activated with a peak current depending on the synapse’s orientation tuning and with a Poisson-distributed delay (mean = 80 ms). This protocol induced V_m_ dynamics that reflected the stimulus orientation in the absence of *E*_K_^+^ shifts (**Fig 2b**). Importantly, for orientations close to the target orientation, we also observed regenerative activation of NMDA conductances, leading to NMDA-dependent dendritic spikes (see **Fig 3a**), while for the orthogonal orientation the V_m_ did not change (**Fig 2b**) [42]. To obtain a mechanistic understanding of how *E*_K_^+^ shifts might shape orientation tuning, we first tested their effect on dendritic spike properties (see Methods) for a stimulus presented at the target orientation, using different Δ*E*_K_^+^ values in the predicted range of **Fig 1h**, ([0–18] mV). *E*_K_^+^ shifts caused a broadening of dendritic spikes (**Figs 2c**, **2d and S1**); for example, for a 12 mV *E*_K_^+^ shift the duration of dendritic spikes increased by approximately 80% compared to no *E*_K_^+^ shift. This effect persisted in the presence of nonspecific inhibitory (GABA_A_) input (**S2 Fig**). Furthermore, the amount of excitatory synaptic drive needed to transition the dendritic V_m_ from subthreshold input summation to suprathreshold dendritic spiking decreased substantially with increased *E*_K_^+^ shifts (**Fig 2e** and **2f**).

In our model, 3 main parameters control dendritic spike generation: the number of dendritic synapses (N), the average synaptic activity (w), and the Δ*E*_K_^+^ (see Methods for details). By simulating different parameter configurations with dendritic spike occurrence as binary output measure, we fitted a function that describes the minimum Δ*E*_K_^+^ needed to trigger dendritic spiking given N and w (**S3 and S4 Figs**):

$$\Delta E_{K^{+}}(N,w)=aN+\beta w+\nu$$ (3)

Using Eq (3), we estimated the probability of generating a dendritic spike for a given stimulus orientation (**Fig 2g**). The probability of eliciting dendritic spikes rose drastically as a function of increasing *E*_K_^+^ shifts (**Fig 2g** and **2f**); for example, the probability of spiking to the target orientation increased from 48% to 97% when shifting the *E*_K_^+^ by 12 mV. This gain in the dendritic output was similar to the one expected from increasing the number of synapses (**S5 Fig**), with the main difference being that *E*_K_^+^ modulation is transient, selectively boosting the activity of repetitively active dendrites. Interestingly, the dendritic spiking orientation selectivity was largely constant even though the probability of spiking increased also for orientations away from the target orientation (**Fig 2g** and **2h**; Orientation selectivity index: 0.98, 0.98, and 0.96 for Δ*E*_K_^+^ = 0, 6, and 18 mV, respectively). Altogether, these results show that local K^+^ changes in dendritic segments with similarly tuned synaptic inputs prolong dendritic spikes and boost the probability of generating dendritic spikes without affecting their feature selectivity.

### *E*_K_^+^ shift alters the current-voltage attractor landscape in dendrites

To understand the general biophysical principles governing the regulation of dendritic spikes by local K^+^ changes, we turned to dynamical systems theory [74]. Dendritic spiking can be described by the instantaneous phase plot of the *V*_*m*_ and $\frac{{dV}_{m}}{dt}$. Given that the membrane capacitance is constant, we transformed this to the I-V curve using $\frac{{dV}_{m}}{dt}=-\frac{I}{C}$ [29]. With only the intrinsic ion channels active, V_m_ is attracted to a single down-stable hyperpolarized fixed point corresponding to the resting potential (**Fig 3a**). Inclusion of NMDA receptors changes the system to bistable, and an attractor basin together with a depolarized fixed point, corresponding to dendritic spiking, is created. The voltage barrier preventing the system from transitioning to an up-stable state, where V_m_ is only attracted to the depolarized fixed point, is overcome by the activation of AMPA receptors. As the activity of AMPA and NMDA receptors wears off, in conjunction with the high activity of K^+^ channels, the system is again pushed back to the down-stable state.

Shifting the *E*_K_^+^ alters the I-V curve attractor landscape in 3 fundamental ways (**Fig 3b–3e** and **S1 Video**). First, for the down-stable state, it moves the fixed point to a more depolarized V_m_, as well as reducing the net outward current across all V_m_ levels (**Fig 3b**). Second, for the bistable state, it lowers the voltage barrier needed to transition the system to the up-stable state and deepens the attractor basin (**Fig 3c and 3d**). Finally, by reducing the net outward currents at depolarized V_m_ levels, it prolongs the duration for which the system is in up- and bistable states (**Fig 3c–3e**). These dynamical system properties are all explained by the weakened K^+^ driving force through K^+^ channels and fully predict the Δ*E*_K_^+^-mediated effects we observed on dendritic spiking (**Fig 2**). Collectively, this analysis demonstrates that the effects of local K^+^ changes on synaptic integration can be predicted by the altered dynamical system properties of dendritic spikes.

### Local dendritic *E*_K_^+^ shifts induce neuronal firing gain modulation

**Fig 4:**
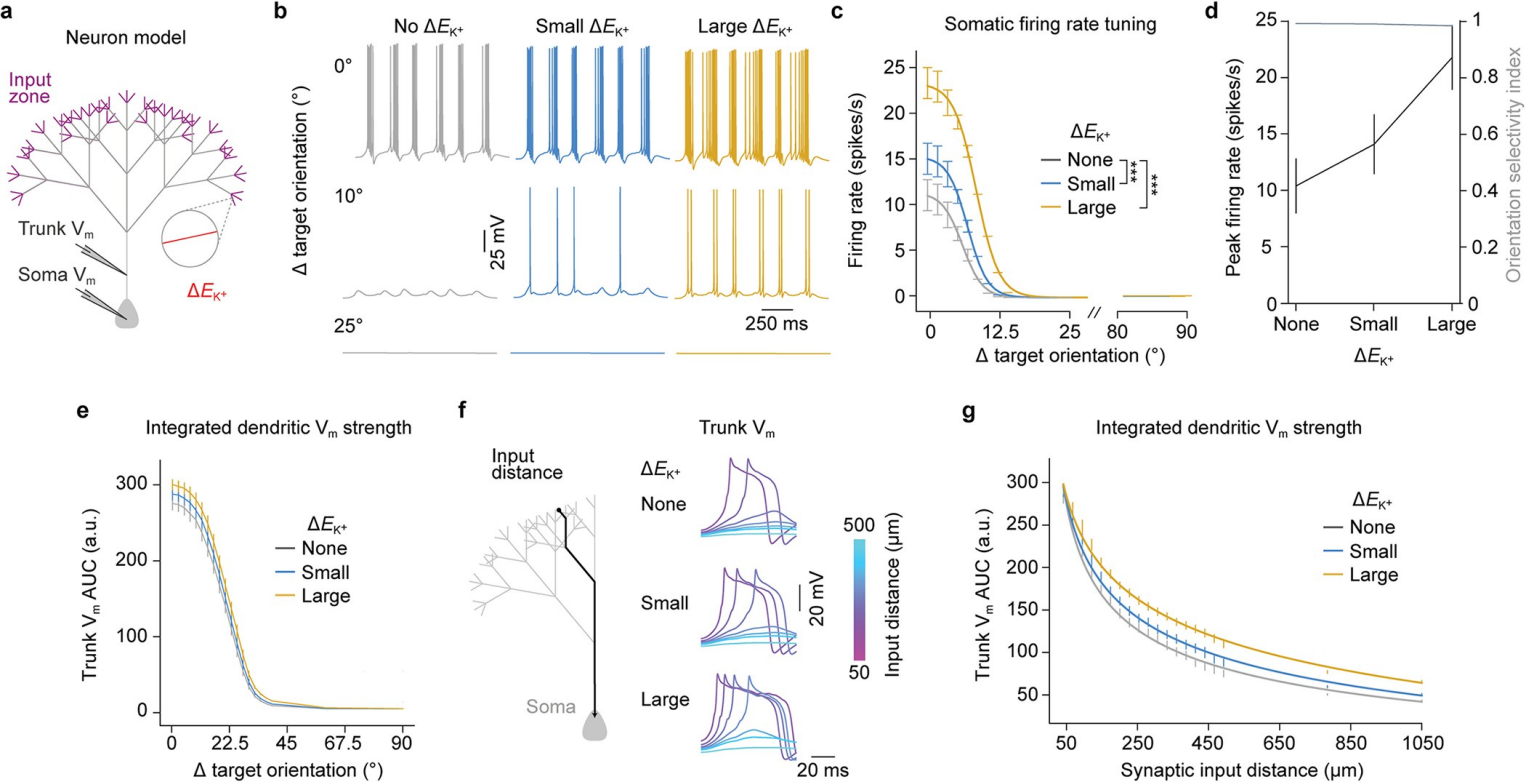
Local dendritic *E*_K_^+^ shifts induces neuronal firing gain modulation. (a) Diagram of the abstract neuron model. The neuron received orientation-tuned synaptic input at the distal dendrites and local *E*_K_^+^ shifts. (b) Example soma V_m_ traces obtained as a function of dendritic *E*_K_^+^ shift magnitude and stimulus orientation relative to target orientation. (c) Soma firing rate tuning curve for different dendritic *E*_K_^+^ shift magnitudes. Error bars are mean ± SEM. (*N* = 15 simulations). ***P = 10^−16^ and 10^−30^ for small and large *E*_K_^+^ shifts, respectively, one-tailed Student’s *t* test. (d) Soma peak firing rate at target orientation and orientation selectivity index as a function of dendritic *E*_K_^+^ shift magnitude. Error bars are mean ± SEM. (*N* = 15 simulations). (e) Area under the curve for the dendritic trunk V_m_ measurements as a function of stimulus orientation relative to target orientation and dendritic *E*_K_^+^ shift magnitude. Error bars are mean ± SEM. (*N* = 15 simulations). Trunk length was kept constant at 500 μm. Note that bAPs were here abolished by silencing voltage-gated sodium channels in the trunk and soma. (f) Example dendritic trunk V_m_ traces obtained at the target orientation as a function of synaptic input distance to soma and dendritic *E*_K_^+^ shift magnitude. (g) Area under the curve for the dendritic trunk V_m_ measurements at the target orientation as a function of synaptic input distance to soma and *E*_K_^+^ shift magnitude. Error bars are mean ± SEM. (*N* = 15 simulations) and solid lines represent fit to the function *f*(*x*) = *x*^*a*^+*c*. ***P = 0.61, 0.41, and 0.38 for different *E*_K_^+^ shifts. See also **S7 Fig** and **S2 Table**.

Our data suggests that the local [K^+^]_o_ increases in dendritic segments receiving similarly tuned inputs can boost the reliability and duration of dendritic spikes without compromising feature selectivity (**Fig 2g** and **2h**). Thus, we next sought to investigate the functional consequences this result might have on the somatic neuronal output. For this, we constructed an abstract neuron model using a fractal tree, mimicking the generic compartmentalization and fractal dimensionality of the apical dendritic tree of pyramidal neurons [75,76] and we stimulated distal dendrites with similarly tuned input [38,77,78] (**Fig 4a**). We simulated 3 conditions of *E*_K_^+^ shifts in the stimulated dendrites: no change, small shift, or large shift, based on the [K^+^]_o_ changes determined in **Fig 1** (**Fig 4a** and **4b**; see Methods). These 3 levels correspond to Δ*E*_K_^+^ = 0 mV, Δ*E*_K_^+^ = 6 mV, and Δ*E*_K_^+^ = 18 mV, respectively, to represent the case of no contribution as well as the minimum and maximum level used in (**Fig 1**), based on in vivo recordings. In S6 Fig, we also included a similar test using a reconstructed neuron morphology based on a previously published model.

These simulations revealed that somatic firing rates were significantly amplified when *E*_K_^+^ values were shifted locally (**Fig 4c** and **4d**; *P* = 0.007 and P = 0.003 for small and large *E*_K_^+^ shifts, respectively, one-tailed Student’s *t* test, *N* = 15 simulations). We determined that this form of gain modulation was consistent with a multiplicative transformation (**S7 Fig**; gain coefficient = 1.64 and 2.73 for small and large *E*_K_^+^ shifts, respectively), similarly to previously shown in the visual cortex [79]. This amplification was similar to the one expected by increasing the number of synapses (**S8 Fig**) with the main difference being that *E*_K_^+^ modulation closely follows the dendritic activity levels, operating in time scales of seconds. Importantly, and in congruence with what we observed in the dendrites (**Fig 2h**), the somatic firing selectivity was not changed by *E*_K_^+^ shifts despite increases in firing rate also for orientations away from the target orientation (**Fig 4c** and **4d**; orientation selectivity index: 0.99, 0.98, and 0.98 for no, small, and large *E*_K_^+^ shifts, respectively; *N* = 15 simulations). To elucidate how dendritic *E*_K_^+^ shifts cause dendritic gain modulation, we measured the area under the curve of the integrated V_m_ signal arriving at the base of the dendritic trunk during a stimulation event. To exclude back-propagating action potentials (bAPs), for this analysis, we turned off the voltage-gated sodium channels in the soma and trunk. This showed that the strength of the integrated dendritic signal, received by the soma, rose substantially with increasing *E*_K_^+^ shifts (**Fig 4e**); for example, the signal strength at the target orientation increased by around 12% when imposing large *E*_K_^+^ shifts compared to no *E*_K_^+^ shifts.

Finally, we asked what functional benefits there might be to this K^+^-mediated gain modulation. A major task of dendrites is to transmit incoming inputs to the soma for output generation. This is an inherent challenge, especially for distal inputs to large neurons, as the voltage signal tends to attenuate as a function of distance traveled and branching points [80,81]. We, therefore, speculated that one function of K^+^-mediated gain modulation might be to promote neurons transmitting signals over larger dendritic distances. To test this, we manipulated the synaptic input distance to the soma by scaling the entire neuron while measuring the integrated V_m_ signal at the base of the neuron (**Fig 4f and 4g**). Interestingly, due to the local gain of the synaptic input, the *E*_K_^+^-shifted regime is attenuated less with distance. This, in turn, makes it possible for signals to travel substantially longer, before reaching a similar integrated V_m_ signal. Based on the power-law fits using the χ^2^ fitting method for each Δ*E*_K_^+^ condition, we observe that as the neuron grows, the signal in the absence of *E*_K_^+^ shift is attenuated by a factor $∼\sqrt[{4}]{L}$ when compared to the large *E*_K_^+^ shifts (**Fig 4g**). Together, these results show that local activity-dependent [K^+^]_o_ increases and *E*_K_^+^ shifts in dendrites enhance the effectiveness of distal synaptic inputs to cause feature-tuned firing of neurons, without comprising feature selectivity.

## Discussion

We have developed mathematical formulations and biophysical models to address the open question of how local activity-dependent changes in [K^+^]_o_ affect dendritic integration of sensory-tuned synaptic inputs. Our work provides three major insights into this question. First, assuming activity-dependent [K^+^]_o_ changes in dendritic segments, the fine-scale arrangement of orientation-tuned synaptic inputs determines the magnitude of these changes; that is, segments with similarly tuned inputs can attain substantially higher [K^+^]_o_ increases than segments with diverse inputs. Second, these [K^+^]_o_ elevations in turn depolarize the *E*_K_^+^ which enhances dendritic excitability, increasing both the reliability and the duration of sensory-evoked dendritic spikes. Finally, these local dendritic effects promote gain amplification of neuronal input–output functions, resulting in increased somatic responsiveness without affecting the feature selectivity of the neuron. Our results, therefore, suggest a prominent and previously overlooked role for local activity-dependent changes in K^+^ concentration in regulating dendritic computations, shedding new light on the mechanisms underlying sensory integration in neurons.

Dendritic processing of synaptic inputs depends on the spatial and temporal organization of the inputs: spatially dispersed and asynchronous inputs are summed linearly, while inputs that are spatially localized and synchronous are summed nonlinearly and can facilitate the generation of dendritic spikes [32–38,82,83]. Here, we propose the novel idea that another important function of grouping co-tuned synaptic inputs close in space is to generate higher activity-dependent [K^+^]_o_ increases, creating local and feature-tuned dendritic “[K^+^]_o_ hotspots.” Such [K^+^]_o_ hotspots, potentially attaining up to 5 mM K^+^ increases relative to baseline (**Fig 1**), can markedly affect dendritic processing by dampening K^+^ currents. For example, we show here that they can reduce the amount of excitatory drive needed to trigger dendritic spiking, as well as broaden the dendritic spikes (**Figs 2** and **3**). Our results on dendritic integration are supported by previous work showing that K^+^ channels, highly expressed in dendrites [84], can modulate dendritic properties, albeit using artificial pharmacological or genetic disruption of dendritic K^+^ channels [43,44,84,85], or global [K^+^]_o_ elevations [82]. Here, we introduce the novel concept of activity-dependent, local [K^+^]_o_ changes that increase the excitability of dendrites and prolong dendritic spikes.

Regarding the functional role of the [K^+^]_o_-dependent dendritic integration modulation, we showed that [K^+^]_o_ increases can act as a volume knob and cause multiplicative gain modulation of the neuronal input–output function by boosting the effectiveness of orientation-tuned synaptic inputs (**Fig 4**). This makes the neuron more responsive and could contribute to increasing the signal-to-noise ratio of visual cortical neurons while, importantly, maintaining their orientation selectivity. Compared to long-term plasticity mechanisms, [K^+^]_o_-dependent dendritic integration modulation requires minimum usage of resources and is transient, operating in time scales of seconds, selectively boosting the activity of repetitively active dendrites; as such, [K^+^]_o_ changes closely follow the overall dendritic activity levels. Indeed, we have previously shown that [K^+^]_o_ changes are state dependent, e.g., on whether the animal is in quiescence or locomoting [14], indicating that state-dependent modulation of sensory processing may be supported by [K^+^]_o_ changes following the overall activity levels. In addition, the broadening of dendritic spikes could potentially also enhance the capacity of the neuron to integrate temporally delayed excitatory inputs [86] and act as a cellular mechanism involved in short-term memory. Notably, the [K^+^]_o_—dependent increase in the reliability of dendritic spikes is of potential high functional importance as dendritic spikes are critical for in vivo learning and behavior [87]. For example, the dendritic response to inputs arriving on the apical dendrites of cortical neurons has been implicated in contextual modulation of sensory processing and perceptual sensitivity [88–90]. A recent report suggests that densely localized thalamic inputs to apical dendrites might regulate such modulation by facilitating dendritic spikes [91]. It is thus tempting to speculate that local activity-dependent [K^+^]_o_ increases of spatially clustered similarly tuned inputs to apical dendrites effectively modulate cortical sensory processing, by means of promoting dendritic spikes. In this context, it is important to note that while we chose here to use visual orientation tuning as our framework of study, our proposed mechanistic concept is agnostic to sensory features, brain regions, or animal species. The fundamental requirement for the emergence of dendritic [K^+^]_o_ hotspots is that synaptic inputs are activated on a spatially and temporally synchronized scale. Hence, this mechanism could play a role in dendritic computations in diverse brain regions such as the hippocampus, motor cortex, visual cortex, and somatosensory cortex where spatiotemporally synchronized inputs have been observed [32,42,45–50].

Importantly, our prediction on the existence of the local activity-dependent [K^+^]_o_ hotspots can be potentially experimentally tested. The gold standard for measuring [K^+^]_o_ dynamics in the brain is with K^+^-selective microelectrodes [5,7–10,12–14,65]. However, this technique creates a dead space surrounding the electrode, and can only measure from a single point in space, hence providing poor spatial resolution. Instead, to test our hypothesis, an optical approach like two-photon microscopy with fine-scale synaptic resolution seems ideal. Genetically encoded green fluorescent protein-based [K^+^]_o_ sensors exist [92], which could be combined with a red-shifted genetically encoded voltage indicator [93] to simultaneously monitor [K^+^]_o_ dynamics and synaptic synapse V_m_ tuning in vivo. We hope that experimentalists in the future will use these advanced techniques to probe the existence of local dendritic [K^+^]_o_ changes, as well as their functions and the cellular mechanisms regulating them, such as astrocyte-mediated K^+^ uptake [94].

Our approach also comes with its limitations: First, while we focused only on the postsynaptic impact of local [K^+^]_o_ changes, it is reasonable to predict that activity-dependent [K^+^]_o_ hotspots could also affect the presynaptic terminals [18]. Increases in [K^+^]_o_ depolarize axons, which can broaden action potentials and increase presynaptic calcium entry [95,96], leading to enhanced glutamate release and stronger synaptic transmission. Such amplification of local synaptic integration, through pre- and post-synaptic mechanisms, could play important role in neuronal circuit development and long-term potentiation by supporting spike timing-dependent plasticity [97,98]. Second, [K^+^]_o_ changes may modulate the gating properties of specific types of K^+^ channels, such as the inward-rectifier K^+^ (Kir) [99] or the slow delayed rectifier (KCNQ1) [100,101], potentially providing another venue for [K^+^]_o_ self-regulating mechanism of dendritic excitability. Third, synapses surrounding the dendritic segments may also participate or be affected by the local [K^+^]_o_ changes: activity of the surrounding synapses, belonging to dendritic branches of the same or different neurons may also contribute to the local [K^+^]_o_. This co-activation of nearby dendrites could either increase the noise levels or locally promote the synergy between synapses, dendrites, and neurons. Fourth, in this work, we did not assess in detail GABAergic inhibition, known to play important roles in regulating dendritic activity [102,103]. Future work could address if local [K^+^]_o_ increases near dendritic segments with similarly tuned excitatory inputs could cause disinhibition by reducing the inward driving force for chloride ions thus increasing neuronal feature selectivity or creating a temporal window for dendritic plasticity. Fifth, the width of the extracellular space can be highly heterogeneous, ranging from 0.04 to 0.5 μm [63,104], yet smaller than the pyramidal neuron dendritic diameter [105,106]. Importantly, the extracellular space itself is a dynamic compartment with timescales ranging from seconds to hours; for example, the extracellular space decreases in response to epileptiform activity [107] and these structural changes can be highly local [63], making the extracellular space size another mean for local modulation of dendritic excitability. Finally, our mean field approximation quantifies average [K^+^]_o_ changes after synaptic activation and thus lacks temporal resolution. We chose this approach because several important parameters have yet to be experimentally determined, including the voltage-dependent fraction of K^+^ currents of total NMDA currents and the precise geometry of the extracellular space surrounding functionally mapped dendritic segments, which prevents precise simulations of time-dependent [K^+^]_o_ dynamics. We assumed here that the system goes asymptotically towards the well-mixed state following synaptic activation, and we estimate that this is reached approximately 150–170 ms after input onset, but we cannot infer the nature of the spatiotemporal [K^+^]_o_ dynamics before this time point. In the future, experimental knowledge about these parameters would enable us to make a full-scale electrodiffussive model of the extracellular space, improving the temporal resolution of extracellular ion dynamics.

In conclusion, our study is a first step towards unraveling the role of extracellular K^+^ changes in dendritic integration. Future theoretical and experimental studies are needed to obtain a comprehensive understanding of how local activity-dependent ionic shifts contribute to information processing and computations in dendrites.

## Methods

### Sampling of orientation-tuned synapses of dendritic segments

We defined a dendritic segment as a cylinder with length L = 10 μm and radius R_1_ = 1 μm [32,45–47]. Each segment was randomly populated with 7 to 13 synapses, yielding synaptic densities comparable to that of neocortical neurons [51–53]. The orientation preference of individual synapses of a dendritic segment depended on the synaptic organization type to which the segment belonged, that is, the diverse or similar input tuning regime. Synapses in segments with similar tuning were assigned an orientation preference using a half-circular normal distribution with σ = 15° [42,72,108] and a mean target orientation arbitrarily set to 0°, while synapses in segments with diverse tuning were assigned orientation preferences using the uniform distribution. Importantly, for both types of input regimes, we could reproduce the synaptic orientation preference cumulative distributions measured previously in the visual cortex of ferrets [42]. Moreover, the orientation preferences of our diverse tuning regime resembled those previously recorded in the visual cortex of mice [45,54]. All synapses, irrespective of the spatial organization regime, had a tuning curve width given by a half-circular normal distribution with σ_tuning_ = 11° [42,55], and the activity of each synapse spanned from 0 to 1 (**Fig 1c**). This sampling method for the number and tuning properties of excitatory synapses of dendritic segments was implemented throughout this work.

### Synaptic activity factor of dendritic segments

We modeled the activation of synapses of dendritic segments by assuming that all synapses were activated by visual stimulation according to their tuning curve. To assess the expected synaptic activity level of a segment for a given stimulus orientation, we calculated the average activity of all synapses in that segment, w(θ), that depends on the orientation tuning of each synapse and the distribution of orientation preferences in the dendritic segment (**Fig 1d and 1e**). We then obtained the synaptic activity factor by comparing the expectation values from the synaptic activity distributions for the 2 types of dendritic segments. We sampled 10^4^ dendritic segments and used a Gaussian kernel density estimator to determine the probability density function p(x) for the expected synaptic activity distributions for each segment type. The expectation value was calculated as ***E***(*X*) = ∑_*i*_*x*_*i*_*p*_*i*_ where x was a linearly spaced set of values between 0 and 1. To investigate the stimulus orientation-dependent modulation of the synaptic activity factor (**Fig 1f**), we repeated this procedure for all stimulus orientations in the interval θ ∈ [0: 90°].

### Extracellular space surrounding the dendrite segment

The exact size and shape of the extracellular space surrounding functionally mapped, short dendritic segments is currently unknown, so we chose to model it as a cylinder that encapsulates the dendritic segment cylinder. The space in between these 2 cylinders thus constitutes the extracellular space in our investigations, and its volume (V_ext_) is given by:

$$V_{ext}=\pi L(R_{2}^{2}-R_{1}^{2})$$ (4)

With R_1_ and R_2_ being the radius of the inner and outer cylinder, respectively, both with length L = 10 μm. This can be rewritten to express the volume ratio (V_R_) as the ratio between the 2 cylinders:

$$V_{R}=\frac{V_{int}}{V_{ext}}=\frac{{R_{1}}^{2}}{R_{2}^{2}-R_{1}^{2}}$$ (5)

For simplicity, synapses were considered as points on the surface of the inner cylinder, and hence, the volume of a synapse was considered part of the dendritic segment volume.

### K^+^ diffusion state

During synaptic excitatory transmission, K^+^ ions are released into the extracellular space primarily through NMDA receptors [16,21,26,27] and move around due to diffusion, although their movement is hindered by various cellular elements within the extracellular space. To account for this hindrance, we define effective diffusion as a function of tortuosity [66]:

$$D^{*}=\frac{D_{K_{free}^{+}}}{\lambda^{2}}=\frac{1.96\frac{{\mu m}^{2}}{ms}}{1.6^{2}}=0.76\frac{{\mu m}^{2}}{ms}$$ (6)

Here, $D_{K_{free}^{+}}$ is the diffusion of free *K*^+^ ions in physiological saline, *λ* is a non-dimensional measure of how hindered an ion is in its movement in a given space, and D* is the resulting diffusion rate. The parameter for $D_{K_{free}^{+}}$ and *λ* were based on previous work [57,66,109,110]. Here, we assume that diffusion in the radial direction is negligible, as the extracellular space is very small compared to the dendritic segment length. Moreover, we assume closed boundary conditions on the outer cylinder wall, since the extracellular space mainly contacts cellular membranes of neighboring neurons and/or glial cells. Finally, assuming the extracellular K^+^ behaves like a brownian particle, the spatial diffusion of the K^+^ will extent as a multivariate normal distribution with standard deviation $\sigma =\sqrt{{2D}^{*}\tau }$ [111,112]. This equates the average distance traveled for an individual particle, after a certain characteristic time *τ*. Using this equation, we can likewise compute the time it will take for a particle to diffuse at a distance L:

$$L=\sqrt{{2D}^{*}\tau }\to \tau =\frac{L^{2}}{{2D}^{*}}$$ (7)

After this characteristic time *τ*, we assume that the [K^+^]_o_ is well-mixed and uniform throughout the extracellular space. For the dendritic segment with length L = 10 μm, the characteristic time becomes *τ* ≈ 66 ms. Considering that the majority of K^+^ efflux occurs within ≈ 100 ms from initial synaptic activation, we predict a peak [K^+^]_o_ elevation along the dendritic segment after ~150–170 ms, for a *τ* ≈ 66 ms. Following, pumps and channels return the K^+^ levels to baseline, yet operating on much lower timescales as seen in in vitro experiments [58,59] (see **S2 Text** and **S9–S13 and S15 Figs** for simulations that describe the spatial and temporal properties of K^+^ diffusion in the presence of Na^+^/K^+^ pumps). Thus, we analyzed our models with fixed [K^+^]_o_ changes, with values chosen as described in the section below. This time scale separation, due to the different time constants of the described phenomena, has been successfully applied previously on similar questions [113]. Finally, we assume that the boundary between the dendritic segments is closed, implying a finite ion concentration within the segment. This limits our approach since we do not investigate the effect of spillover K^+^ ions moving along the dendrite, potentially affecting neighboring segments. However, given the average distance traveled for a K^+^ ion between the stimuli considered here (based on Eq 7, the expected movement is ≈ 30 μm during a period of 600 ms), only the directly neighboring segments could potentially be affected, thus keeping the ionic shift highly localized (**S10 Fig**).

### Activity-dependent K^+^ change across the neuronal membrane

To estimate synaptic activity-dependent [K^+^]_o_ changes near dendritic segments, we assumed that local increases in [K^+^]_o_ are linearly correlated with the expected synapse activity, as a result of K^+^ efflux from the intracellular space. By scaling the K^+^ change in the diversely tuned input regime with the stimulus orientation-dependent synaptic activity factor, we obtained an estimate of the K^+^ change in the similarly tuned regime. Using this, in conjunction with Eq 5, we derived equations for the 2 types of input regimes:

$$\Delta {\left[{Diverse\;K}^{+}\right]}_{o}(\theta ,\Delta [K^{+}{]}_{i})=|\Delta [K^{+}{]}_{i}|V_{R}$$ (8)

$$\Delta {\left[{Similar\;K}^{+}\right]}_{o}(\theta ,\Delta [K^{+}{]}_{i})=E(\theta )\Delta {\left[{Diverse\;K}^{+}\right]}_{o}(\theta ,\Delta [K^{+}{]}_{i})$$ (9)

With Δ[K^+^]_o_ and Δ[K^+^]_i_ being the change in extracellular or intracellular [K^+^], respectively, V_R_ the volume ratio as defined in 5 and **E**(θ) being the stimulus orientation-dependent synaptic activity factor. Using the Nernst equation, we then can express the shift in *E*_K_^+^ (see Eq 2).

### Point-dendrite model

We simulated the dendritic segment V_m_ as an isolated resistance-capacitance circuit, similar to the common practice for point-neuron models [70]. The major differences between point-neuron models and the point-dendrite model developed here stem from the specific ion channel setup and cellular resistance, chosen to mimic dendritic properties [73] (**S1 Table**). The circuit is given by the ordinary differential equation:

$$\frac{{dV}_{m}}{dt}C_{m}=I_{ext}-AI_{int}$$ (10)

*C*_*m*_ Is the membrane capacitance, set to 2 $\frac{\mu F}{{cm}^{2}}$ to simulate the contribution from the spines [114], *I*_ext_ is the current generated by synaptic receptors (AMPA and NMDA receptors), and *I*_int_ is the current generated by intrinsic ion channels (K_leak_, Na_V_, K_V_, K_M_, K_A_, K_Ca_, C_aV_, and HCN). The latter is multiplied by the surface area, *A*, as these channels are scattered uniformly along the surface of the dendrite. All relevant channel dynamics and specific parameters are included in the S1 Text. The system was simulated using an exponential Euler scheme (cnexp):

$$V_{m}(t+dt)=V_{\infty }(t)+(V_{m}(t)-V_{\infty }(t))exp\left(\frac{-dt}{{\tau_{V}}_{m}(t)}\right)$$ (11)

Here, *V*_∞_ is the solution to the equation $\frac{{dV}_{m}}{dt}=0$:

$$V_{\infty }(t)=\frac{{\bar{g}}_{L}E_{L}+{\bar{g}}_{Nav}{m^{3}hE}_{Na}+{\bar{g}}_{Kv}nE_{K^{+}}+{\bar{g}}_{KA}{nlE}_{K^{+}}+{\bar{g}}_{KCa}{nE}_{K^{+}}+{\bar{g}}_{Cav}m^{2}{hE}_{{Ca}^{2+}}+{\bar{g}}_{HCN}{mE}_{HCN}+\sum g_{syn}E_{syn}}{{\bar{g}}_{L}+{\bar{g}}_{Nav}m^{3}h+{\bar{g}}_{Kv}n+{\bar{g}}_{KA}nl+{\bar{g}}_{KCa}n+{\bar{g}}_{Cav}m^{2}h+{\bar{g}}_{HCN}m+\sum g_{syn}}$$ (12)

Where $\bar{g}$ denotes a channel’s average conductance, *n*^*x*^ is from the individual channel dynamics controlling the gating and channel-specific conductance, *E*_*ion*_ denotes the relevant reversal potential and $\sum g_{syn}E_{syn},for\;syn\in AMPA,NMDA,GABA_{A}$ sums the current from the different types of synaptic receptors, were $g_{syn}={\overset{-}{g}}_{syn}(B-A)f,with\;f=Mg_{block}(V_{m})if\;syn==NMDA,and\;f=1\;otherwise$. For complete synaptic equations for NMPA, NMDA, and GABA_A_ receptors, see **S1 Text**. The membrane time constant is given as follows:

$$\tau_{V_{m}}=\frac{C_{m}}{g_{m}}$$ (13)

Here, *g*_*m*_ is the total membrane conductance ($g_{m}={\bar{g}}_{L}+{\bar{g}}_{Nav}m^{3}h+{\bar{g}}_{Kv}n+\ldots$).

The point-dendrite model was populated with orientation-tuned synapses as described in the previous section. The orientation-tuned synaptic activity in the point dendrite was implemented by multiplying g_AMPA_ and g_NMDA_ of synapses with the average synaptic activity, w(θ), that depends on the orientation tuning of each synapse and the distribution of orientation preferences in the dendritic segment, according to **Fig 1d and 1e**. For each stimulation presynaptic event, all synapses were activated with a Poisson distributed delay (λ = 80 ms). The orientation tuning of the individual synapses was achieved by scaling the AMPA and NMDA conductance according to the synapse’s tuning curve given by a half-circular normal distribution with σ_tuning_ = 11° (see **Fig 1c**) [42,55,72,108]. To create a stimulation train consisting of several input events, we replicated the synaptic activation 2 times with a constant delay of 300 ms in between events. Based on our analysis in the Methods section “K^+^ diffusion state,” the **S2 Text**, and the **S9–S13 Figs**, we modeled the activity-dependent local increase in [K^+^]_o_ a step function in between events: The first stimulation event was regarded as the control condition with Δ*E*_K_^+^ = 0 mV, and the 2 subsequent events as the experimental condition for a given *E*_K_^+^ shift in the interval Δ*E*_K_^+^ ∈ [6:18 mV]. We used a V_m_ of –30 mV as a threshold criterion for identifying dendritic spikes. This value was chosen because we observed the largest change in V_m_ around this value, as synaptic integration transitions from subthreshold input summation to suprathreshold spiking. To evaluate the effect of nonspecific inhibitory (GABA_A_) input, we repeated our point-dendrite model simulations in the presence of inhibition. Specifically, we simulated the activation of GABA_A_ synapses randomly activated at 10 Hz (**S2 Fig**).

### Linear dendritic spike function

To investigate the relationship between the average synaptic activity, w(θ), (**Fig 1e**), the Δ*E*_K_^+^, and the number of synapses (N) for the emergence of a dendritic spike, we independently varied each variable in small increments in the point-dendrite model. By doing so, we identified the lowest value of Δ*E*_K_^+^ that was able to push the V_m_ over the threshold of –30 mV, our working criterion for dendritic spike generation, given N and w. We assumed that the relation between the 3 parameters was linear, and we, therefore, fitted a plane to obtain a model of Δ*E*_K_^+^ needed to trigger dendritic spikes as a function of N and w. For this, we used the planar equation αw + βN + ν = 0 and solved it as Ax = b (**S3** and **S4 Figs** and Table 1 for coefficients):

$$\left(\begin{array}{c} \alpha \\ \beta \\ \nu \end{array}\right)={\left({AA}^{T}\right)}^{-1}A^{T}b$$ (14)

**Table 1:** Fitted coefficients for dendritic spike emergence seen in S3 Fig.

Coeff.	α	β	ν
Fitted value	−38.43	−2.08	52.32

From this equation, we can see that adding 1 additional synapse would lower the required Δ*E*_K_^+^ for a dendritic spike by 2.08 mV. Accordingly, a 10% increase in w would lower the required Δ*E*_K_^+^ for a dendritic spike by 3.8 mV. Note that in this work w varies between 0 and 1 and the maximum number of synapses on a dendritic segment is 13 [51–53]. A combination of sufficiently high number of synapses N and w would lead to negative Δ*E*_K_^+^, in which case we assumed to correspond to no *E*_K_^+^ shift. From this, we could then estimate the dendritic spike probability as a function of Δ*E*_K_^+^ and stimulus orientation. Using statistical formalism for the setup of the dendritic segments with similarly tuned inputs, we sampled the number of synapses and synaptic orientation preferences of 10^4^ segments, and calculated the average synaptic activity, w(θ). Following, we determined the Δ*E*_K_^+^ needed to generate a dendritic spike according to Δ*E*_K_^+^ (N, w) = αN + βw + ν for each stimulus orientation. For a stimulus presented in the target orientation, we compared this value with a set of Δ*E*_K_^+^ values in [0, 6, 10, 18 mV] to determine whether each sampled segment fired a dendritic spike or not. To obtain the set Δ*E*_K_^+^ values for the other orientations, we multiplied the set Δ*E*_K_^+^ values of the target orientation with the normalized version of the synaptic activity factor seen in **Fig 1f** and repeated the comparison with the respective needed Δ*E*_K_^+^ value. Finally, we calculated the fraction of dendritic segments, at each orientation, that would be expected to show dendritic spikes and converted this into a probability.

### Current-voltage attractor landscape

To understand the biophysical principles governing the regulation of dendritic spikes by local K^+^ changes, we used dynamical systems theory [74]. While our point-dendrite model is defined by the differential Eq 10, it is not possible to solve it analytically. Rather, we chose to plot the exact solutions to the differential equations evaluated at different V_m_ as a vector plot, which in essence is similar to summing all I-V curves from the included intrinsic and synaptic channels. We thus have V_m_ on the first axis, and on the second we have its derivative which in our case is denoted $\frac{{dV}_{m}}{dt}=-\frac{I}{C}$. The time evolution of the synaptic currents in the point dendrite model was described as before (see S1 Text). Synaptic receptors were simulated in the time interval [0: 100 ms] using the Euler method. For each time step, an I-V curve was generated and the resulting I-V curves were concatenated to create a time-dependent I-V landscape (**Fig 3e** and **S1 Video**). To compare the effect of [K^+^]_o_ changes, we simulated the system with Δ*E*_K_^+^ = 0 mV and Δ*E*_K_^+^ = 18 mV.

### Abstract neuron model

Using the NEURON simulation environment, we constructed an abstract neuron model to systematically test the role of local [K^+^]_o_ elevations on dendritic input integration and somatic output firing. The dendrite’s morphology was constructed as a fractal tree (**Fig 4a**): for each dendritic section generation, 3 new sections, each with half the length of the former, were added (length of the most distant section: 20 μm). This gave a fractal with dimension D = ln 3/ ln 2 = 1.58, mimicking the generic compartmentalization and fractal dimensionality of the apical tree of pyramidal neurons [75,76]. The fractal dendritic tree was extended with a trunk dendritic compartment (500 μm) and a soma (length 30 μm, diameter 10 μm) to complete the neuron morphology, and intrinsic ion channels (K_leak_, Na_P_, Na_T_, K_P_, K_T_, K_DR_, K_Ca_, KM, Ca_LVA_, Ca_HVA_, and HCN) were inserted into the cellular compartments (**S2 Table**), based on [73]. Using this morphology enabled us to test the effect without hidden edge cases caused by morphology, as regardless of the placement of the cluster the effect should remain the same. The fractal dimension kept the properties of the neuron as close as possible to that of a detailed morphology.

To investigate the compartmentalization of dendritic integration under the different Δ*E*_K_^+^ values, we stimulated synapses residing in segments with similar orientation tuning on the most distant dendritic branches [38,77,78]. For this, a Poisson-distributed number of dendritic segments (λ = 32 segments) were selected to host the similarly tuned inputs in each simulation iteration, and the number of synapses and their orientation tuning were sampled as described above. Only distal dendrites received direct input, to ensure a constant distance to the soma. The local Δ*E*_K_^+^ of stimulated dendrites were grouped into 3 conditions: no shift (Δ*E*_K_^+^ = 0 mV), small shifts (Δ*E*_K_^+^ = 6 mV), or large shifts (Δ*E*_K_^+^ = 18 mV), based on the [K^+^]_o_ changes found in **Fig 1**. As before, the orientation tuning of individual synapses was achieved by scaling the AMPA and NMDA conductance. To simulate a realistic somatic firing pattern, the synapses within each dendritic segment were activated randomly given by a Poisson distribution with λ = 30 ms. This stimulation protocol was repeated 3 times with a 300 ms delay between stimulation events. Each neuron setup was simulated with each of the 3 Δ*E*_K_^+^ conditions to directly assess the effect of the local K^+^ changes.

### Multiple ionic contributions

In this investigation, we only considered the activity-induced changes of [K^+^]_o_ and assumed the concentration of all other ions to remain constant. We assume contributions from Na^+^ and Ca^2+^ to be minimal and their effects covered within the range of the *E*_K_^+^ demonstrated. This difference in the respective contributions arises from the ratio between the intra and extracellular volume, where the former is assumed to be at least a factor of 2 larger [62–64] (the size of the extracellular space ranges from 0.04 to 0.5 μm [63,104]), and is smaller than the pyramidal neuron dendritic diameter [105,106] and is also motivated by our previous experimental recordings documenting in vivo elevation of [K^+^]_o_ [14]. This results in greater changes seen in the extracellular space, and combined with the formulation of the Nernst equation, the shift in *E*_K_^+^ outweighs the other ions. For a more in-depth discussion about this, see **S3 Text** and **S14 Fig**.

### Neuronal activity measures

To obtain a better understanding of how the local [K^+^]_o_ changes impact the activity of the neuronal model we introduced 2 additional measures, in addition to the conventional measurement of somatic firing rate: Neuronal firing gain transformation and area under the curve (AUC) for the integrated dendritic V_m_ signal.

### Neuronal firing gain transformation

We assumed that the gain modulation of the somatic firing tuning curve could be described by a multiplicative or additive function, similar to previously described in the visual cortex of mice [79]. The multiplicative and additive functions are given by:

$$FR_{After}(\theta )=\xi_{mul}\;FR_{Before}(\theta )$$ (15)

$$FR_{After}(\theta )=\xi_{add}\;FR_{Before}(\theta )$$ (16)

Here, *FR*_*Before*_(*θ*) and *FR*_*After*_(*θ*) are the somatic firing rates at a given orientation with non-shifted or shifted *E*_K_^+^ values, respectively, ξ is the fit parameter that describes the change in firing rate either as a gain coefficient or as a gain constant for the multiplicative and additive gain transformation, respectively. To determine the fit parameters, we fitted the 2 functions to the firing rate tuning curve data using the χ^2^ fitting method (**Table 2** and **S7 Fig**). Only the multiplicative method was able to produce a satisfying fit and capture the main features of the tuning curve, whereas the additive only captured the responses to orientations close to the target orientation.

**Table 2:** Fitted gain parameters. Gain parameters obtained for the multiplicative (top) and additive functions (bottom) for the small and large *E*_K_^+^ shifts (left and right, respectively). Errors were assumed Gaussian and reported as standard deviations. See also **S7 Fig**.

	^ξ^ Small Δ*E*_K_^+^	^ξ^ Large Δ*E*_K_^+^
Multiplicative Additive	1.64 ± 0.21 2.73 ± 0.75	2.73 ± 0.32 7.06 ± 0.33

### Area under the curve

To understand how dendritic *E*_K_^+^ shifts shape dendritic gain, we computed the AUC of the V_m_ measured at the base of the dendritic trunk, over an interval of 500 ms to capture the full synaptic activation during an event. To exclude the contribution of bAPs, we here turned off the voltage-gated sodium channels in the soma and trunk, which eliminated somatic firing. To calculate the AUC, we first subtracted the V_m_ baseline of the control signal, corresponding to Δ*E*_K_^+^ = 0 mV, from all traces, and used the trapezoidal method.

### Orientation selectivity index

The orientation selectivity index was computed as 1 –circular variance (CV) in orientation space given by:

$$Orientation\;selectivity\;index=1-|CV|=\left|\sum_{k}r_{k}\frac{e^{-{i2\theta }_{k}}}{\sum_{k}r_{k}}\right|$$ (17)

Where *r*_*k*_ is a measurable output at orientation *θ*_*k*_ (in radians); here, we used dendritic spiking probability and somatic firing rate as output measures. To compute OSI, we mirrored the results obtained in the simulation between [0:90°] to obtain values in the interval [−90:90°].

### Statistics

To determine if somatic firing rates, obtained with either small or large local *E*_K_^+^ shifts, differed from firing rates obtained with no *E*_K_^+^ shift, we performed one-tailed Student’s *t* tests on the residuals of the somatic firing rates. *P* < 0.05 was considered statistically significant, where *P < 0.05, **P < 0.01, and ***P < 0.001.

## Supporting information

S1 Text Biophysical model setup. (PDF)

S2 Text Spatial and temporal properties of K^+^ diffusion. (PDF)

S3 Text Electrochemical potential shifts of ions. (PDF)

S1 Fig Voltage response of the point dendrite with excitatory inputs. Example dendrite V_m_ traces for a similarly tuned dendritic segment as a function of stimulus orientation relative to target orientation. Individual trials are in gray and average is in teal. The colored segments in the inset (right) show the impact of the *E*_K_^+^ shift (comparison of the first and third responses). The first stimulation event induces a small *E*_K_^+^ shift (10 mV) for the target orientation. For the rest of stimulus orientations, the shift in *E*_K_^+^ is scaled according to **Fig 1f**. *E*_K_^+^ shifts increase dendritic spike occurrence and dendritic spike duration.
(PDF)

S2 Fig Voltage response of the point dendrite with excitatory and inhibitory inputs. Top 2 plots: Example dendrite V_m_ traces for different orientations, as in S1 Fig, when GABA_A_ synapses (0.2 synapses/μm density) were randomly stimulated with mean Poisson frequency 10 Hz. Following plots show example dendrite V_m_ traces for target orientation with increasing the inhibitory conductance and mean Poisson frequency of inhibitory activation. In each subplot with gray are voltage responses from 10 repetitions, and in teal is the mean response. The colored segments in the inset show the impact of the *E*_K_^+^ shift (comparison of the first and third responses). As in S1 Fig, the first stimulation event induces a small *E*_K_^+^ shift (10 mV) for the target orientation. High *E*_K_^+^ shifts increase dendritic spike occurrence and dendritic spike duration in the presence of nonspecific inhibition. High enough inhibition (bottom panel) reduces dendritic spike occurrence irrespective of the *E*_K_^+^ shift.
(PDF)

S3 Fig Dendritic spike emergence plane fit. We created multiple input–output curves by varying the average synaptic activity (w), the number of synapses on the dendritic segment (N), and Δ*E*_K_^+^, in the point-neuron model, while having dendritic spike occurrence (V_m_ above –30 mV) as the output measure. This was done multiple times for each parameter combination and the resulting scatter of points in 3D space is shown here (blue circles). Following, we fitted a plane to this data (black grid), as we assumed the relation between w, N, Δ*E*_K_^+^ and dendritic spike generation to be linear; points on the plane denote the parameter sets which were able to generate a dendritic spike.
(PDF)

S4 Fig Dendritic spike emergence for constant N. Data similar to S3 Fig, presented here as a scatter plot for different numbers of synapses, N (colors). As suggested by the planar plot, the relationship between Δ*E*_K_^+^ and w is well approximated by a linear relationship. In addition, the slope of each line increases linearly with the number of synapses.
(PDF)

S5 Fig Synaptic activity and number of synapses regulate active dendritic properties. Heat map showing peak V_m_ depolarization as a function of mean synaptic activity (w) and the number of synapses (N) in the point dendrite model. Dotted lines indicate the transition to dendritic spiking. Computed for Δ*E*_K_^+^ = 0 mV. Compare this figure to Fig 2f of main text.
(PDF)

S6 Fig Orientation tuning of a layer 5 pyramidal cortical neuron, at different Δ*E*K_K_^+^. Somatic firing rate tuning curve for different dendritic *E*_K_^+^ shift magnitudes, similar to Fig 4. To further verify the results obtained with the abstract neuron of Fig 4, we simulated a neuron with detailed, reconstructed morphology. Here, we chose the widely used model of a layer 5 pyramidal neuron, obtained from [1]. The neuronal setup was kept similar to the one used for the abstract neuron, with active conductances listed in S3 Table. Synapses with similar orientation preferences were on the tips of the apical dendrites, similar to the procedure for the abstract neuron model in the main text. As before, the orientation tuning of individual synapses was achieved by scaling the AMPA and NMDA currents according to the synapse’s tuning curve. Apical segments that experienced input stimulus underwent a shift in *E*_K_^+^. The local Δ*E*_K_^+^ imposed during simulations were grouped into 3 conditions: no shift (Δ*E*_K_^+^ = 0 mV), small shifts (Δ*E*_K_^+^ = 6 mV), or large shifts (Δ*E*_K_^+^ = 18 mV), based on the [K^+^]_o_ changes found in Fig 1. To simulate a realistic somatic firing pattern, the synapses within each dendritic segment were activated randomly given by a Poisson distribution with λ = 30 ms. This stimulation protocol was repeated 3 times with a 300 ms delay between stimulation events. Each neuron setup was simulated with each of the 3 Δ*E*_K_^+^ conditions to directly assess the effect of the local *E*_K_^+^ changes.
(PDF)

S7 Fig Gain function fit. Left, multiplicative and additive gain function predictions plotted together with the original somatic firing rate for the low Δ*E*_K_^+^ condition as a function of stimulus orientation relative to target orientation. The ξ values denote the fit parameter that describes the firing rate change either as a gain coefficient or as a gain constant for the multiplicative (ξ_mul_) and additive (ξ_add_) gain transformation, respectively. Predictions were generated by either adding ξ_add_ or multiplying ξ_mul_ to the tuning curve with no *E*_K_^+^ change. Errors were assumed Gaussian and reported as ± standard deviations. Right, actual multiplicative and additive gain function fits plotted with the simulated somatic firing rate data with no *E*_K_^+^ shift (firing rate before) against low *E*_K_^+^ shift (firing rate after). The original firing rate data was plotted as ± standard deviations on each axis. To fit the data, we have only considered data with nonzero standard deviation.
(PDF)

S8 Fig Orientation tuning curve of the abstract neuron model with varying synaptic strength. Somatic tuning curve for different values of synaptic input strength while g_AMPA_ and g_NMDA_ was varied in the interval ±5% relative to the strength used in the simulations of Fig 4c. For these simulations Δ*E*_K_^+^ = 0 mV.
(PDF)

S9 Fig Discretization of the extracellular space surrounding serially connected dendrite segments. (a) The 10 μm dendritic segments with diameter R_1_ = 1 μm were concatenated to create 1 dendrite with total length 110 μm. The surface of the dendrite is unfolded to create a 2D surface where we simulate the movement of K^+^. The segments were either populated with similarly or diversely tuned synapses. Colored points indicate the placement of synapses on a 2D grid around a dendritic segment, and their color represents their target orientation, similar to Fig 1C. (b) Representation of K^+^ efflux from one synapse at different orientations. If a synapse is activated close to its preferred orientation (red line), the total amount of K^+^ efflux will be higher than a non-preferred stimulus (black line). Note that tpeak corresponds to the peak of K^+^ efflux, occurring after synaptic activation.
(PDF)

S10 Fig Dynamics of Δ*E*_K_^+^ \with varying stimulus orientation. (a) Top: Example Δ*E*_K_^+^ traces over time for a stimulus presented at the target orientation, arbitrary set at 0°. Solid lines show the evolution of Δ*E*_K_^+^ of each dendritic segment along the dendrite (different colors), with the central dendritic segment, receiving similarly tuned synapses, being the reference point (distance = 0 μm, light orange). Dotted green line shows Δ*E*_K_^+^ over time of a dendrite receiving exclusively diversely tuned input. After synaptic activation, the peak Δ*E*_K_^+^ is reached within 150–200 ms. As also shown in Fig 1, the largest shift in *E*_K_^+^ is seen for the segment receiving similarly tuned synapses. Neighboring segments, receiving diversely tuned synapses, display a gradually decreasing shift, and for dendritic segments in distance, >40 μm, the Δ*E*_K_^+^ is similar to the one expected from a dendrite receiving exclusively diversely tuned synapses (dotted green line). Middle, bottom: Δ*E*_K_^+^ as function of stimulus orientation (22.5° and 45°, respectively). For a stimulus orientation far from target orientation, the shift in the *E*_K_^+^ becomes smaller and similar to the diverse input tuning regime, as per Fig 1g and 1h. Overall, for the diversely tuned segments the smooth Δ*E*_K_^+^ is as a result well-mixed and uniform [K^+^]_o_ throughout the outer cylinder. For the similarly tuned segment, an initial drop before stabilizing in Δ*E*_K_^+^ is due to the concentration gradients with its adjacent segments, still maintaining higher Δ*E*_K_^+^ levels when compared to diversely tuned segments. (b) Same data as above, for Δ[K^+^]_o_. Δ[K^+^]_o_ lies within the interval [1:5 mM], as per Fig 1, and follows the similar trend to the Δ*E*_K_^+^. For all plots, bottom rows indicate the activation timings of individual synapses.
(PDF)

S11 Fig Diffusion in the intracellular space does not change the dynamics of Δ*E*_K_^+^. Example Δ*E*_K_^+^ traces over time for a stimulus presented at the target orientation, at different distances from the dendritic segment receiving similarly tuned synapses. Colors are as per S10 Fig Solid line: *E*_K_^+^ calculated by taking into account changes both in the intracellular and extracellular [K^+^]. Dotted line: *E*_K_^+^ calculated by changes in the [K^+^]_o_ while [K^+^]_i_ is constant. Only subtle differences are noted, with the full model laying just below the model using only [K^+^]_o_. This is to be expected, as a reduction of [K^+^]_i_ would lower *E*_K_^+^. The minimal effect of [K^+^]_i_ changes is due to the differences in the extracellular and intracellular spaces sizes, described by V_R_.
(PDF)

S12 Fig Dynamics of Δ*E*_K_^+^ with varying K_dec_. Example Δ*E*_K_^+^ traces over time for a stimulus presented at the target orientation with varying the strength of the potassium pumps (top: K_dec_ = 1.9·10^−8^ m/s, middle: K_dec_ = 2.9·10^−8^ m/s, bottom: K_dec_ = 3.9·10^−8^ m/s). Increasing the decay constant of the Na^**+**^/K^**+**^ pump lowers the Δ*E*_K_^+^ both for the similarly and diversely tuned segments and reduces the differences in Δ*E*_K_^+^ between the 2 types over time.
(PDF)

S13 Fig Dynamics of Δ*E*_K_^+^ with varying the interstimulus interval. Example Δ*E*_K_^+^ traces over time for a stimulus presented at the target orientation for interstimulus intervals 200 ms (top), 300 ms (middle), and 400 ms (bottom). For all segments, the shorter interstimulus interval (200 ms) allows for the temporal summation of the extracellular [K^+^] yet reaches similar levels of Δ*E*_K_^+^ when compared to longer intervals.
(PDF)

S14 Fig Electrochemical potential change of ions for different Δ[ion]. Left: Absolute changes in the reverse potential for K^+^ and Na^+^ for different V_R_ and Δ[ion]. Center: Ratio between the change in the potential for K^+^ and Na^+^. Right: Absolute changes in the potential for Ca^2+^ ions at different V_R_ and Δ[ion]. Note that the range of the first axis is in μm and not directly comparable with the left plot axis.
(PDF)

S15 Fig Frequency-based modulation of responses. Example Δ*E*_K_^+^ traces over time for stimuli presented at 0° (top), 22.5° (middle), and 45° (bottom) following a single stimulation event. Solid lines show the data for weight-modulated orientation tuning of synapses, i.e., based on w_syn(i,j)_(θ), with each synapse’s activation time drawn from a Poisson distribution with λ = 80 ms, as per **S10–S13 Figs**. Dotted lines show the data for frequency-modulated orientation tuning of synapses, i.e., when each synapse is activated by a Poisson train whose frequency depends on the synapse’s orientation preference (see **S2 Text** for details). Timing of inputs is plotted as raster plots (black lines: weight-modulated, gray lines: frequency-modulated). Following the stimulation event, the simulation runs for 2 s to show the return to baseline (Δ*E*_K_^+^ = 0 mV). The 2 approaches display similar dynamics, reaching same peak Δ*E*_K_^+^ responses for the similarly tuned segment within ∼200 ms. Δ*E*_K_^+^ is modulated by the stimulus orientation mainly in the segment receiving similarly tuned inputs. In contrast, the diversely tuned segments sustain a fixed Δ*E*_K_^+^ irrespective of the stimulus orientation, as per **Fig 1g**. Neighboring segments, receiving diversely tuned synapses, display a sharper decrease in the *E*_K_^+^ shift for the frequency-modulated input compared to the weight-modulated input. The slow decay to the steady state *E*_K_^+^ indicates the time window within which a second stimulation would be modulated by the elevated *E*_K_^+^. Colors of plotted lines are as **S10 Fig**.
(PDF)

S1 Table Active conductances of the point-dendrite model. (PDF)

S2 Table Active conductances of the abstract neuron model. (PDF)

S3 Table Active conductances of the neuron model for the L5 PC neuron. (PDF)

S1 Video Current-voltage attractor landscape during a dendritic spike. (a) I-V curves including intrinsic ion channels and NMDA receptors during the generation of a dendritic NMDA spike without and with *E*_K_^+^ shift. Solid and open points indicate stable and unstable fixed points, respectively, and arrows indicate system flow direction. (b) Simplified outline of the approximate V_m_ during the dendritic NMDA spike with and without *E*_K_^+^ shift, highlighting the voltage levels the V_m_ is attracted toward in a. (c) Heat maps showing the temporal evolution of the I-V curves without and with *E*_K_^+^ shift. The red line indicates the corresponding I-V curves shown in a, full and dotted white lines indicate stable and unstable fixed points, respectively, and the magenta and green lines being drawn indicate the instantaneous V_m_ for the system without and with *E*_K_^+^ shift, respectively.
(MP4)

## References

[1] Rasmussen R, O’Donnell J, Ding F, Nedergaard M. Interstitial ions: a key regulator of state-dependent neural activity? Prog Neurobiol. 2020;193:101802. doi: 10.1016/j.pneurobio.2020.101802 32413398 PMC7331944

[2] Somjen GG. Ion regulation in the brain: implications for pathophysiology. Neuroscience. 2002;8:254–267. doi: 10.1177/1073858402008003011 12061505

[3] Somjen GG. Extracellular potassium in the mammalian central nervous system. Annu Rev Physiol. 1979;41:159–177. doi: 10.1146/annurev.ph.41.030179.001111 373587

[4] Kelly JP, Van Essen DC. Cell structure and function in the visual cortex of the cat. J Physiol. 1974;238:515–547. doi: 10.1113/jphysiol.1974.sp010541 4136579 PMC1330900

[5] Singer W, Dieter LH. Extracellular potassium gradients and visual receptive fields in the cat striate cortex. Brain Res. 1975;96:378–383. doi: 10.1016/0006-8993(75)90751-9 1175021

[6] Dietz AG, Weikop P, Hauglund N, Andersen M, Petersen NC, Rose L, et al. Local extracellular K+ in cortex regulates norepinephrine levels, network state, and behavioral output. Proc Natl Acad Sci U S A. 2023;120:9. doi: 10.1073/pnas.2305071120 37774097 PMC10556678

[7] Connors B, Dray A, Fox P, Hilmy M, Somjen G. LSD’s effect on neuron populations in visual cortex gauged by transient responses of extracellular potassium evoked by optical stimuli. Neurosci Lett. 1979;13:147–150. doi: 10.1016/0304-3940(79)90032-6 530463

[8] Connors BW, Ransom BR, Kunis DM, Gutnick MJ. Activity-dependent K+ accumulation in the developing rat optic nerve. Science. 1982;216:1341–1343. doi: 10.1126/science.7079771 7079771

[9] Syková E, Rothenberg S, Krekule I. Changes of extracellular potassium concentration during spontaneous activity in the mesencephalic reticular formation of the rat. Brain Res. 1974;79:333–337. doi: 10.1016/0006-8993(74)90428-4 4420586

[10] Heinemann U, Schaible HG, Schmidt RF. Changes in extracellular potassium concentration in cat spinal cord in response to innocuous and noxious stimulation of legs with healthy and inflamed knee joints. Exp Brain Res. 1990;79:283–292. doi: 10.1007/BF00608237 2323375

[11] Brocard F, Shevtsova NA, Bouhadfane M, Tazerart S, Heinemann U, Rybak IA, et al. Activity-dependent changes in extracellular Ca2+ and K+ reveal pacemakers in the spinal locomotor-related network. Neuron. 2013;77:1047–1054. doi: 10.1016/j.neuron.2013.01.026 23522041 PMC3736142

[12] Amzica F, Massimini M, Manfridi A. Spatial buffering during slow and paroxysmal sleep oscillations in cortical networks of glial cells in vivo. J Neurosci. 2002;22:1042. doi: 10.1523/JNEUROSCI.22-03-01042.2002 11826133 PMC6758489

[13] Ding F, O’Donnell J, Xu Q, Kang N, Goldman N, Nedergaard M. Changes in the composition of brain interstitial ions control the sleep-wake cycle. Science. 2016;352:550–555. doi: 10.1126/science.aad4821 27126038 PMC5441687

[14] Rasmussen R, Nicholas E, Petersen NC, Dietz AG, Xu Q, Sun Q, et al. Cortex-wide changes in extracellular potassium ions parallel brain state transitions in awake behaving mice. Cell Rep. 2019;28:1182–1194. doi: 10.1016/j.celrep.2019.06.082 31365863 PMC6790006

[15] Heinemann U, Lux HD, Gutnick MJ. Extracellular free calcium and potassium during paroxsmal activity in the cerebral cortex of the cat. Exp Brain Res. 1977;27:237–243. doi: 10.1007/BF00235500 880984

[16] Poolos NP, Mauk MD, Kocsis JD. Activity-evoked increases in extracellular potassium modulate presynaptic excitability in the CA1 region of the hippocampus. J Neurophysiol. 1987;58:404–416. doi: 10.1152/jn.1987.58.2.404 3655875

[17] Futamachi KJ, Mutani R, Prince DA. Potassium activity in rabbit cortex. Brain Res. 1974;75:5–25. doi: 10.1016/0006-8993(74)90767-7 4842030

[18] Malenka RC, Kocsis JD, Ransom BR, Waxman SG. Modulation of parallel fiber excitability by postsynaptically mediated changes in extracellular potassium. Science. 1981;214:339–341. doi: 10.1126/science.7280695 7280695

[19] Balestrino M, Aitken PG, Somjen GG. The effects of moderate changes of extracellular K+ and Ca2+ on synaptic and neural function in the CA1 region of the hippocampal slice. Brain Res. 1986;377:229–239. doi: 10.1016/0006-8993(86)90863-2 3015348

[20] Hablitz JJ, Lundervold A. Hippocampal excitability and changes in extracellular potassium. Exp Neurol. 1981;71:410–420. doi: 10.1016/0014-4886(81)90099-6 7449908

[21] Shih PY, Savtchenko LP, Kamasawa N, Dembitskaya Y, McHugh TJ, Rusakov DA, et al. Retrograde synaptic signaling mediated by K+ efflux through postsynaptic NMDA receptors. Cell Rep. 2013;5:941–951. doi: 10.1016/j.celrep.2013.10.026 24268779

[22] Tong X, Ao Y, Faas GC, Nwaobi SE, Xu J, Haustein MD, et al. Astrocyte Kir4.1 ion channel deficits contribute to neuronal dysfunction in Huntington’s disease model mice. Nat Neurosci. 2014;17:694–703. doi: 10.1038/nn.3691 24686787 PMC4064471

[23] Utzschneider D, Kocsis J, Devor M. Mutual excitation among dorsal root ganglion neurons in the rat. Neurosci Lett. 1992;146:53–56. doi: 10.1016/0304-3940(92)90170-c 1475049

[24] Rasmussen R, Jensen MH, Heltberg ML. Chaotic dynamics mediate brain state transitions, driven by changes in extracellular ion concentrations. Cell Syst. 2017;5:591–603. doi: 10.1016/j.cels.2017.11.011 29248375

[25] Ding F, Sun Q, Long C, Rasmussen RN, Peng S, Xu Q, et al. Dysregulation of extracellular potassium distinguishes healthy ageing from neurodegeneration. Brain. 2024;147:1726–1739. doi: 10.1093/brain/awae075 38462589 PMC11068329

[26] Rice ME, Nicholson C. Glutamate- and aspartate-induced extracellular potassium and calcium shifts and their relation to those of kainate, quisqualate and N-methyl-D-aspartate in the isolated turtle cerebellum. Neuroscience. 1990;38:295–310. doi: 10.1016/0306-4522(90)90029-4 1979851

[27] Tyurikova O, Shih PY, Dembitskaya Y, Savtchenko LP, McHugh TJ, Rusakov DA, et al. K+ efflux through postsynaptic NMDA receptors suppresses local astrocytic glutamate uptake. Glia. 2022;70:961–974. doi: 10.1002/glia.24150 35084774 PMC9132042

[28] Häusser M, Mel B. Dendrites: bug or feature? Curr Opin Neurobiol. 2003;13:372–383. doi: 10.1016/s0959-4388(03)00075-8 12850223

[29] Major G, Larkum ME, Schiller J. Active properties of neocortical pyramidal neuron dendrites. Annu Rev Neurosci. 2013;36:1–24. doi: 10.1146/annurev-neuro-062111-150343 23841837

[30] Poirazi P, Papoutsi A. Illuminating dendritic function with computational models. Nat Rev Neurosci. 2020;21:303–321. doi: 10.1038/s41583-020-0301-7 32393820

[31] Stuart GJ, Spruston N. Dendritic integration: 60 years of progress. Nat Neurosci. 2015;18:1713–1721. doi: 10.1038/nn.4157 26605882

[32] Takahashi N, Kitamura K, Matsuo N, Mayford M, Kano M, Matsuki N, et al. Locally synchronized synaptic inputs. Science. 2012;335:353–356. doi: 10.1126/science.1210362 22267814

[33] Mel BW. Synaptic integration in an excitable dendritic tree. J Neurophysiol. 1993;70:1086–1101. doi: 10.1152/jn.1993.70.3.1086 8229160

[34] Poirazi P, Mel BW. Impact of active dendrites and structural plasticity on the memory capacity of neural tissue. Neuron. 2001;29:779–796. doi: 10.1016/s0896-6273(01)00252-5 11301036

[35] Poirazi P, Brannon T, Mel BW. Pyramidal neuron as two-layer neural network. Neuron. 2003;37:989–999. doi: 10.1016/s0896-6273(03)00149-1 12670427

[36] Losonczy A, Magee JC. Integrative properties of radial oblique dendrites in hippocampal CA1 pyramidal neurons. Neuron. 2006;50:291–307. doi: 10.1016/j.neuron.2006.03.016 16630839

[37] Major G, Polsky A, Denk W, Schiller J, Tank DW. Spatiotemporally graded NMDA spike/plateau potentials in basal dendrites of neocortical pyramidal neurons. J Neurophysiol. 2008;99:2584–2601. doi: 10.1152/jn.00011.2008 18337370

[38] Weber JP, Andrásfalvy BK, Polito M, Magó Á, Ujfalussy BB, Makara JK. Location-dependent synaptic plasticity rules by dendritic spine cooperativity. Nat Commun. 2016:7. doi: 10.1038/ncomms11380 27098773 PMC4844677

[39] Smith SL, Smith IT, Branco T, Häusser M. Dendritic spikes enhance stimulus selectivity in cortical neurons in vivo. Nature. 2013;503:115–120. doi: 10.1038/nature12600 24162850 PMC6319606

[40] Lavzin M, Rapoport S, Polsky A, Garion L, Schiller J. Nonlinear dendritic processing determines angular tuning of barrel cortex neurons in vivo. Nature. 2012;490:397–401. doi: 10.1038/nature11451 22940864

[41] Bittner KC, Milstein AD, Grienberger C, Romani S, Magee JC. Behavioral time scale synaptic plasticity underlies CA1 place fields. Science. 2017;357:1033–1036. doi: 10.1126/science.aan3846 28883072 PMC7289271

[42] Wilson DE, Whitney DE, Scholl B, Fitzpatrick D. Orientation selectivity and the functional clustering of synaptic inputs in primary visual cortex. Nat Neurosci. 2016;19:1003–1009. doi: 10.1038/nn.4323 27294510 PMC5240628

[43] Hoffman DA, Magee JC, Colbert CM, Johnston D. K+ channel regulation of signal propagation in dendrites of hippocampal pyramidal neurons. Nature. 1997;387:869–875. doi: 10.1038/43119 9202119

[44] Magee JC, Carruth M. Dendritic voltage-gated ion channels regulate the action potential firing mode of hippocampal CA1 pyramidal neurons. J Neurophysiol. 1999;82:1895–1901. doi: 10.1152/jn.1999.82.4.1895 10515978

[45] Iacaruso MF, Gasler IT, Hofer SB. Synaptic organization of visual space in primary visual cortex. Nature. 2017;547:449–452. doi: 10.1038/nature23019 28700575 PMC5533220

[46] Scholl B, Wilson DE, Fitzpatrick D. Local order within global disorder: synaptic architecture of visual space. Neuron. 2017;96:1127–1138. doi: 10.1016/j.neuron.2017.10.017 29103806 PMC5868972

[47] Kerlin A, Boaz M, Flickinger D, Maclennan BJ, Dean MB, Davis C, et al. Functional clustering of dendritic activity during decision-making. Elife. 2019:8. doi: 10.7554/eLife.46966 31663507 PMC6821494

[48] Winnubst J, Cheyne JE, Niculescu D, Lohmann C. Spontaneous activity drives local synaptic plasticity in vivo. Neuron. 2015;87:399–410. doi: 10.1016/j.neuron.2015.06.029 26182421

[49] Hedrick NG, Lu Z, Bushong E, Singhi S, Nguyen P, Magaña Y, et al. Learning binds new inputs into functional synaptic clusters via spinogenesis. Nat Neurosci. 2022;25:726–737. doi: 10.1038/s41593-022-01086-6 35654957 PMC12251420

[50] Kleindienst T, Winnubst J, Roth-Alpermann C, Bonhoeffer T, Lohmann C. Activity-dependent clustering of functional synaptic inputs on developing hippocampal dendrites. Neuron. 2011;72:1012–1024. doi: 10.1016/j.neuron.2011.10.015 22196336

[51] Comer AL, Jinadasa T, Sriram B, Phadke RA, Kretsge LN, Nguyen TPH, et al. Increased expression of schizophrenia-associated gene C4 leads to hypoconnectivity of prefrontal cortex and reduced social interaction. PLoS Biol. 2020:18. doi: 10.1371/journal.pbio.3000604 31935214 PMC6959572

[52] Parker EM, Kindja NL, Cheetham CEJ, Sweet RA. Sex differences in dendritic spine density and morphology in auditory and visual cortices in adolescence and adulthood. Sci Rep. 2020:10. doi: 10.1038/S41598-020-65942-W 32523006 PMC7287134

[53] Iascone DM, Li Y, Sümbül U, Doron M, Chen H, Andreu V, et al. Whole-neuron synaptic mapping reveals spatially precise excitatory/inhibitory balance limiting dendritic and somatic Spiking. Neuron. 2020;106:566–578. doi: 10.1016/j.neuron.2020.02.015 32169170 PMC7244395

[54] Jia H, Rochefort NL, Chen X, Konnerth A. Dendritic organization of sensory input to cortical neurons in vivo. Nature. 2010;464:1307–1312. doi: 10.1038/nature08947 20428163

[55] Chen T-W, Wardill TJ, Sun Y, Pulver SR, Renninger SL, Baohan A, et al. Ultrasensitive fluorescent proteins for imaging neuronal activity. Nature. 2013;499:295–300. doi: 10.1038/nature12354 23868258 PMC3777791

[56] Ellingsrud AJ, Solbrå A, Einevoll GT, Halnes G, Rognes ME. Finite element simulation of ionic electrodiffusion in cellular geometries. Front Neuroinform. 2020:14. doi: 10.3389/fninf.2020.00011 32269519 PMC7109287

[57] Chen KC, Nicholson C. Spatial buffering of potassium ions in brain extracellular space. Biophys J. 2000;78:2776–2797. doi: 10.1016/S0006-3495(00)76822-6 10827962 PMC1300867

[58] Benninger C, Kadis J, Prince DA. Extracellular calcium and potassium changes in hippocampal slices. Brain Res. 1980;187:165–182. doi: 10.1016/0006-8993(80)90502-8 7357467

[59] Aitken PG, Somjen GG. The sources of extracellular potassium accumulation in the CA1 region of hippocampal slices. Brain Res. 1986;369:163–167. doi: 10.1016/0006-8993(86)90524-x 3697739

[60] Gao L, Liu S, Wang Y, Wu Q, Gou L, Yan J. Single-neuron analysis of dendrites and axons reveals the network organization in mouse prefrontal cortex. Nat Neurosci. 2023; 26:1111–1126. doi: 10.1038/S41593-023-01339-Y 37217724

[61] Yamada R, Kuba H. Dendritic synapse geometry optimizes binaural computation in a sound localization circuit. Sci Adv. 2021:7. doi: 10.1126/SCIADV.ABH0024 34818046 PMC8612683

[62] Grafe P, Rimpel J, Reddy MM, ten Bruggencate G. Changes of intracellular sodium and potassium ion concentrations in frog spinal motoneurons induced by repetitive synaptic stimulation. Neuroscience. 1982;7:3213–3220. doi: 10.1016/0306-4522(82)90243-3 6984493

[63] Tønnesen J, Inavalli VVGK, Nägerl UV. Super-resolution imaging of the extracellular space in living brain tissue. Cell. 2018;172:1108–1121. doi: 10.1016/J.CELL.2018.02.007 29474910

[64] Pallotto M, Watkins PV, Fubara B, Singer JH, Briggman KL. Extracellular space preservation aids the connectomic analysis of neural circuits. Elife. 2015:4. doi: 10.7554/eLife.08206 26650352 PMC4764589

[65] Somjen GG, Rosenthal M, Cordingley G, LaManna J, Lothman E. Potassium, neuroglia, and oxidative metabolism in central gray matter. Fed Proc. 1976;35:1266–1271. 177318

[66] Nicholson C. Ion-selective microelectrodes and diffusion measurements as tools to explore the brain cell microenvironment. J Neurosci Methods. 1993;48:199–213. doi: 10.1016/0165-0270(93)90092-6 8412303

[67] Fröhlich F, Bazhenov M, Iragui-Madoz V, Sejnowski TJ. Potassium dynamics in the epileptic cortex: new insights on an old topic. Neuroscience. 2008;14:422–433. doi: 10.1177/1073858408317955 18997121 PMC2854295

[68] Frant MS, Ross JW. Potassium ion specific electrode with high selectivity for potassium over sodium. Science. 1970;167:987–988. doi: 10.1126/science.167.3920.987 5411177

[69] Walz W, Hertz L. Functional interactions between neurons and astrocytes. II. Potassium homeostasis at the cellular level. Prog Neurobiol. 1983;20:133–183. doi: 10.1016/0301-0082(83)90013-8 6141593

[70] Dayan P, Abbott LF. Theoretical neuroscience: computational and mathematical modeling of neural Systems. MIT Press; 2001.

[71] Hay E, Hill S, Schürmann F, Markram H, Segev I. Models of neocortical layer 5b pyramidal cells capturing a wide range of dendritic and perisomatic active properties. PLoS Comput Biol. 2011:7. doi: 10.1371/journal.pcbi.1002107 21829333 PMC3145650

[72] Park J, Papoutsi A, Ash RT, Marin MA, Poirazi P, Smirnakis SM. Contribution of apical and basal dendrites to orientation encoding in mouse V1 L2/3 pyramidal neurons. Nat Commun. 2019:10. doi: 10.1038/s41467-019-13029-0 31772192 PMC6879601

[73] Shai AS, Anastassiou CA, Larkum ME, Koch C. Physiology of layer 5 pyramidal neurons in mouse primary visual cortex: coincidence detection through bursting. PLoS Comput Biol. 2015:11. doi: 10.1371/journal.pcbi.1004090 25768881 PMC4358988

[74] Strogatz SH. Nonlinear Dynamics and Chaos. CRC Press; 2018. doi: 10.1201/9780429492563

[75] Smith JH, Rowland C, Harland B, Moslehi S, Montgomery RD, Schobert K, et al. How neurons exploit fractal geometry to optimize their network connectivity. Sci Rep. 2021:11. doi: 10.1038/s41598-021-81421-2 33504818 PMC7840685

[76] Porter R, Ghosh S, David Lange G, Smith TG. A fractal analysis of pyramidal neurons in mammalian motor cortex. Neurosci Lett. 1991;130:112–116. doi: 10.1016/0304-3940(91)90240-t 1749510

[77] Ebner C, Clopath C, Jedlicka P, Cuntz H. Unifying Long-Term Plasticity Rules for Excitatory Synapses by Modeling Dendrites of Cortical Pyramidal Neurons. Cell Rep. 2019;29:4295–4307.e6. doi: 10.1016/j.celrep.2019.11.068 31875541 PMC6941234

[78] Bloss EB, Cembrowski MS, Karsh B, Colonell J, Fetter RD, Spruston N. Single excitatory axons form clustered synapses onto CA1 pyramidal cell dendrites. Nat Neurosci. 2018;21:353–363. doi: 10.1038/s41593-018-0084-6 29459763

[79] Polack P-O, Friedman J, Golshani P. Cellular mechanisms of brain state-dependent gain modulation in visual cortex. Nat Neurosci. 2013;16:1331–1339. doi: 10.1038/nn.3464 23872595 PMC3786578

[80] Gooch HM, Bluett T, Perumal MB, Vo HD, Fletcher LN, Papacostas J, et al. High-fidelity dendritic sodium spike generation in human layer 2/3 neocortical pyramidal neurons. Cell Rep. 2022;41:111500. doi: 10.1016/j.celrep.2022.111500 36260998

[81] Stuart G, Spruston N. Determinants of voltage attenuation in neocortical pyramidal neuron dendrites. J Neurosci. 1998;18:3501–3510. doi: 10.1523/JNEUROSCI.18-10-03501.1998 9570781 PMC6793161

[82] Gasparini S, Migliore M, Magee JC. On the initiation and propagation of dendritic spikes in CA1 pyramidal neurons. J Neurosci. 2004;24:11046–11056. doi: 10.1523/JNEUROSCI.2520-04.2004 15590921 PMC6730267

[83] Polsky A, Mel BW, Schiller J. Computational subunits in thin dendrites of pyramidal cells. Nat Neurosci. 2004;7:621–627. doi: 10.1038/nn1253 15156147

[84] Johnston D, Hoffman DA, Magee JC, Poolos NP, Watanabe S, Colbert CM, et al. Dendritic potassium channels in hippocampal pyramidal neurons. J Physiol. 2000;525:75. doi: 10.1111/j.1469-7793.2000.00075.x 10811726 PMC2269937

[85] Sun W, Maffie JK, Lin L, Petralia RS, Rudy B, Hoffman DA. DPP6 establishes the A-type K+ current gradient critical for the regulation of dendritic excitability in CA1 hippocampal neurons. Neuron. 2011;71:1102–1115. doi: 10.1016/j.neuron.2011.08.008 21943606 PMC3184237

[86] Du K, Wu YW, Lindroos R, Liu Y, Rózsa B, Katona G, et al. Cell-type-specific inhibition of the dendritic plateau potential in striatal spiny projection neurons. Proc Natl Acad Sci U S A. 2017;114:E7612–E7621. doi: 10.1073/pnas.1704893114 28827326 PMC5594658

[87] Fischer L, Mojica Soto-Albors R, Tang VD, Bicknell B, Grienberger C, Francioni V, et al. Dendritic Mechanisms for In Vivo Neural Computations and Behavior. J Neurosci. 2022;42:8460–8467. doi: 10.1523/JNEUROSCI.1132-22.2022 36351832 PMC9665914

[88] Fişek M, Herrmann D, Egea-Weiss A, Cloves M, Bauer L, Lee T-Y, et al. Cortico-cortical feedback engages active dendrites in visual cortex. Nature. 2023;617: 769–776. doi: 10.1038/s41586-023-06007-6 37138089 PMC10244179

[89] Takahashi N, Oertner TG, Hegemann P, Larkum ME. Active cortical dendrites modulate perception. Science. 2016;354:1587–1590. doi: 10.1126/science.aah6066 28008068

[90] Suzuki M, Larkum ME. General anesthesia decouples cortical pyramidal neurons. Cell. 2020;180:666–676. doi: 10.1016/j.cell.2020.01.024 32084339

[91] Bast A, Guest JM, Fruengel R, Narayanan RT, de Kock CPJ, Oberlaender M. Thalamus drives active dendritic computations in cortex. bioRxiv. 2021:p. 2021.10.21.465325. doi: 10.1101/2021.10.21.465325

[92] Wu SY, Wen Y, Serre NBC, Laursen CCH, Dietz AG, Taylor BR, et al. A sensitive and specific genetically-encoded potassium ion biosensor for in vivo applications across the tree of life. PLoS Biol. 2022: 20. doi: 10.1371/journal.pbio.3001772 36067248 PMC9481166

[93] Beck C, Gong Y. A high-speed, bright, red fluorescent voltage sensor to detect neural activity. Sci Rep. 2019:9. doi: 10.1038/s41598-019-52370-8 31685893 PMC6828731

[94] Wang F, Smith NA, Xu Q, Fujita T, Baba A, Matsuda T, et al. Astrocytes modulate neural network activity by Ca2+-dependent uptake of extracellular K+. Sci Signal. 2012:5. doi: 10.1126/SCISIGNAL.2002334 22472648 PMC3515082

[95] Geiger JRP, Jonas P. Dynamic control of presynaptic Ca2+ inflow by fast-inactivating K+ channels in hippocampal mossy fiber boutons. Neuron. 2000;28:927–939. doi: 10.1016/S0896-6273(00)00164-1 11163277

[96] Sasaki T, Matsuki N, Ikegaya Y. Action-potential modulation during axonal conduction. Science. 2011;331:599–601. doi: 10.1126/science.1197598 21292979

[97] Caporale N, Dan Y. Spike timing-dependent plasticity: a hebbian learning rule. Annu Rev Neurosci. 2008;31:25–46. doi: 10.1146/annurev.neuro.31.060407.125639 18275283

[98] Feldman DE. The spike-timing dependence of plasticity. Neuron. 2012;75:556–571. doi: 10.1016/j.neuron.2012.08.001 22920249 PMC3431193

[99] Hibino H, Inanobe A, Furutani K, Murakami S, Findlay I, Kurachi Y. Inwardly rectifying potassium channels: Their structure, function, and physiological roles. Physiol Rev. 2010;90:291–366. doi: 10.1152/physrev.00021.2009 20086079

[100] Abrahamyan A, Eldstrom J, Sahakyan H, Karagulyan N, Mkrtchyan L, Karapetyan T, et al. Mechanism of external K+ sensitivity of KCNQ1 channels. J Gen Physiol. 2023:155. doi: 10.1085/jgp.202213205 36809486 PMC9960071

[101] Larsen AP, Steffensen AB, Grunnet M, Olesen SP. Extracellular potassium inhibits Kv7.1 potassium channels by stabilizing an inactivated state. Biophys J. 2011;101:818–827. doi: 10.1016/j.bpj.2011.06.034 21843472 PMC3175082

[102] d’Aquin S, Szonyi A, Mahn M, Krabbe S, Gründemann J, Lüthi A. Compartmentalized dendritic plasticity during associative learning. Science. 2022;376:eabf7052. doi: 10.1126/science.abf7052 35420958

[103] Chiu CQ, Lur G, Morse TM, Carnevale NT, Ellis-Davies GCR, Higley MJ. Compartmentalization of GABAergic inhibition by dendritic spines. Science. 2013;340:759–762. doi: 10.1126/science.1234274 23661763 PMC3752161

[104] Tønnesen J, Hrabĕtová S, Soria FN. Local diffusion in the extracellular space of the brain. Neurobiol Dis. 2023:177. doi: 10.1016/j.nbd.2022.105981 36581229

[105] Gilman JP, Medalla M, Luebke JI. Area-specific features of pyramidal neurons-a comparative study in mouse and rhesus monkey. Cereb Cortex. 2017;27:2078–2094. doi: 10.1093/cercor/bhw062 26965903 PMC6059164

[106] Benavides-Piccione R, Regalado-Reyes M, Fernaud-Espinosa I, Kastanauskaite A, Tapia-González S, León-Espinosa G, et al. Differential Structure of Hippocampal CA1 Pyramidal Neurons in the Human and Mouse. Cereb Cortex. 2020;30:730–752. doi: 10.1093/cercor/bhz122 31268532

[107] Colbourn R, Hrabe J, Nicholson C, Perkins M, Goodman JH, Hrabetova S. Rapid volume pulsation of the extracellular space coincides with epileptiform activity in mice and depends on the NBCe1 transporter. J Physiol. 2021;599:3195–3220. doi: 10.1113/JP281544 33942325 PMC9319884

[108] Petousakis KE, Park J, Papoutsi A, Smirnakis S, Poirazi P. Modeling apical and basal tree contribution to orientation selectivity in a mouse primary visual cortex layer 2/3 pyramidal cell. Elife. 2023;12:1–28. doi: 10.7554/eLife.91627 38054403 PMC10754496

[109] Halnes G, Mäki-Marttunen T, Keller D, Pettersen KH, Andreassen OA, Einevoll GT. Effect of ionic diffusion on extracellular potentials in neural tissue. PLoS Comput Biol. 2016:12. doi: 10.1371/journal.pcbi.1005193 27820827 PMC5098741

[110] Halnes G, Østby I, Pettersen KH, Omholt SW, Einevoll GT. Electrodiffusive model for astrocytic and neuronal ion concentration dynamics. PLoS Comput Biol. 2013:9. doi: 10.1371/journal.pcbi.1003386 24367247 PMC3868551

[111] Bialek W, Setayeshgar S. Physical limits to biochemical signaling. Proc Natl Acad Sci U S A. 2005;102:10040–10045. doi: 10.1073/pnas.0504321102 16006514 PMC1177398

[112] Sneppen K. Models of Life: dynamics and regulation in biological systems. 1st ed. Cambridge: Cambridge University Press; 2014.

[113] Contreras SA, Schleimer JH, Gulledge AT, Schreiber S. Activity-mediated accumulation of potassium induces a switch in firing pattern and neuronal excitability type. PLoS Comput Biol. 2021;17:1–25. doi: 10.1371/journal.pcbi.1008510 34043638 PMC8205125

[114] Ujfalussy BB, Makara JK. Impact of functional synapse clusters on neuronal response selectivity. Nat Commun. 2020:11. doi: 10.1038/s41467-020-15147-6 32179739 PMC7075899

