# Supplementary material for: Local changes in potassium ions regulate input integration in active dendrites
Source: PLoS Biol. 2024 Dec 4;22(12):e3002935. doi: 10.1371/journal.pbio.3002935 (PMC11649091; doi:10.1371/journal.pbio.3002935)
Supplement: S1 Text — (PDF) [file pbio.3002935.s001.pdf]

S1 Text: Biophysical model setup

Below we describe the biophysical models used to create ion channel dynamics of the point-dendrite model. The models used for the neuron model in NEURON were very similar but with some slight differences. For more information we refer to the files found on Git: github.com/malthenielsen/potassium_hotspots or Zenodo 10.5281/zenodo.14054295..

To mimic the physiological intra- and extracellular ionic environment we used the following concentrations:

- $[K^+]_i = 140\ mM \qquad [K^+]_o = 4\ mM \qquad (\Delta[K^+]_o \in [0.25 : 5\ mM])$
- $[Na^+]_i = 7\ mM \qquad [Na^+]_o = 140\ mM$
- $[Ca^{2+}]_i = 0.0001\ mM \qquad [Ca^{2+}]_o = 1.5\ mM$ (See $[Ca^{2+}]_i$ time dynamics below)

Intrinsic ion channels

Except for the HCN channels, intrinsic ion channels conduct a single ion species (Na^+, K^+, or Ca^{2+}), and the reversal potential for that ion species is given by:

$$E_{ion} = \frac{RT}{z_{ion}F} \ln\left(\frac{[ion]_o}{[ion]_i}\right)$$

Here $R = 8.1415 \frac{J}{mol;\ K}$ is the gas constant, $T = 311K$ is the temperature in kelvin, $F = 96485.3321 \frac{s\ A}{mol}$ is the Faraday constant, and z_{ion} is the ion valence.

Some models also includes the Q_{10} temperature-dependent coefficient denoted as T_Q:

$$T_Q = 2.3 \frac{T - 23}{10}$$

- **Leak channel (Leak)**:

$$I_{Leak} = g_{Leak}(V_m - E_{L^+}) \tag{1}$$

- **Voltage-gated Na$^+$ channel (Na$_\mathbf{V}$)**:

$$m_\tau = \frac{m_\alpha + m_\beta}{T_Q} \qquad m_\infty = \frac{m_\alpha}{m_\alpha + m_\beta} \qquad \begin{cases} m_\alpha & = \frac{0.182(V_m+35)}{1-\exp -(V_m+35)/9.8} \\ m_\beta & = \frac{0.14(-V_m-35)}{1-\exp -(-V_m-35)/9.8} \end{cases}$$

$$h_\infty = \frac{1}{\frac{1+\exp V_m+65}{T_Q}} \qquad h_\tau = \frac{h_\alpha + h_\beta}{T_Q} \qquad \begin{cases} h_\alpha & = \frac{0.024(V_m-50)}{1-\exp -(V_m-50/5} \\ h_\beta & = \frac{0.0091(-V_m+75)}{1-\exp -(-V_m+75)/5} \end{cases}$$

$$\frac{dm}{dt} = \frac{m_\infty - m}{m_\tau} \qquad \frac{dh}{dt} = \frac{h_\infty - h}{h_\tau}$$

$$I_{Na_V} = T_Q g_{Na} m^3 h((V + 25) - E_{Na^+})$$

- **Voltage-gated K$^+$ channel (K$_\mathbf{V}$)**:

$$n_\tau = \frac{n_\alpha + n_\beta}{T_Q} \qquad n_\infty = \frac{n_\alpha}{n_\alpha + n_\beta} \qquad \begin{cases} n_\alpha & = 0.02 \frac{V_m-25}{1-\exp \frac{-V_m-25}{9}} \\ n_\beta & = -0.006 \frac{V_m-25}{1-\exp \frac{V_m-25}{9}} \end{cases}$$

$$\frac{dn}{dt} = \frac{n_\infty - n}{n_\tau}$$

$$I_{K_V^+} = T_Q g_{K^+} n(V_m - E_{K^+})$$

- **M-type K^+ channel (K_M):**

$$n_\tau = \frac{n_\alpha + n_\beta}{T_Q} \qquad n_\infty = \frac{n_\alpha}{n_\alpha + n_\beta} \qquad \begin{cases} n_\alpha & = 0.001\frac{(V_m+30)}{1-\exp\frac{-V_m+30}{9}} \\ n_\beta & = -0.001\frac{(V_m+30)}{1-\exp\frac{-V_m+30}{9}} \end{cases}$$

$$\frac{dn}{dt} = \frac{n_\infty - n}{n_\tau}$$

$$I_{K_m^+} = T_Q g_{K^+} n(V_m - E_{K^+})$$

- **A-type K^+ channel (K_A):**

$$\zeta = \frac{-1.5}{1 + \frac{\exp V_m + 40}{5}}$$

$$n_\tau = \frac{n_\beta}{0.05T_Q(1+n_\alpha)} \qquad n_\infty = \frac{1}{1+n_\alpha} \qquad \begin{cases} n_\alpha & = \exp\frac{96.48\zeta(V_m-11)}{8.13(273+38)} \\ n_\beta & = \exp\frac{53.03\zeta(V_m-11)}{8.13(273+38)} \end{cases}$$

$$l_\tau = .26(V_m - 50) \qquad l_\infty = \frac{1}{1+l_\alpha} \qquad \begin{cases} l_\alpha & = \exp\frac{96.48\zeta(V_m-11)}{8.13(273+38)} \end{cases}$$

$$\frac{dn}{dt} = \frac{n_\infty - n}{n_\tau} \qquad \frac{dl}{dt} = \frac{l_\infty - l}{l_\tau}$$

$$I_{K_A^+} = g_{K_A^+} nl(V_m - E_{K^+})$$

- **Ca^{2+}-gated K^+ channel (K_{Ca}):**

$$n_\tau = \frac{n_\alpha + n_\beta}{T_Q} \qquad n_\infty = \frac{n_\alpha}{n_\alpha + n_\beta} \qquad \begin{cases} n_\alpha & = 0.01[Ca^{2+}]_i \\ n_\beta & = 0.02 \end{cases}$$

$$\frac{dn}{dt} = \frac{n_\infty - n}{n_\tau}$$

$$I_{K_{Ca}^+} = T_Q g_{K_{Ca}^+} n(V_m - E_{K^+})$$

- **Voltage-gated Ca^{2+} channel (Ca_V):**

$$m_\tau = \frac{1}{m_\alpha + m_\beta} \qquad m_\infty = \frac{m_\alpha}{m_\alpha + m_\beta} \qquad \begin{cases} m_\alpha & = 0.055\frac{-27-V_m}{\exp\frac{-27-V_m}{3.8}-1} \\ m_\beta & = .94\exp\frac{-75-V_m}{17} \end{cases}$$

$$h_\tau = \frac{1}{h_\alpha + h_\beta} \qquad h_\infty = \frac{h_\alpha}{h_\alpha + h_\beta} \qquad \begin{cases} h_\alpha & = 0.000457\exp\frac{-13-V_m}{50} \\ h_\beta & = \frac{0.0065}{\exp\frac{-V_m-15}{28}+1} \end{cases}$$

$$\frac{dm}{dt} = \frac{m_\infty - m}{m_\tau} \qquad \frac{dh}{dt} = \frac{h_\infty - h}{h_\tau}$$

$$I_{Ca_V} = g_{Ca_V} m^2 h(V_m - E_{Ca^{2+}})$$

The $[Ca^{2+}]_i$ and pumping was modeled as:

$$[\ddot{Ca}]_i = -20(A \cdot I_{Ca_V} + I_{NMDA}) - \frac{[Ca^{2+}]_i}{121.4} + \mathcal{N}(0, 3\cdot 10^{-7})$$

Here A is the surface area of the dendrite and $I_{channel}$ is the current passing at a given time. $\mathcal{N}(\mu, \sigma)$ is a random number from a normal distribution with mean μ and standard deviation σ.

- **Hyperpolarization-activated cyclic nucleotide-gated channels (HCN):**

This is the only intrinsic ion channel with mixed ion conductance, that is, both Na^+ and K^+ move through it. We can calculate the combined reversal potential as:

$$E_{HCN} = \frac{RT}{z_{ion}} \ln\left(\frac{p_{Na}[Na^+]_i + p_K[K^+]_i}{p_{Na}[Na^+]_o + p_K[K^+]_o}\right)$$

Here we assume that $p_{Na} = p_K$

$$m_\tau = \frac{1}{m_\alpha + m_\beta} \qquad m_\infty = \frac{m_\alpha}{m_\alpha + m_\beta} \qquad \begin{cases} m_\alpha & = \frac{0.00643(V_m+154.9)}{\exp\frac{V_m+154.9}{11.9} - 1} \\ m_\beta & = 0.193 \exp\frac{V_m}{33.1} \end{cases}$$

$$\frac{dm}{dt} = \frac{m_\infty - m}{m_\tau}$$

$$I_{HCN} = g_{HCN} m (V_m - E_{HCN})$$

Synaptic Receptors

As mentioned in the main text, only changes in K^+ are considered, leaving the rest of the ions unaccounted for. This hinders an update for the reversal potential for multi-ionic channels, like NMDA and AMPA, and we therefore kept them constant. For completeness, we artificially varied the reversal potentials, but it had negligible effects on the overall results, justifying our choice.

- **AMPA receptor**:

$$\frac{dA}{dt} = \frac{-A}{\tau_1} \quad \tau_1 = 0.5ms \quad A_{init} = 1.17 \qquad \frac{dB}{dt} = \frac{-B}{\tau_2} \quad \tau_2 = 1.5ms \quad B_{init} = 1.17$$

$$I_{AMPA} = \bar{g}_{AMPA}(B - A)(V_m - E_{AMPA}) \quad \begin{cases} \bar{g}_{AMPA_{point\ dendrite}} & = 4.25 \cdot 10^{-8}\ S \\ \bar{g}_{AMPA_{neuron}} & = 5 \cdot 10^{-8}\ S \\ E_{AMPA} & = 0mV \end{cases} \tag{2}$$

- **NMDA receptor**:

$$Mg_{block}(V_m) = \frac{1}{1 + [Mg^{2+}]_o / 3.57 exp(-0.1 V_m)}$$

$$\frac{dA}{dt} = \frac{-A}{\tau_1} \quad \tau_1 = 4ms \quad A_{init} = 2.6 \qquad \frac{dB}{dt} = \frac{-B}{\tau_2} \quad \tau_2 = 42ms \quad B_{init} = 2.6$$

$$I_{NMDA} = \bar{g}_{NMDA}(B - A) Mg_{block}(V_m)(V_m - E_{NMDA}) \quad \begin{cases} \bar{g}_{NMDA_{Point\ dendrite}} & = 2.45 \cdot 10^{-7}\ S \\ \bar{g}_{NMDA_{neuron}} & = 2.9 \cdot 10^{-7}\ S \\ [Mg^{2+}]_o & = 2mM \\ E_{NMDA} & = 0mV \end{cases}$$

- $GABA_A$ **receptor**:

$$\frac{dA}{dt} = \frac{-A}{\tau_1} \quad \tau_1 = 1ms \quad A_{init} = 1.17 \qquad \frac{dB}{dt} = \frac{-B}{\tau_2} \quad \tau_2 = 4ms \quad B_{init} = 1.17$$

$$I_{GABA_A} = \bar{g}_{GABA_A}(B - A)(V_m - E_{GABA_A}) \quad \begin{cases} \bar{g}_{GABA_A} & = 2.5 \cdot 10^{-8} \\ E_{GABA_A} & = -80mV \end{cases} \tag{3}$$

- **Stimulation protocol of synaptic receptors.** Synaptic receptors simulate the activation of a single synapse. Thus for the stimulation of e.g. 10 synapses, we would simulate 10 synaptic

models, each activated with its own synaptic activation timing. Before the activation time, synaptic conductances and rate parameters were set to 0. Upon a presynaptic spike (synaptic activation time), the synaptic conductance is set to $g_{receptor}$ and the rate parameters to their initial values, as described above for each receptor type. Following, the ODE of the temporal dynamics was numerically simulated for each of the receptors using an Euler method.
