# Supplementary material for: Local changes in potassium ions regulate input integration in active dendrites
Source: PLoS Biol. 2024 Dec 4;22(12):e3002935. doi: 10.1371/journal.pbio.3002935 (PMC11649091; doi:10.1371/journal.pbio.3002935)
Supplement: S2 Text — (PDF) [file pbio.3002935.s002.pdf]

S2 Text: Spatial and temporal properties of K^+ diffusion

In this section, we evaluate the spatial and temporal diffusion properties of potassium ions. We implemented a simple representation of the extracellular space and we used it to simulate the dynamics of $[K^+]_o$ changes and ΔE_{K^+} in dendritic segments receiving similarly and diversely tuned synapses. Steady-state $[K^+]_o$ and $[K^+]_i$ values are as per S1 Text. We further simulated different conditions and investigated how they affect ΔE_{K^+} dynamics.

Specifically, keeping the cylindrical definition of the extracellular space, we defined a longer dendritic branch composed of 11 individual 10 μm segments serially connected (total length: 110 μm). We assumed that the middle segment is populated with similarly-tuned inputs and the remaining segments are populated with diversely-tuned inputs. As the width of the extracellular space is much smaller than the diameter and length of the dendritic segment, we assumed that the K^+ diffusion in this direction is negligible. Thus the concentration gradient occurs in a 2D plane spanned by the surface around the dendritic segment and the length of the dendrite (**S9a Fig**). We chose to represent only the flow of ions on a 2D surface, as the timescale for radial diffusion of K^+ ions is much smaller than longitudinal diffusion due to its much shorter length. Future work could investigate the diffusion properties in a finer spatial 3D scale. To populate each dendritic segment with synapses, the 2D surface was divided into grid nodes that were placed at a constant distance of $\delta x = \delta y = 0.1$ μm, giving a node area of $A_{node} = 10^{-2} \mu m^2$. Only for the calculations of K^+ extrusion by Na^+/K^+ pumps, the 3D volume of each node was used [1]. For this, we assumed that the width of the extracellular space is $0.5 \mu m$, and thus the volume of each node is $V_{node} = 5 \cdot 10^{-3}$ μm^3. For each dendritic segment, sampling of the orientation-tuned synapses was performed as before (**Fig 1b, c, S9a Fig**). The number of synapses per segment was set to 10 [2, 3, 4]. Given the lack of information regarding the fine-scale temporal profile of K^+ release to the extracellular space during synaptic activation, we assumed that it can be modeled as a Gaussian distribution with peak at t_{peak} and a spread of $\sigma_{NMDA} = 65ms$, replicating the activation time frame of the NMDA receptors [5, 6, 7, 8](**S9b Fig**). When presenting a stimulus, the activation of a synapse depended on the stimulus orientation, implemented as $w_{syn(i,j)}(\theta)$, and the local change in $[K^+]_o$ followed the equation:

$$\Delta [K^+]_{o(i,j)}(t, \theta) = c_0 w_{syn(i,j)}(\theta) \frac{1}{\sqrt{2\pi\sigma^2_{NMDA}}} exp^{\left(\frac{\left[t_{peak}(i,j) - t \right]^2}{2\sigma^2_{NMDA}} \right)} \tag{1}$$

Here i, j indicate the location of the node on the grid, and t_{peak} is the time where K^+ efflux is maximal, following synaptic activation, and c_0 is the normalizing factor to convert the Gaussian output to the experimentally reported value of 1 mM $\Delta[K^+]_o$ for the diversely tuned input regime, according to **Fig 1g, h** ($c_0 = 5000$). As in all of our results, the activation of each synapse, $w_{syn(i,j)}(\theta)$, was based on its tuning curve, given by a half-circular normal distribution with $\sigma = 11°$ [9], with $w_{syn(i,j)}(\theta) \in [0, 1]$ (**Fig 1c**). Each dendritic segment was assigned its own segment-relative random activation time drawn from a uniform distribution [0:50] ms. Following, within one segment, each synapse's activation time was drawn from a Poisson distribution with $\lambda = 80$ ms. We repeated this stimulation protocol 3 times, with the time interval between each stimulation set to 300ms. For results shown in **S15 Fig** we kept constant $w_{syn(i,j)}$ and modulated the input frequency of synapses according to its synapse's tuning curve. Synaptic activation for a stimulus presented at the target orientation was achieved by sampling a Poisson process with frequency $f(0°) = 100$ Hz in a time 200ms window and time step dt = 0.001 s. Input frequency to the other stimulus orientations was implemented as $f(\theta)$ given by multiplying $f(0°)$ by a half-circular normal distribution with $\sigma = 11°$ [9]. For these simulations, the normalizing factor was set to $c_0 = 4000$.

To implement Na^+/K^+ pumps we used the model of [1]. We assumed that Na^+/K^+ pumps are

uniformly distributed along the dendrite and that the uptake of K^+ depended on the local $[K^+]_{o,(i,j)}$.

$$j_{K^+{}_{(i,j)}} = \frac{dK^+{}_{pump}}{dt} = K_{dec}\Delta[K^+]_{o(i,j)} \tag{2}$$

where K_{dec} is a constant that determines the rate of exponential decay of $[K^+]_o$ towards the steady-state $[K^+]_o$, $j_{K^+{}_{(i,j)}}$ is the inward flux of K^+ ions of a grid node due to the pump , and $\Delta[K^+]_o$ is the $[K^+]_o$ change away from steady-state concentration (for values see S1 Text). We chose a range of physiological realistic values of K_{dec}, based on [1], to investigate how K_{dec} affects the dynamics $[K^+]_o$. For example, for $K_{dec} = 2.9 \cdot 10^{-8}$ $[m/s]$ and $\Delta[K^+]_o = 1$ mM we would get a net flux per area of $2.9 \cdot 10^{-8}$ $\left[\frac{mol}{m^2 s}\right]$. Using this flux, we calculated the $\Delta[K^+]_o$ associated with the grid node, and updated its $[K^+]_o$, according to:

$$\Delta[K^+]_{o(i,j)} = j_{K^+_{(i,j)}} \frac{A_{node}}{V_{node}} \cdot dt \tag{3}$$

Finally, we used Fick's law of diffusion to simulate the spatially varying concentration field $[K^+]_o(x, y)$ of the extracellular space:

$$\frac{\partial[K^+]_o}{\partial t} = \nabla^2 D^*[K^+]_o \rightarrow \partial[K^+]_o = \nabla^2 D^*[K^+]_o \partial t \tag{4}$$

To solve for $[K^+]_o$ in both space and time, we used the finite difference method where we evaluated the concentration on each segment and time step [10, 11]. We assumed von Neuman boundary conditions in the two ends of the dendrite, that is $\frac{\partial[K^+]_o}{\partial x} = 0$. Across the dendrite, we implemented a periodic boundary condition, making a 2D surface into a cylinder. The time step of the simulation was $dt = 0.002$ ms. For **S11 Fig**, we also implemented K^+ flow in the intracellular space. For these simulations, we assumed that we could describe the intracellular volume adjacent to the dendritic membrane, similar to the description used for the extracellular space. For each grid node, to simulate the effect of the different intracellular and extracellular sizes, we multiplied the inward or outward K^+ flow with the volume ratio (V_R):

$$\Delta[K^+]_{i(i,j)}(t) = -c_0 w_{i,j} \frac{1}{\sqrt{2\pi\sigma^2_{NMDA}}} exp^{\left(\frac{\left[t_{peak}(i,j)-t\right]^2}{2\sigma^2_{NMDA}}\right)} V_R \tag{5}$$

Similarly, the volume ratio for the local $\Delta[K^+]_i$ from the pump of equation 6 was taken into account. To solve $[K^+]_i$ diffusion in space and time we simulated the diffusion in a spatially varying concentration field $[K^+]_i(x, y)$ following equation 7. Taking account of tortuosity ($\lambda_{in} = 3.2$ [1]), we computed the effective diffusion rate, as per equation 6 of the main text:

$$D^*_{in} = \frac{D_{K^+_{free}}}{\lambda^2_{in}} = \frac{1.96}{3.2^2} = 0.19\ (\frac{\mu m^2}{s})$$

Finally, using the Nernst equation, we calculated the ΔE_{K^+} of each segment using the segment-averaged $\Delta[K^+]_{(i,j)}$ either only of the extracellular (**S10 Fig, S12 Fig, S13 Fig, S15 Fig**) or the intracellular and extracellular (**S11 Fig**) spaces.

References

[1] Geir Halnes, Ivar Østby, Klas H. Pettersen, Stig W. Omholt, and Gaute T. Einevoll. Electrodiffusive model for astrocytic and neuronal ion concentration dynamics. PLoS computational biology, 9(12), 2013.

[2] Ashley L. Comer, Tushare Jinadasa, Balaji Sriram, Rhushikesh A. Phadke, Lisa N. Kretsge, Thanh P.H. Nguyen, Giovanna Antognetti, James P. Gilbert, Jungjoon Lee, Elena R. Newmark, Frances S. Hausmann, Sara Ann Rosenthal, Kevin Liu Kot, Yenyu Liu, William W. Yen, Borislav Dejanovic, and Alberto Cruz-Martín. Increased expression of schizophrenia-associated gene C4 leads to hypoconnectivity of prefrontal cortex and reduced social interaction. PLoS biology, 18(1), 1 2020.

[3] Emily M. Parker, Nathan L. Kindja, Claire E.J. Cheetham, and Robert A. Sweet. Sex differences in dendritic spine density and morphology in auditory and visual cortices in adolescence and adulthood. Scientific reports, 10(1), 12 2020.

[4] Daniel Maxim Iascone, Yujie Li, Uygar Sümbül, Michael Doron, Hanbo Chen, Valentine Andreu, Finola Goudy, Heike Blockus, Larry F. Abbott, Idan Segev, Hanchuan Peng, and Franck Polleux. Whole-neuron synaptic mapping reveals spatially precise excitatory/inhibitory balance limiting dendritic and somatic Spiking. Neuron, 106(4):566–578, 5 2020.

[5] M. E. Rice and C. Nicholson. Glutamate- and aspartate-induced extracellular potassium and calcium shifts and their relation to those of kainate, quisqualate and N-methyl-D-aspartate in the isolated turtle cerebellum. Neuroscience, 38(2):295–310, 1990.

[6] Pei Yu Shih, Leonid P. Savtchenko, Naomi Kamasawa, Yulia Dembitskaya, Thomas J. McHugh, Dmitri A. Rusakov, Ryuichi Shigemoto, and Alexey Semyanov. Retrograde synaptic signaling mediated by K+ efflux through postsynaptic NMDA receptors. Cell reports, 5(4):941–951, 11 2013.

[7] N. P. Poolos, M. D. Mauk, and J. D. Kocsis. Activity-evoked increases in extracellular potassium modulate presynaptic excitability in the CA1 region of the hippocampus. Journal of neurophysiology, 58(2):404–416, 1987.

[8] Olga Tyurikova, Pei Yu Shih, Yulia Dembitskaya, Leonid P. Savtchenko, Thomas J. McHugh, Dmitri A. Rusakov, and Alexey Semyanov. K+ efflux through postsynaptic NMDA receptors suppresses local astrocytic glutamate uptake. Glia, 70(5):961–974, 2022.

[9] Tsai-wen Chen, Trevor J Wardill, Yi Sun, Stefan R Pulver, Sabine L Renninger, Amy Baohan, Eric R Schreiter, Rex A Kerr, Michael B Orger, Vivek Jayaraman, Loren L Looger, Karel Svoboda, and Douglas S Kim. Nihms489004-1. 499(7458):295–300, 2013.

[10] Kenny Erleben, Melanie Ganz, and Francois Lauze. Computational Methods in Simulation, 2024.

[11] Joel H Ferziger, Milovan Perić, and Robert L Street. Finite Difference Methods. In Computational Methods for Fluid Dynamics, pages 41–79. Springer International Publishing, Cham, 2020.
