# Supplementary material for: Local changes in potassium ions regulate input integration in active dendrites
Source: PLoS Biol. 2024 Dec 4;22(12):e3002935. doi: 10.1371/journal.pbio.3002935 (PMC11649091; doi:10.1371/journal.pbio.3002935)
Supplement: S1 Fig — Example dendrite Vm traces for a similarly tuned dendritic segment as a function of stimulus orientation relative to target orientation. Individual trials are in gray and average is in teal. The colored segments in the inset (right) show the impact of the EK+ shift (comparison of the first and third responses). The first stimulation event induces a small EK+ shift (10 mV) for the target orientation. For the rest of stimulus orientations, the shift in EK+ is scaled according to Fig 1f. EK+ shifts increase dendritic spike occurrence and dendritic spike duration. (PDF) [file pbio.3002935.s004.pdf]

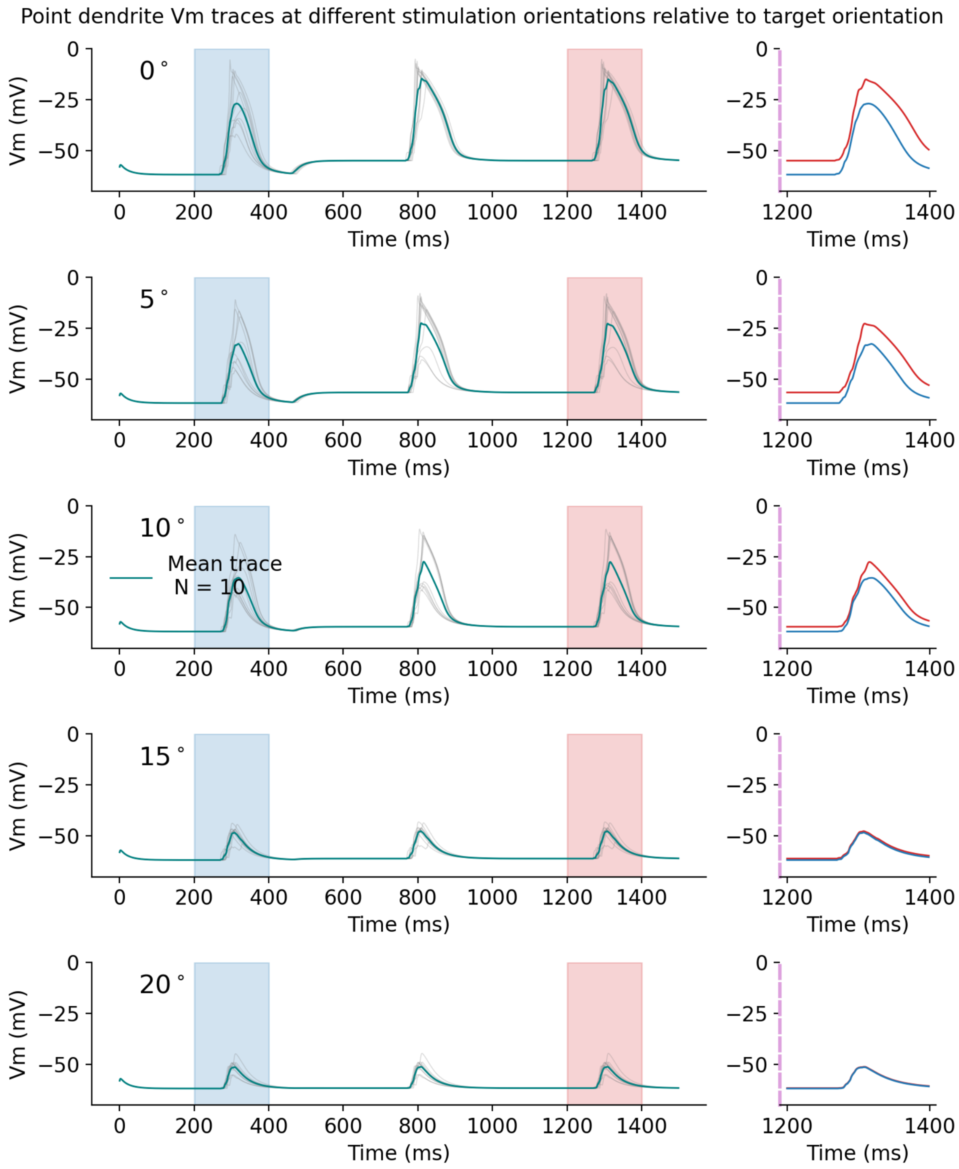

S1 Fig: Voltage response of the point dendrite with excitatory inputs.
Example dendrite V_m traces for a similarly-tuned dendritic segment as a function of stimulus orientation relative to target orientation. Individual trials are in gray and average is in teal. The colored segments in the inset (right) show the impact of the E_{K+} shift (comparison of the first and third responses). The first stimulation event induces a small E_{K+} shift (10 mV) for the target orientation. For the rest of stimulus orientations, the shift in E_{K+} is scaled according to **Fig. 1f**. E_{K+} shifts increase dendritic spike occurrence and dendritic spike duration.
