# Supplementary material for: Local changes in potassium ions regulate input integration in active dendrites
Source: PLoS Biol. 2024 Dec 4;22(12):e3002935. doi: 10.1371/journal.pbio.3002935 (PMC11649091; doi:10.1371/journal.pbio.3002935)
Supplement: S2 Fig — Top 2 plots: Example dendrite Vm traces for different orientations, as in S1 Fig, when GABAA synapses (0.2 synapses/μm density) were randomly stimulated with mean Poisson frequency 10 Hz. Following plots show example dendrite Vm traces for target orientation with increasing the inhibitory conductance and mean Poisson frequency of inhibitory activation. In each subplot with gray are voltage responses from 10 repetitions, and in teal is the mean response. The colored segments in the inset show the impact of the EK+ shift (comparison of the first and third responses). As in S1 Fig, the first stimulation event induces a small EK+ shift (10 mV) for the target orientation. High EK+ shifts increase dendritic spike occurrence and dendritic spike duration in the presence of nonspecific inhibition. High enough inhibition (bottom panel) reduces dendritic spike occurrence irrespective of the EK+ shift. (PDF) [file pbio.3002935.s005.pdf]

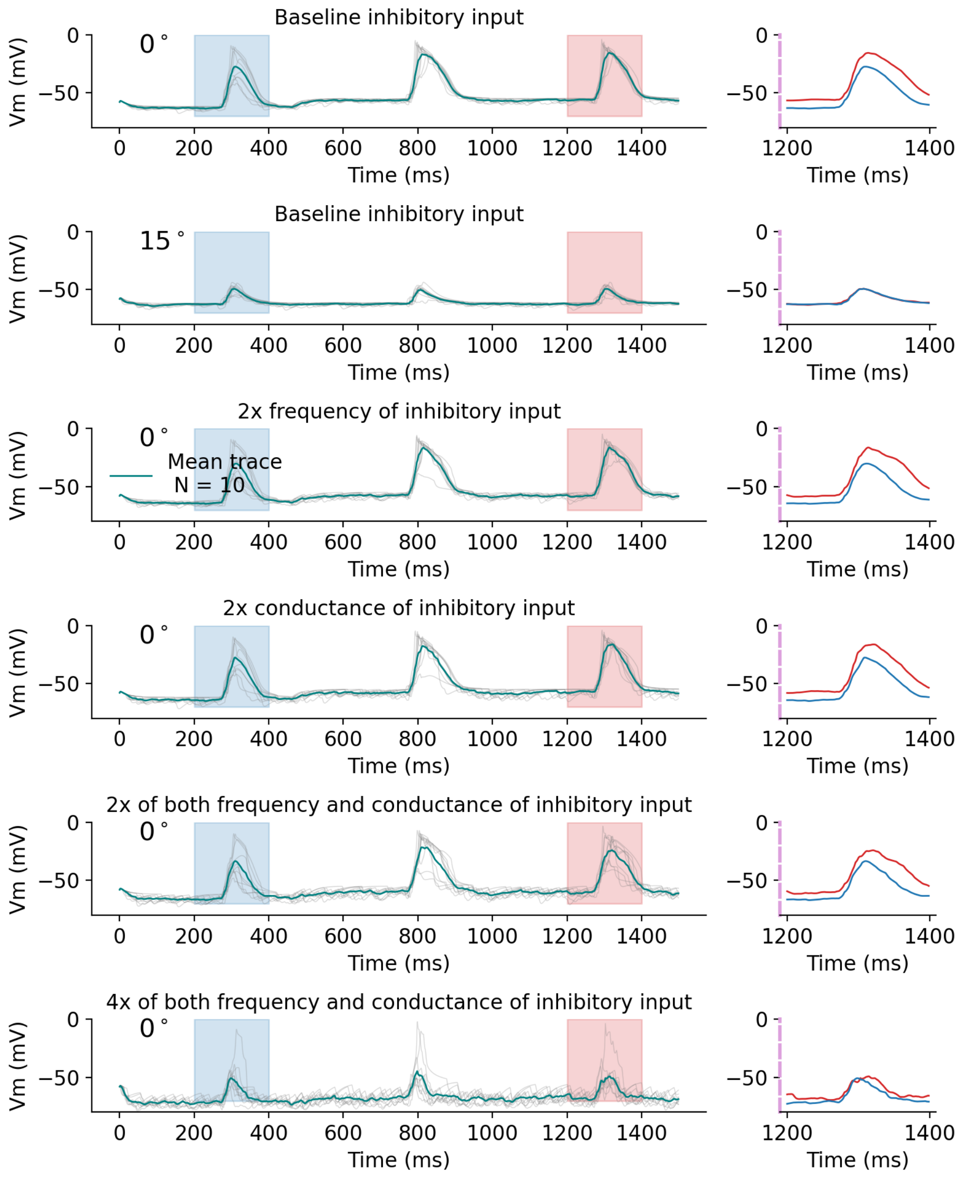

S2 Fig: Voltage response of the point dendrite with excitatory and inhibitory inputs. Top two plots: example dendrite V_m traces for different orientations, as in **S1 Fig**, when $GABA_A$ synapses (0.2 synapses/ μm density) were randomly stimulated with mean Poisson frequency 10 Hz. Following plots show example dendrite V_m traces for target orientation with increasing the inhibitory conductance and mean Poisson frequency of inhibitory activation. In each subplot with grey are voltage responses from 10 repetitions, and in teal is the mean response. The colored segments in the inset show the impact of the E_{K^+} shift (comparison of the first and third responses). As in **S1 Fig**, the first stimulation event induces a small E_{K^+} shift (10 mV) for the target orientation. High E_{K^+} shifts increase dendritic spike occurrence and dendritic spike duration in the presence of non-specific inhibition. High enough inhibition (bottom panel) reduces dendritic spike occurrence irrespective of the E_{K^+} shift.
