# Supplementary material for: Local changes in potassium ions regulate input integration in active dendrites
Source: PLoS Biol. 2024 Dec 4;22(12):e3002935. doi: 10.1371/journal.pbio.3002935 (PMC11649091; doi:10.1371/journal.pbio.3002935)
Supplement: S3 Fig — We created multiple input–output curves by varying the average synaptic activity (w), the number of synapses on the dendritic segment (N), and ΔEK+, in the point-neuron model, while having dendritic spike occurrence (Vm above –30 mV) as the output measure. This was done multiple times for each parameter combination and the resulting scatter of points in 3D space is shown here (blue circles). Following, we fitted a plane to this data (black grid), as we assumed the relation between w, N, ΔEK+ and dendritic spike generation to be linear; points on the plane denote the parameter sets which were able to generate a dendritic spike. (PDF) [file pbio.3002935.s006.pdf]

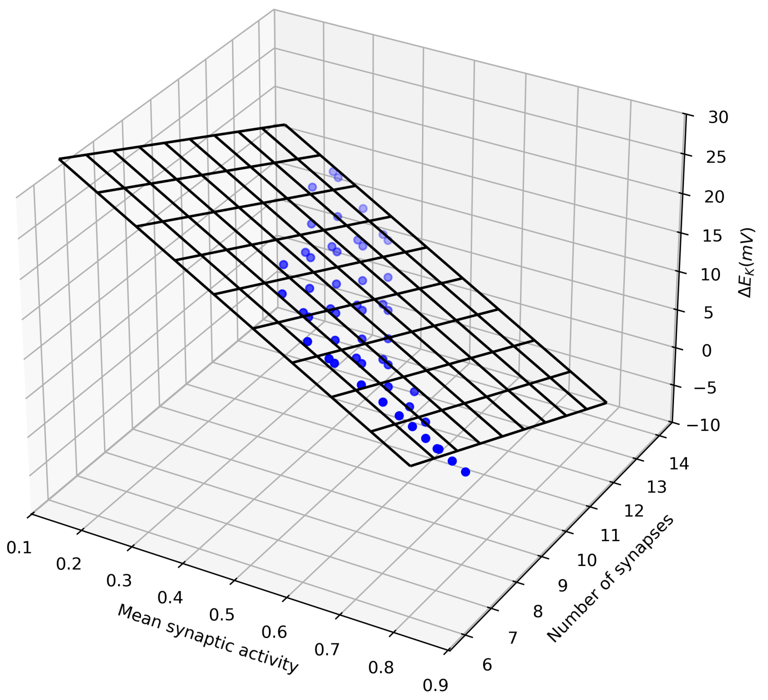


**S3 Fig: Dendritic spike emergence plane fit.**
We created multiple input-output curves by varying the average synaptic activity ($w$), the number of synapses on the dendritic segment (N), and $\Delta E_{K^+}$, in the point-neuron model, while having dendritic spike occurrence ($V_m$ above $-30$ $mV$) as the output measure. This was done multiple times for each parameter combination and the resulting scatter of points in 3D space is shown here (blue circles). Following, we fitted a plane to this data (black grid), as we assumed the relation between $w, N, \Delta E_{K^+}$ and dendritic spike generation to be linear; points on the plane denote the parameter sets which were able to generate a dendritic spike.
