# Supplementary material for: Local changes in potassium ions regulate input integration in active dendrites
Source: PLoS Biol. 2024 Dec 4;22(12):e3002935. doi: 10.1371/journal.pbio.3002935 (PMC11649091; doi:10.1371/journal.pbio.3002935)
Supplement: S4 Fig — Data similar to S3 Fig, presented here as a scatter plot for different numbers of synapses, N (colors). As suggested by the planar plot, the relationship between ΔEK+ and w is well approximated by a linear relationship. In addition, the slope of each line increases linearly with the number of synapses. (PDF) [file pbio.3002935.s007.pdf]

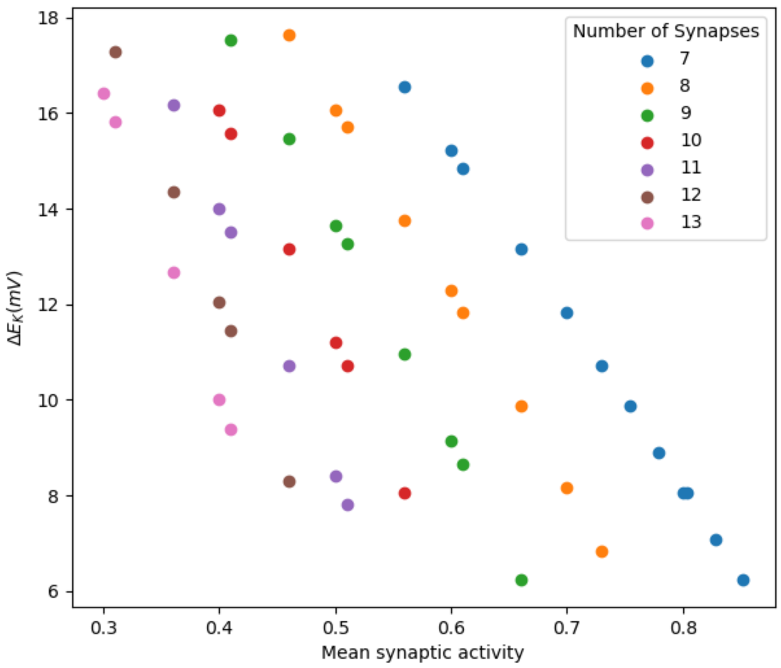

S4 Fig: Dendritic spike emergence for constant N. Data similar to **S3 Fig**, presented here as a scatter plot for different numbers of synapses, N (colors). As suggested by the planar plot, the relationship between ΔE_{K^+} and w is well approximated by a linear relationship. In addition, the slope of each line increases linearly with the number of synapses.
