# Supplementary material for: Local changes in potassium ions regulate input integration in active dendrites
Source: PLoS Biol. 2024 Dec 4;22(12):e3002935. doi: 10.1371/journal.pbio.3002935 (PMC11649091; doi:10.1371/journal.pbio.3002935)
Supplement: S5 Fig — Heat map showing peak Vm depolarization as a function of mean synaptic activity (w) and the number of synapses (N) in the point dendrite model. Dotted lines indicate the transition to dendritic spiking. Computed for ΔEK+ = 0 mV. Compare this figure to Fig 2f of main text. (PDF) [file pbio.3002935.s008.pdf]

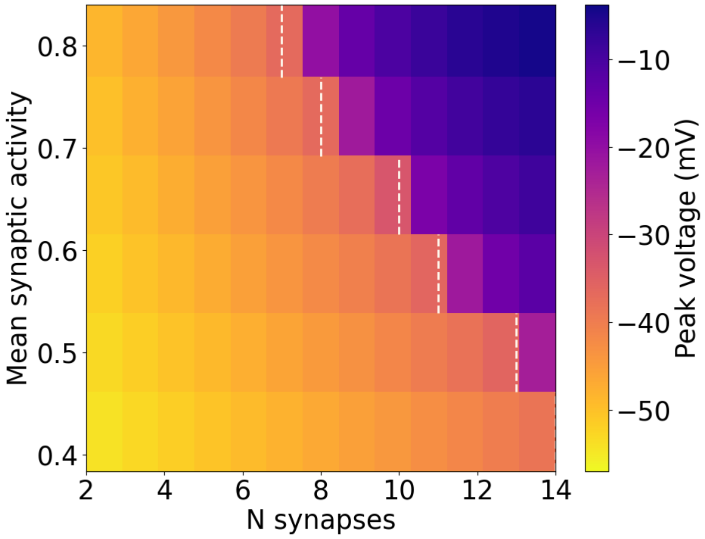

S5 Fig: Synaptic activity and number of synapses regulate active dendritic properties. Heat map showing peak V_m depolarization as a function of mean synaptic activity (w) and the number of synapses (N) in the point dendrite model. Dotted lines indicate the transition to dendritic spiking. Computed for $\Delta E_{K^+} = 0\ mV$. Compare this figure to **Fig. 2f** of main text.
