# Supplementary material for: Local changes in potassium ions regulate input integration in active dendrites
Source: PLoS Biol. 2024 Dec 4;22(12):e3002935. doi: 10.1371/journal.pbio.3002935 (PMC11649091; doi:10.1371/journal.pbio.3002935)
Supplement: S6 Fig — Somatic firing rate tuning curve for different dendritic EK+ shift magnitudes, similar to Fig 4. To further verify the results obtained with the abstract neuron of Fig 4, we simulated a neuron with detailed, reconstructed morphology. Here, we chose the widely used model of a layer 5 pyramidal neuron, obtained from [1]. The neuronal setup was kept similar to the one used for the abstract neuron, with active conductances listed in S3 Table. Synapses with similar orientation preferences were on the tips of the apical dendrites, similar to the procedure for the abstract neuron model in the main text. As before, the orientation tuning of individual synapses was achieved by scaling the AMPA and NMDA currents according to the synapse’s tuning curve. Apical segments that experienced input stimulus underwent a shift in EK+. The local ΔEK+ imposed during simulations were grouped into 3 conditions: no shift (ΔEK+ = 0 mV), small shifts (ΔEK+ = 6 mV), or large shifts (ΔEK+ = 18 mV), based on the [K+]o changes found in Fig 1. To simulate a realistic somatic firing pattern, the synapses within each dendritic segment were activated randomly given by a Poisson distribution with λ = 30 ms. This stimulation protocol was repeated 3 times with a 300 ms delay between stimulation events. Each neuron setup was simulated with each of the 3 ΔEK+ conditions to directly assess the effect of the local EK+ changes. (PDF) [file pbio.3002935.s009.pdf]

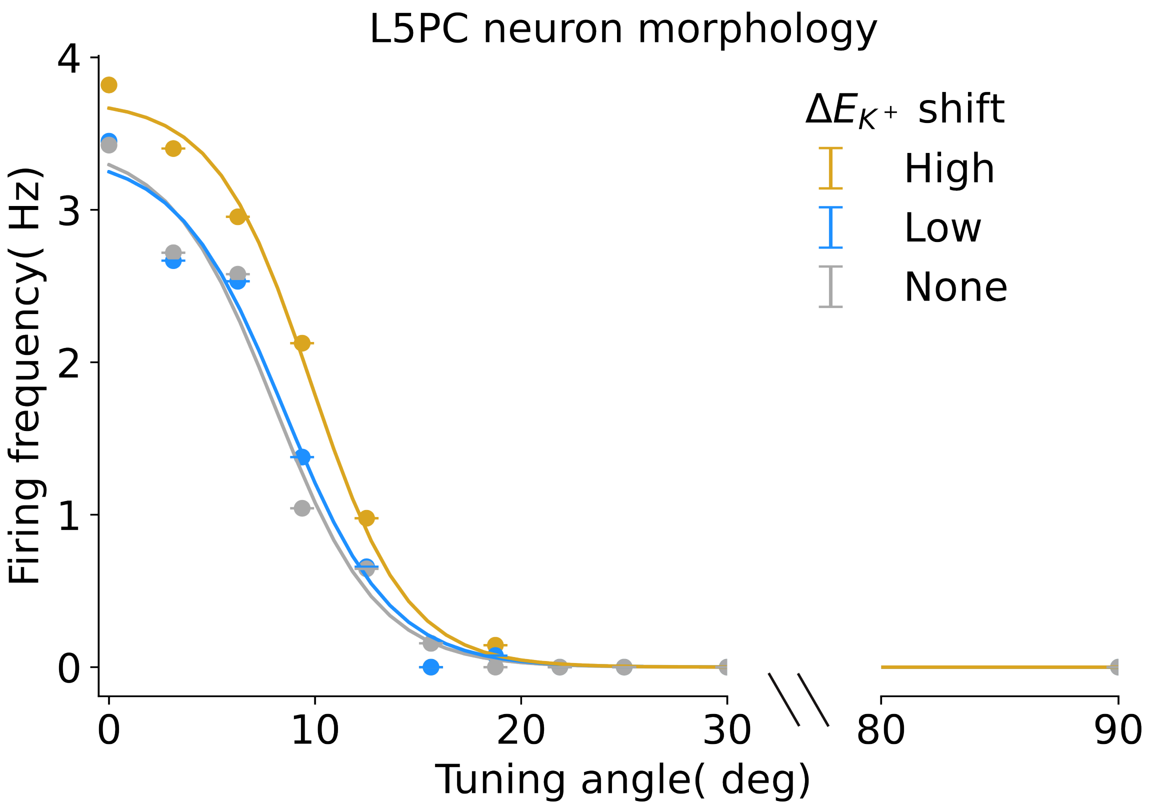

S6 Fig: Orientation tuning of a layer 5 pyramidal cortical neuron, at different ΔE_{K^+}. Somatic firing rate tuning curve for different dendritic E_{K^+} shift magnitudes, similar to **Fig 4**. To further verify the results obtained with the abstract neuron of **Fig 4**, we simulated a neuron with detailed, reconstructed morphology. Here we chose the widely used model of a layer 5 pyramidal neuron, obtained from [1]. The neuronal setup was kept similar to the one used for the abstract neuron, with active conductances listed in S3 Table. Synapses with similar orientation preferences were on the tips of the apical dendrites, similar to the procedure for the abstract neuron model in the main text. As before, the orientation tuning of individual synapses was achieved by scaling the AMPA and NMDA currents according to the synapse's tuning curve. Apical segments that experienced input stimulus underwent a shift in E_{K^+}. The local ΔE_{K^+} imposed during simulations were grouped into three conditions: no shift ($\Delta E_{K^+} = 0\ mV$), small shifts ($\Delta E_{K^+} = 6\ mV$), or large shifts ($\Delta E_{K^+} = 18\ mV$), based on the $[K^+]_o$ changes found in **Fig 1**. To simulate a realistic somatic firing pattern, the synapses within each dendritic segment were activated randomly given by a Poisson distribution with $\lambda = 30ms$. This stimulation protocol was repeated three times with a 300 ms delay between stimulation events. Each neuron setup was simulated with each of the three ΔE_{K^+} conditions to directly assess the effect of the local E_{K^+} changes.

References

[1] Etay Hay, Sean Hill, Felix Schürmann, Henry Markram, and Idan Segev. Models of neocortical layer 5b pyramidal cells capturing a wide range of dendritic and perisomatic active properties. PLoS computational biology, 7(7), 2011.
