# Supplementary material for: Local changes in potassium ions regulate input integration in active dendrites
Source: PLoS Biol. 2024 Dec 4;22(12):e3002935. doi: 10.1371/journal.pbio.3002935 (PMC11649091; doi:10.1371/journal.pbio.3002935)
Supplement: S7 Fig — Left, multiplicative and additive gain function predictions plotted together with the original somatic firing rate for the low ΔEK+ condition as a function of stimulus orientation relative to target orientation. The ξ values denote the fit parameter that describes the firing rate change either as a gain coefficient or as a gain constant for the multiplicative (ξmul) and additive (ξadd) gain transformation, respectively. Predictions were generated by either adding ξadd or multiplying ξmul to the tuning curve with no EK+ change. Errors were assumed Gaussian and reported as ± standard deviations. Right, actual multiplicative and additive gain function fits plotted with the simulated somatic firing rate data with no EK+ shift (firing rate before) against low EK+ shift (firing rate after). The original firing rate data was plotted as ± standard deviations on each axis. To fit the data, we have only considered data with nonzero standard deviation. (PDF) [file pbio.3002935.s010.pdf]

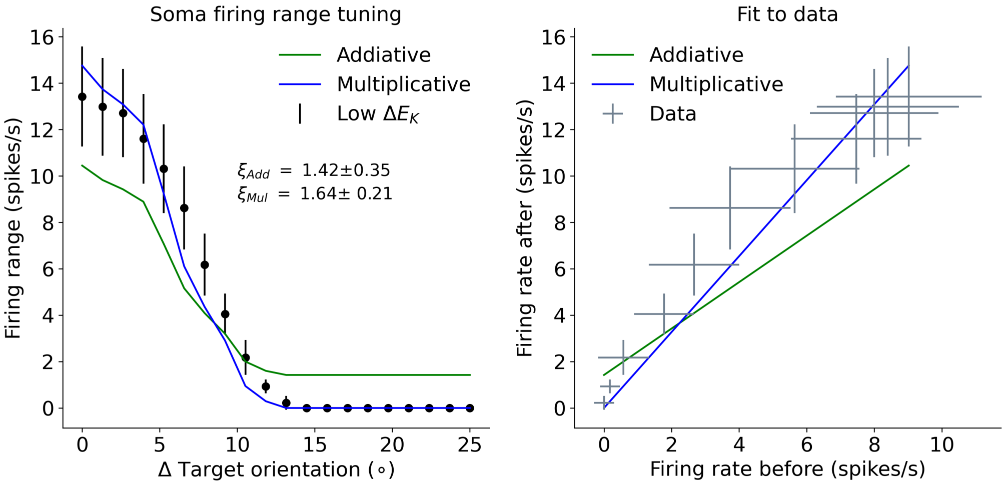

S7 Fig: Gain function fit.

Left, Multiplicative and additive gain function predictions plotted together with the original somatic firing rate for the low ΔE_{K+} condition as a function of stimulus orientation relative to target orientation. The ξ values denote the fit parameter that describes the firing rate change either as a gain coefficient or as a gain constant for the multiplicative (ξ_{mul}) and additive (ξ_{add}) gain transformation, respectively. Predictions were generated by either adding ξ_{add} or multiplying ξ_{mul} to the tuning curve with no E_{K+} change (curve not shown). Errors were assumed Gaussian and reported as ± standard deviations.

Right, Actual multiplicative and additive gain function fits plotted with the simulated somatic firing rate data with no E_{K+} shift (firing rate before) against low E_{K+} shift (firing rate after). The original firing rate data was plotted as ± standard deviations on each axis. To fit the data we have only considered data with non-zero standard deviation.
