# Supplementary material for: Local changes in potassium ions regulate input integration in active dendrites
Source: PLoS Biol. 2024 Dec 4;22(12):e3002935. doi: 10.1371/journal.pbio.3002935 (PMC11649091; doi:10.1371/journal.pbio.3002935)
Supplement: S8 Fig — Somatic tuning curve for different values of synaptic input strength while gAMPA and gNMDA was varied in the interval ±5% relative to the strength used in the simulations of Fig 4c. For these simulations ΔEK+ = 0 mV. (PDF) [file pbio.3002935.s011.pdf]

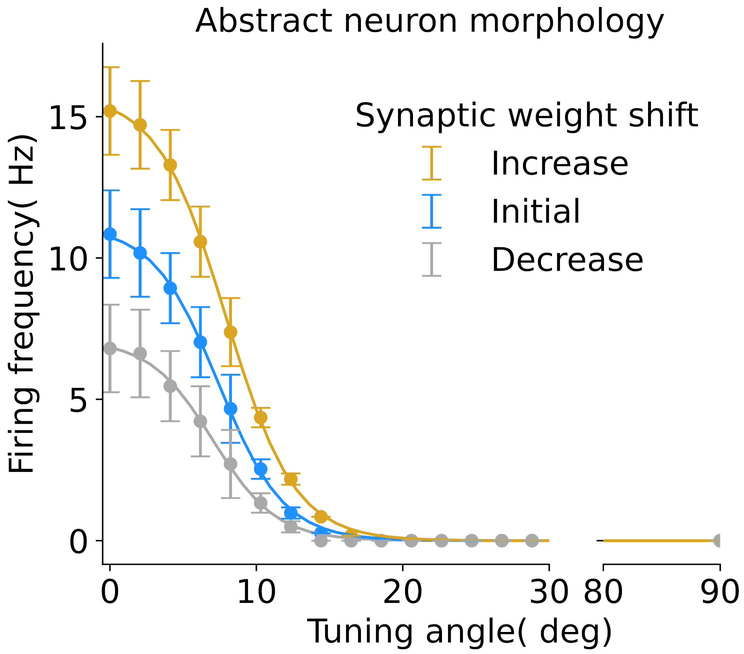

S8 Fig: Orientation tuning curve of the abstract neuron model with varying synaptic strength.

Somatic tuning curve for different values of synaptic input strength while g_{AMPA} and g_{NMDA} was varied in the interval $\pm 5\%$ relative to the strength used in the simulations of **Fig 4c**. For these simulations $\Delta E_{K^+} = 0\ mV$.
