# Supplementary material for: Local changes in potassium ions regulate input integration in active dendrites
Source: PLoS Biol. 2024 Dec 4;22(12):e3002935. doi: 10.1371/journal.pbio.3002935 (PMC11649091; doi:10.1371/journal.pbio.3002935)
Supplement: S9 Fig — (a) The 10 μm dendritic segments with diameter R1 = 1 μm were concatenated to create 1 dendrite with total length 110 μm. The surface of the dendrite is unfolded to create a 2D surface where we simulate the movement of K+. The segments were either populated with similarly or diversely tuned synapses. Colored points indicate the placement of synapses on a 2D grid around a dendritic segment, and their color represents their target orientation, similar to Fig 1C. (b) Representation of K+ efflux from one synapse at different orientations. If a synapse is activated close to its preferred orientation (red line), the total amount of K+ efflux will be higher than a non-preferred stimulus (black line). Note that tpeak corresponds to the peak of K+ efflux, occurring after synaptic activation. (PDF) [file pbio.3002935.s012.pdf]

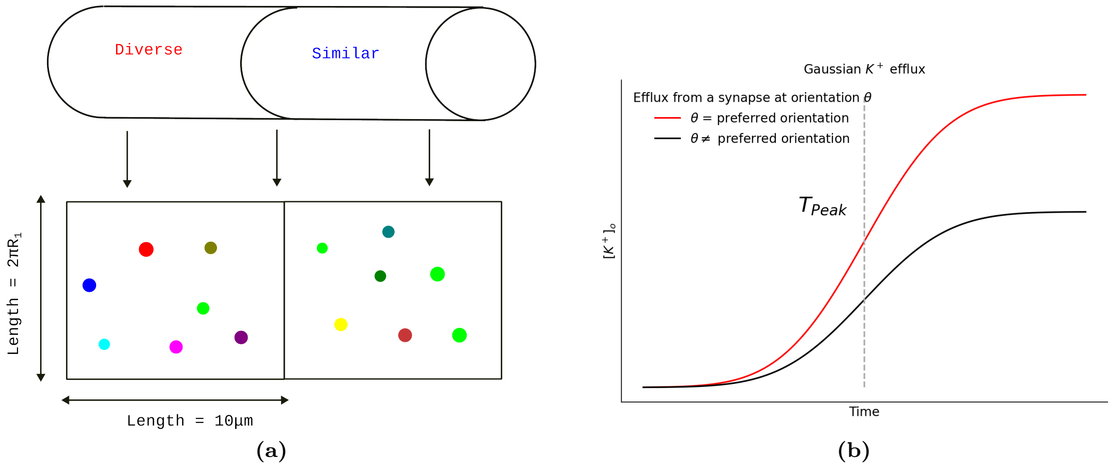

S9 Fig: Discretization of the extracellular space surrounding serially connected dendrite segments.

(a) $10\mu m$ dendritic segments with diameter $R_1 = 1\mu m$ were concatenated to create one dendrite with total length $110\mu m$. The surface of the dendrite is unfolded to create a 2D surface where we simulate the movement of K^+. The segments were either populated with similarly or diversely tuned synapses. Colored points indicate the placement of synapses on a 2D grid around a dendritic segment, and their color represents their target orientation, similar to **Fig. 1c**.

(b) Representation of K^+ efflux from one synapse at different orientations. If a synapse is activated close to its preferred orientation (red line), the total amount of K^+ efflux will be higher than a non-preferred stimulus (black line). Note that t_{peak} corresponds to the peak of K^+ efflux, occurring after synaptic activation.
