# Supplementary material for: Local changes in potassium ions regulate input integration in active dendrites
Source: PLoS Biol. 2024 Dec 4;22(12):e3002935. doi: 10.1371/journal.pbio.3002935 (PMC11649091; doi:10.1371/journal.pbio.3002935)
Supplement: S10 Fig — (a) Top: Example ΔEK+ traces over time for a stimulus presented at the target orientation, arbitrary set at 0°. Solid lines show the evolution of ΔEK+ of each dendritic segment along the dendrite (different colors), with the central dendritic segment, receiving similarly tuned synapses, being the reference point (distance = 0 μm, light orange). Dotted green line shows ΔEK+ over time of a dendrite receiving exclusively diversely tuned input. After synaptic activation, the peak ΔEK+ is reached within 150–200 ms. As also shown in Fig 1, the largest shift in EK+ is seen for the segment receiving similarly tuned synapses. Neighboring segments, receiving diversely tuned synapses, display a gradually decreasing shift, and for dendritic segments in distance, >40 μm, the ΔEK+ is similar to the one expected from a dendrite receiving exclusively diversely tuned synapses (dotted green line). Middle, bottom: ΔEK+ as function of stimulus orientation (22.5° and 45°, respectively). For a stimulus orientation far from target orientation, the shift in the EK+ becomes smaller and similar to the diverse input tuning regime, as per Fig 1g and 1h. Overall, for the diversely tuned segments the smooth ΔEK+ is as a result well-mixed and uniform [K+]o throughout the outer cylinder. For the similarly tuned segment, an initial drop before stabilizing in ΔEK+ is due to the concentration gradients with its adjacent segments, still maintaining higher ΔEK+ levels when compared to diversely tuned segments. (b) Same data as above, for Δ[K+]o. Δ[K+]o lies within the interval [1:5 mM], as per Fig 1, and follows the similar trend to the ΔEK+. For all plots, bottom rows indicate the activation timings of individual synapses. (PDF) [file pbio.3002935.s013.pdf]

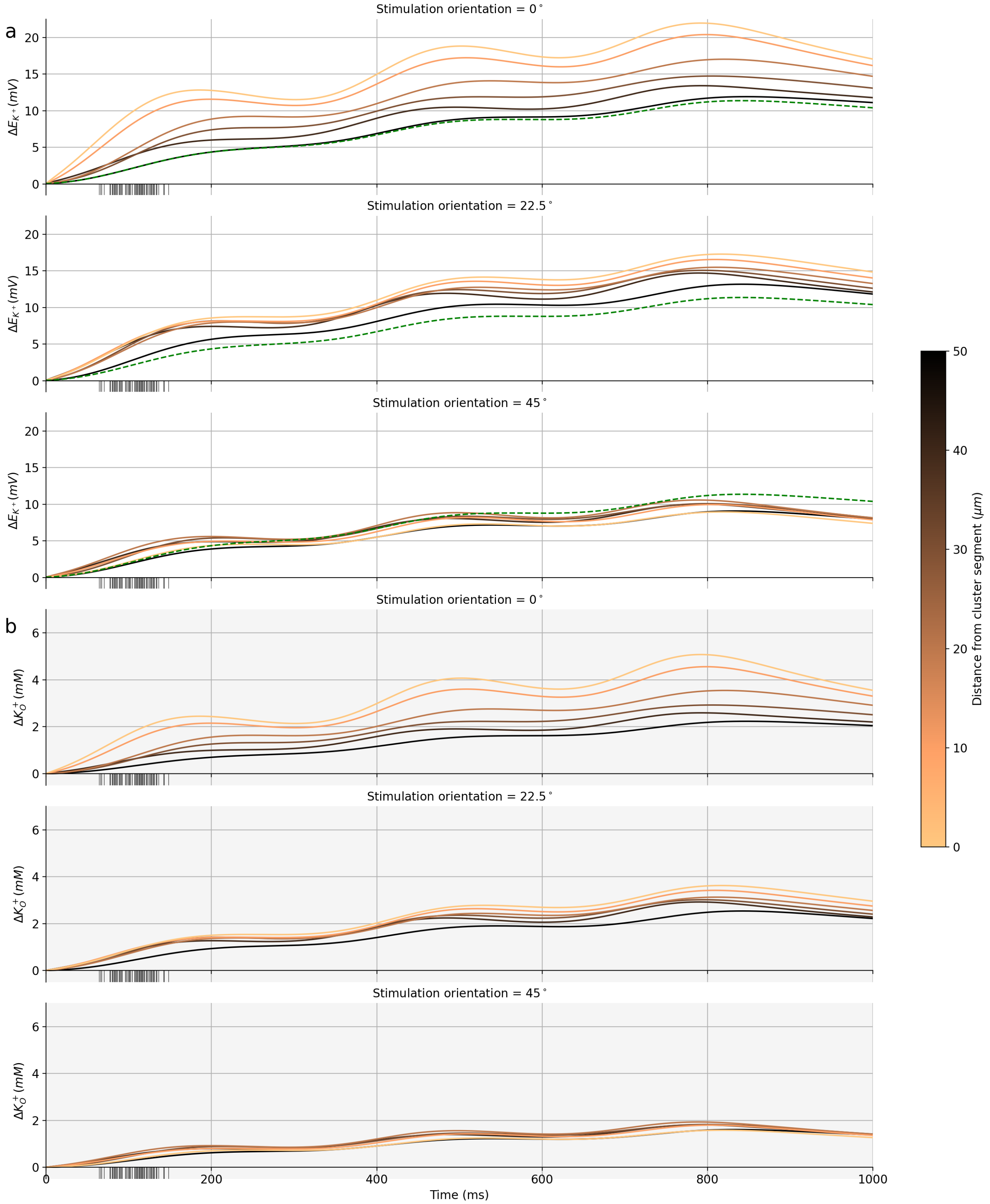

a
b
Stimulation orientation = 0°
Stimulation orientation = 22.5°
Stimulation orientation = 45°
$\Delta E_{K^+} (mV)$
$\Delta K_O^+ (mM)$
Time (ms)
Distance from cluster segment (μm)
0
200
400
600
800
1000

S10 Fig: Dynamics of ΔK_o^+ with varying stimulus orientation.

a) Top: Example ΔE_{K+} traces over time for a stimulus presented at the target orientation, arbitrary set at 0°. Solid lines show the evolution of ΔE_{K+} of each dendritic segment along the dendrite (different colors), with the central dendritic segment receiving similarly tuned synapses, being the reference point (distance= 0 µm, light orange). Dotted green line shows ΔE_{K+} over time of a dendrite receiving exclusively diversely-tuned input. After synaptic activation, the peak ΔE_{K+} is reached within 150-200 ms. As also shown in Fig 1, the largest shift in E_{K+} is seen for the segment receiving similarly tuned synapses. Neighboring segments, receiving diversely tuned synapses, display a gradually decreasing shift, and for dendritic segments in distance, $>$ 40 µm, the ΔE_{K+} is similar to the one expected from a dendrite receiving exclusively diversely-tuned synapses (dotted green line). Middle, bottom: ΔE_{K+} as function of stimulus orientation (22.5° and 45° respectively). For a stimulus orientation far from target orientation, the shift in the E_{K+} becomes smaller and similar to the diverse input tuning regime, as per **Fig 1g, 1h**. Overall, for the diversely-tuned segments the smooth ΔE_{K+} is as a result well-mixed and uniform $[K^+]_o$ throughout the outer cylinder. For the similarly-tuned segment an initial drop before stabilizing in ΔE_{K+} is due to the concentration gradients with its adjacent segments, still maintaining higher ΔE_{K+} levels when compared to diversely-tuned segments. b) Same data as above, for $\Delta[K^+]_o$. $\Delta[K^+]_o$ lies within the interval [1:5 mM], as per **Fig 1**, and follows the similar trend to the ΔE_{K+}. For all plots, bottom rows indicate the activation timings of individual synapses.
