# Supplementary material for: Local changes in potassium ions regulate input integration in active dendrites
Source: PLoS Biol. 2024 Dec 4;22(12):e3002935. doi: 10.1371/journal.pbio.3002935 (PMC11649091; doi:10.1371/journal.pbio.3002935)
Supplement: S11 Fig — Example ΔEK+ traces over time for a stimulus presented at the target orientation, at different distances from the dendritic segment receiving similarly tuned synapses. Colors are as per S10 Fig Solid line: EK+ calculated by taking into account changes both in the intracellular and extracellular [K+]. Dotted line: EK+ calculated by changes in the [K+]o while [K+]i is constant. Only subtle differences are noted, with the full model laying just below the model using only [K+]o. This is to be expected, as a reduction of [K+]i would lower EK+. The minimal effect of [K+]i changes is due to the differences in the extracellular and intracellular spaces sizes, described by VR. (PDF) [file pbio.3002935.s014.pdf]

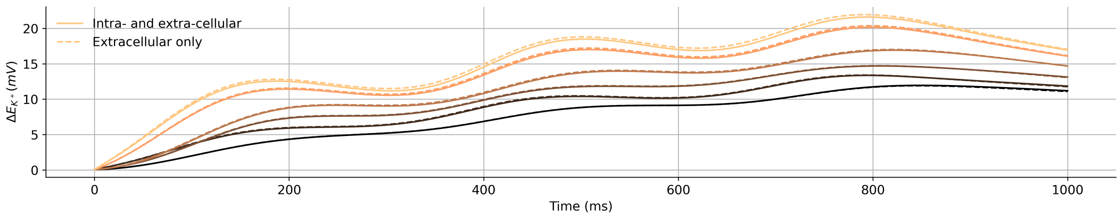

S11 Fig: Diffusion in the intracellular space does not change the dynamics of $\mathbf{\Delta E_{K^+}}$. Example ΔE_{K^+} traces over time for a stimulus presented at the target orientation, at different distances from the dendritic segment receiving similarly-tuned synapses. Colors are as per **S10 Fig**. Solid line: E_{K^+} calculated by taking into account changes both in the intracellular and extracellular $[K^+]$. Dotted line: E_{K^+} calculated by changes in the $[K^+]_o$ while $[K^+]_i$ is constant. Only subtle differences are noted, with the full model laying just below the model using only $[K^+]_o$. This is to be expected, as a reduction of $[K^+]_i$ would lower E_{K^+}. The minimal effect of $[K^+]_i$ changes is due to the differences in the extracellular and intracellular spaces sizes, described by V_R.
