# Supplementary material for: Local changes in potassium ions regulate input integration in active dendrites
Source: PLoS Biol. 2024 Dec 4;22(12):e3002935. doi: 10.1371/journal.pbio.3002935 (PMC11649091; doi:10.1371/journal.pbio.3002935)
Supplement: S12 Fig — Example ΔEK+ traces over time for a stimulus presented at the target orientation with varying the strength of the potassium pumps (top: Kdec = 1.9·10−8 m/s, middle: Kdec = 2.9·10−8 m/s, bottom: Kdec = 3.9·10−8 m/s). Increasing the decay constant of the Na+/K+ pump lowers the ΔEK+ both for the similarly and diversely tuned segments and reduces the differences in ΔEK+ between the 2 types over time. (PDF) [file pbio.3002935.s015.pdf]

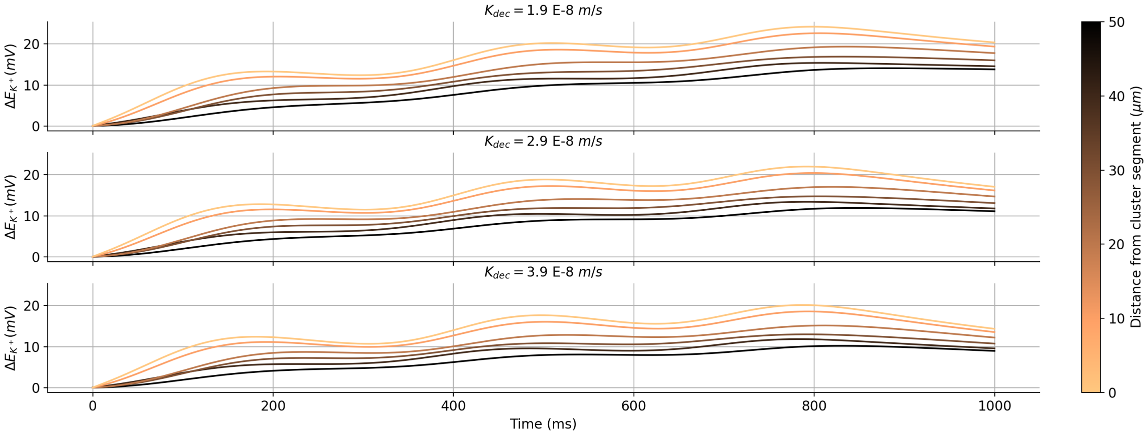

S12 Fig: Dynamics of $\mathbf{\Delta E_{K^+}}$ with varying K_{dec}.

Example ΔE_{K+} traces over time for a stimulus presented at the target orientation with varying the strength of the potassium pumps (top: $K_{dec} = 1.9 \cdot 10^{-8}\ m/s$, middle: $K_{dec} = 2.9 \cdot 10^{-8}\ m/s$, bottom: $K_{dec} = 3.9 \cdot 10^{-8}\ m/s$). Increasing the decay constant of the Na^+/K^+ pump lowers the ΔE_{K+} both for the similarly- and diversely- tuned segments and reduces the differences in ΔE_{K+} between the two types over time.
