# Supplementary material for: Local changes in potassium ions regulate input integration in active dendrites
Source: PLoS Biol. 2024 Dec 4;22(12):e3002935. doi: 10.1371/journal.pbio.3002935 (PMC11649091; doi:10.1371/journal.pbio.3002935)
Supplement: S13 Fig — Example ΔEK+ traces over time for a stimulus presented at the target orientation for interstimulus intervals 200 ms (top), 300 ms (middle), and 400 ms (bottom). For all segments, the shorter interstimulus interval (200 ms) allows for the temporal summation of the extracellular [K+] yet reaches similar levels of ΔEK+ when compared to longer intervals. (PDF) [file pbio.3002935.s016.pdf]

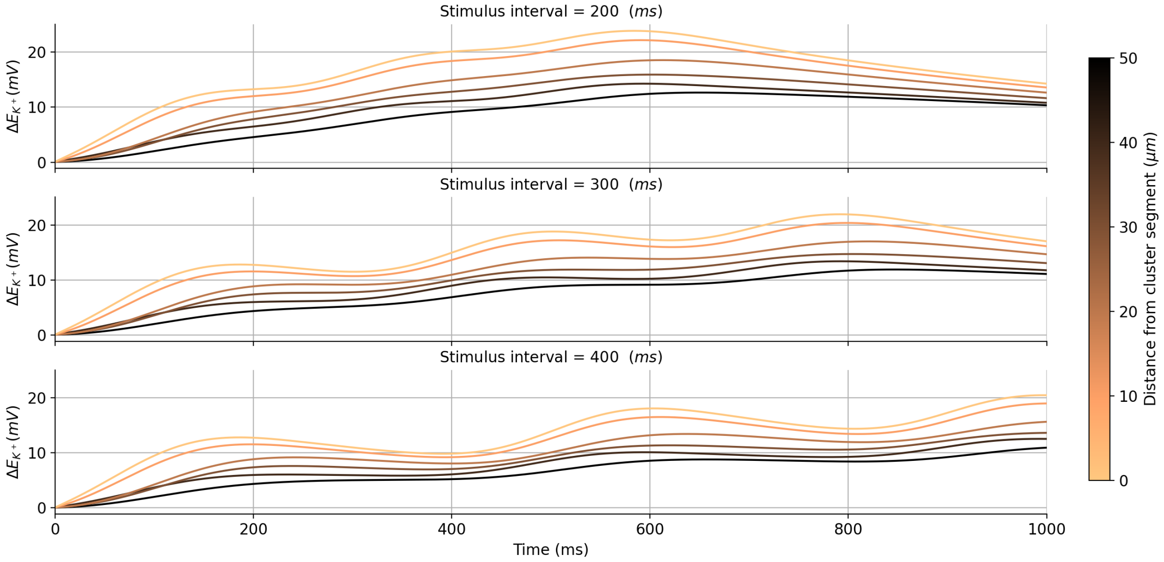

S13 Fig: Dynamics of $\mathbf{\Delta E_{K^+}}$ with varying the interstimulus interval.
Example ΔE_{K+} traces over time for a stimulus presented at the target orientation for interstimulus intervals 200 ms (top), 300 ms (middle) and 400 ms (bottom). For all segments, the shorter interstimulus interval (200ms) allows for the temporal summation of the extracellular $[K^+]$ yet reaches similar levels of ΔE_{K+} when compared to longer intervals.
