# Supplementary material for: Local changes in potassium ions regulate input integration in active dendrites
Source: PLoS Biol. 2024 Dec 4;22(12):e3002935. doi: 10.1371/journal.pbio.3002935 (PMC11649091; doi:10.1371/journal.pbio.3002935)
Supplement: S14 Fig — Left: Absolute changes in the reverse potential for K+ and Na+ for different VR and Δ[ion]. Center: Ratio between the change in the potential for K+ and Na+. Right: Absolute changes in the potential for Ca2+ ions at different VR and Δ[ion]. Note that the range of the first axis is in μm and not directly comparable with the left plot axis. (PDF) [file pbio.3002935.s017.pdf]

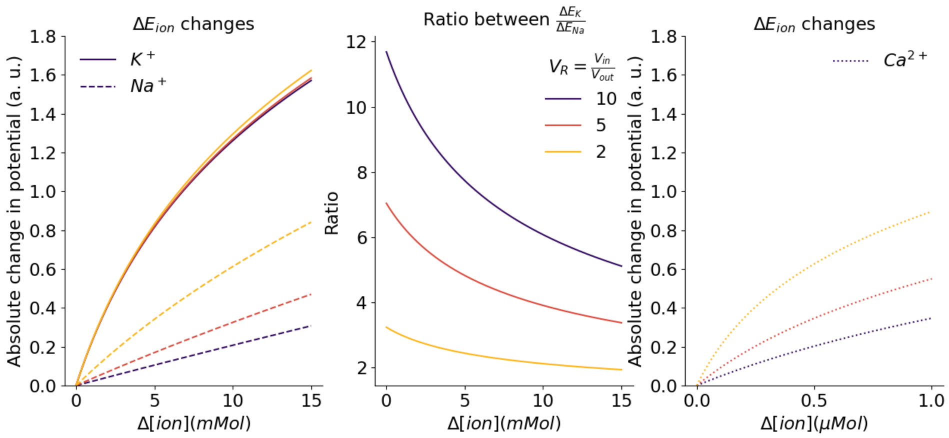

S14 Fig: Electrochemical potential change of ions for different $\Delta[ion]$.
Left: Absolute changes in the reverse potential for K^+ and Na^+ for different V_R and $\Delta[ion]$.
Center: Ratio between the change in the potential for K^+ and Na^+.
Right: Absolute changes in the potential for Ca^{2+} ions at different V_R and $\Delta[ion]$. Note that the range of the first axis is in μM and not directly comparable with the left plot axis.
