# Supplementary material for: Local changes in potassium ions regulate input integration in active dendrites
Source: PLoS Biol. 2024 Dec 4;22(12):e3002935. doi: 10.1371/journal.pbio.3002935 (PMC11649091; doi:10.1371/journal.pbio.3002935)
Supplement: S15 Fig — Example ΔEK+ traces over time for stimuli presented at 0° (top), 22.5° (middle), and 45° (bottom) following a single stimulation event. Solid lines show the data for weight-modulated orientation tuning of synapses, i.e., based on wsyn(i,j)(θ), with each synapse’s activation time drawn from a Poisson distribution with λ = 80 ms, as per S10–S13 Figs. Dotted lines show the data for frequency-modulated orientation tuning of synapses, i.e., when each synapse is activated by a Poisson train whose frequency depends on the synapse’s orientation preference (see S2 Text for details). Timing of inputs is plotted as raster plots (black lines: weight-modulated, gray lines: frequency-modulated). Following the stimulation event, the simulation runs for 2 s to show the return to baseline (ΔEK+ = 0 mV). The 2 approaches display similar dynamics, reaching same peak ΔEK+ responses for the similarly tuned segment within ∼200 ms. ΔEK+ is modulated by the stimulus orientation mainly in the segment receiving similarly tuned inputs. In contrast, the diversely tuned segments sustain a fixed ΔEK+ irrespective of the stimulus orientation, as per Fig 1g. Neighboring segments, receiving diversely tuned synapses, display a sharper decrease in the EK+ shift for the frequency-modulated input compared to the weight-modulated input. The slow decay to the steady state EK+ indicates the time window within which a second stimulation would be modulated by the elevated EK+. Colors of plotted lines are as S10 Fig. (PDF) [file pbio.3002935.s018.pdf]

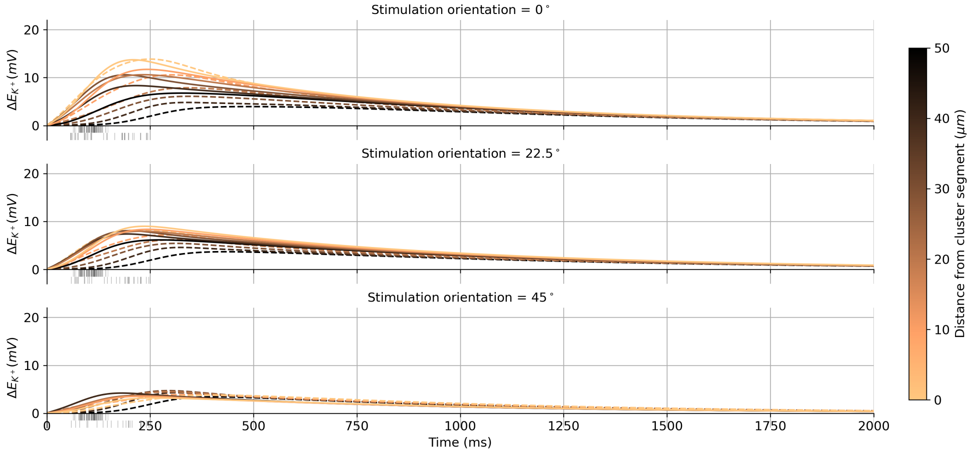


**S15 Fig: Frequency-based modulation of responses.**
Example $\Delta E_{K+}$ traces over time for stimuli presented at 0° (top), 22.5° (middle) and 45° (bottom) following a single stimulation event. Solid lines show the data for weight-modulated orientation tuning of synapses, i.e., based on $w_{syn(i,j)}(\theta)$, with each synapse's activation time drawn from a Poisson distribution with $\lambda = 80\ ms$, as per **S10-S13 Figs.** Dotted lines show the data for frequency-modulated orientation tuning of synapses, i.e., when each synapse is activated by a Poisson train whose frequency depends on the synapse's orientation preference (see **S2 Text** for details). Timing of inputs is plotted as raster plots (black lines: weight-modulated, grey lines: frequency-modulated). Following the stimulation event, the simulation runs for 2 seconds to show the return to baseline ($\Delta E_{K+} = 0\ mV$). The two approaches display similar dynamics, reaching same peak $\Delta E_{K+}$ responses for the similarly tuned segment within $\sim 200\ ms$. $\Delta E_{K+}$ is modulated by the stimulus orientation mainly in the segment receiving similarly tuned inputs. In contrast, the diversely-tuned segments sustain a fixed $\Delta E_{K+}$ irrespective of the stimulus orientation, as per **Fig 1g**. Neighboring segments, receiving diversely tuned synapses, display a sharper decrease in the $E_{K+}$ shift for the frequency-modulated input compared to the weight-modulated input. The slow decay to the steady state $E_{K+}$ indicates the time window within which a second stimulation would be modulated by the elevated $E_{K+}$. Colors of plotted lines are as **S10 Fig**.
