# Supplementary material for: Local changes in potassium ions regulate input integration in active dendrites
Source: PLoS Biol. 2024 Dec 4;22(12):e3002935. doi: 10.1371/journal.pbio.3002935 (PMC11649091; doi:10.1371/journal.pbio.3002935)
Supplement: S1 Table — (PDF) [file pbio.3002935.s019.pdf]

Channel	Conductance ($\mu S cm^{-2}$)
g_{Leak}	0.005
g_{Na_V}	5.5
g_{K_V}	.2
g_{K_M}	0.1
g_{K_A}	.9
$g_{K_{Ca}}$	.06
g_{Ca_V}	.12
g_{HCN}	.01

S1 Table: Active conductances of the point-dendrite model. Based on [1]

References

[1] Adam S. Shai, Costas A. Anastassiou, Matthew E. Larkum, and Christof Koch. Physiology of layer 5 pyramidal neurons in mouse primary visual cortex: coincidence detection through bursting. PLoS computational biology, 11(3), 3 2015.
