# Supplementary material for: Local changes in potassium ions regulate input integration in active dendrites
Source: PLoS Biol. 2024 Dec 4;22(12):e3002935. doi: 10.1371/journal.pbio.3002935 (PMC11649091; doi:10.1371/journal.pbio.3002935)
Supplement: S3 Table — (PDF) [file pbio.3002935.s021.pdf]

Channel	Channel (NEURON)	Dendrite (Scm^{-2})	Trunk (Scm^{-2})	Soma (Scm^{-2})
g_{Leak}	pas	$6{\cdot}10^{-5}$	$3{\cdot}10^{-5}$	$3{\cdot}10^{-6}$
g_{Na_P}	g_{Nap}	-	-	0.00583
g_{Na_T}	g_{NaTa}	0.021489	3.89618	0.9989
g_{K_P}	g_{K_per}	-	0.077	-
g_{K_T}	g_{K_tst}	-	0.188	-
$g_{K_{DR}}$	g_{SK+v3_1}	$18.08{\cdot}10^{-4}$	$18.08{\cdot}10^{-4}$	0.438029
g_{K_M}	g_{I_m}	$9.9{\cdot}10^{-4}$	$1.3{\cdot}10^{-2}$	-
$g_{K_{Ca}}$	$g_{SK_{E2}}$	$.02{\cdot}10^{-4}$	$.02{\cdot}10^{-4}$	-
$g_{Ca_{LVA}}$	$g_{Ca_{LVA}}$	-	$8.13\ {\cdot}10^{-4}$	$5.57{\cdot}10^{-4}$
$g_{Ca_{HVA}}$	$g_{Ca_{HVA}}$	$7.01{\cdot}10^{-4}$	$2.2{\cdot}10^{-4}$	-

S3 Table: Active conductances of the neuron model for the L5 PC neuron. Based on [1]

References

[1] Adam S. Shai, Costas A. Anastassiou, Matthew E. Larkum, and Christof Koch. Physiology of layer 5 pyramidal neurons in mouse primary visual cortex: coincidence detection through bursting. PLoS computational biology, 11(3), 3 2015.
