# Supplementary material for: Local changes in potassium ions regulate input integration in active dendrites
Source: PLoS Biol. 2024 Dec 4;22(12):e3002935. doi: 10.1371/journal.pbio.3002935 (PMC11649091; doi:10.1371/journal.pbio.3002935)
Supplement: Attachment — Submitted filename: Nordentoft_et_al_Response.pdf [file pbio.3002935.s023.pdf]

PLOS Biology Response to Reviewers

Local changes in potassium ions regulates input integration in active dendrites

Malthe S. Nordentoft, Naoya Takahashi,
Mathias S. Heltberg, Mogens H. Jensen, Rune N. Rasmussen and Athanasia Papoutsi

Correspondence:
rune.nguyen.rasmussen@sund.ku.dk
papoutsi@imbb.forth.gr

Black text = Reviewers' comments
Blue text = Authors' responses

Thank you for your careful and constructive review of our research article. We greatly value the considerable time and effort you have put into providing such comprehensive and perceptive feedback. Your insights have been crucial in refining the quality and clarity of our work. We believe we have addressed all the points raised thoroughly. Please see below for the detailed response to each point raised. For issues raised more than once, we have repeated our responses to ensure each comment is fully addressed.

1 Reviewer 1

The manuscript of Nordentoft et al. investigates the effect of extracellular potassium concentration changes on dendritic excitability and neuronal responses in abstract visual cortical neuron models. Using they modeling approach they argue that 1) the $[K]_o$ can substantially change during intense stimulation, especially in dendritic segments receiving clustered inputs; 2) The elevated extra- (and reduced intra-) cellular potassium concentration changes the reversal potential and reduces the driving force of all potassium currents in the membrane; 3) As a consequence of the reduced driving force for K, the excitability of the dendritic tree increases locally, including elongation of the NMDA spikes and increasing gain of the inputs. Importantly, the authors claim that the stongest effect can be observed when the neurons receives strong, clustered input, but even in this case, the extracellular concentration changes remain localized.
The effects are clear, and the proposed mechanism seems to be an important biophysical process potentially regulating dendritic excitability locally. However, I'm not convinced that all the spatial aspects of the changes in the potassium concentration can be neglected and that we can expect a large, but selective differences in the $[K^+]_o$ experienced by neighbouring dendritic segments.
I think that this is really the most important assumption of the paper: how much can we believe that

the changes in [K]_outside (and [K]_inside) remain localized? There are several important factors potentially influenceing $[K]$_outside not considered here:
- First, lateral diffusion. They argue in the Methods (K^+ diffusion state), that within the 10um segment they study, the $[K^+]_o$ reaches a spatially uniform concentration within 300 ms. At steady state after stimulation, the $[K^+]_o$ can be twice as large as during rest. They also argue, that different segments are well isolated from each other even on this larger time scale, since on avrage, K^+ ions only move ≈ 30 μm during a period of 600 ms. However, these arguments are not supported by exact calculations or simulations, just back of the envelop-type estimations.

We thank the reviewer for raising this issue, as we agree that we did not provide enough details in the "K^+ diffusion state" method section. We updated this section with an analytical description of our calculations, explicitly stating our assumptions and providing references for the solutions used (lines 558-568 and 578). We would like to note here that we had made an error in our calculation of the characteristic time constant (line 567, previously reported value: 263 ms), as we had multiplied instead of divided by 2 when applying equation 7. This does not affect our results and we thank the reviewer for this comment, as it allowed us to spot this error and correct it (change also made in line 481). The calculation for the spatial diffusion - distance traveled - was done using the correct equation (line 578).

I acknowedge that the fine details of many of these processes are unknown, and thus building a full model of extracellular K+ dynamics is unrealistic and beyond the scope of the current paper. However, I believe that a simple simulation of the K+ diffusion along a dendritic branch could convincinly demonstrate the spatial and temporal scales of the K+ concentration changes. Specifically, I would perform a simulation where 1. a synapse cluster (7-13 spines) is placed on the middle of a 100 μm long dendritic segment. 2. Further synapses are added with similar density but uniform tuning. 3. Synapses are stimulated with rate-modulated Poisson spike train according to a slowly changing stimulus orientation. 4. The synaptic and intrinsic currents of the dendrite are recorded and the diffusion of K+ is simulated in the extra- and intracellular space. 5. K+ reversal potential is calculated from the local concentration. 6. K/Na exchanger is also simulated to maintain the long-term balance of K+ concentration.

We thank the reviewer for this insightful suggestion. In taking on board this comment, we implemented a simple representation of an extracellular and intracellular environment, used to simulate the spatial and temporal properties of potassium diffusion. The dendrite's length, synapse population, and activation pattern were implemented according to the recommendations of the reviewer. We have further added K^+/Na^+ pumps and we used this simple model to investigate the dynamics of the $[K^+]_o$ and of the ΔE_{K^+}. Note that for these simulations, we did not assume any obstacles for the K^+ diffusion, such as astrocytes (Tyurikova et al., 2022). We have included a description of this simulation in the S2 Text and S9 Fig. and new results are shown in S10, S11, S12 and S13 Figs. These results show that a) the peak ΔE_{K^+} is reached within 100-150ms, b) the largest shift in E_{K^+} is seen at the segment receiving similarly-tuned synapses, c) ΔE_{K^+} in > 40 μm segments from the similarly tuned segment is not affected, d) E_{K^+} shifts in segments receiving similarly tuned inputs are modulated by the stimulus orientation, e) due to the differences in the sizes of the extracellular and intracellular spaces, $[K^+]_i$ minimally contributes to ΔE_{K^+}, f) increasing the rate of Na^+/K^+ pump lowers the ΔE_{K^+} both for the similarly- and diversely- tuned segments and reduces the differences in ΔE_{K^+} between the two types over time and, g) short interstimulus intervals induce similar ΔE_{K^+} levels compared to longer intervals. We highly appreciate the reviewer's constructive feedback as we believe that these additional simulations

greatly improved the quality of our work as they showcase the validity of our assumptions as well as the parameters that manipulate ΔE_{K^+} dynamics. We reference this simulation in lines 569-571 and 628-629 in the main text. Finally, regarding the levels of $[K^+]_o$ increase, this was based on experimental data showing that during sensory stimulation, motor network activity, sleep oscillations, or behavioral state transitions, $[K^+]_o$ increases by 0.25–2 mM (Kelly and Van Essen, 1974; Singer and Dieter Lux, 1975; Connors et al., 1979, 1982; Syková et al., 1974; Heinemann et al., 1990; Brocard et al., 2013; Amzica et al., 2002; Ding et al., 2016; Rasmussen et al., 2019; Dietz et al., 2023), while it can rise to 7–12 mM during hypersynchronous neuronal activity (Heinemann et al., 1977; Poolos et al., 1987; Futamachi et al., 1974) (introduction section, lines 40-45).

- Second, the K+ concentration at a given segment is influenced by K+ release from neighbouring branches, that belong to the same or a different neuron. This effect is probably hard to consider exactly, but given the high density of dendritic membranes and synapses it may have an important contribution. Estimating the number of synapses within a 5um diameter sphere centered on a particular branch gives 500 synapses (using 1.3 synapse / um3 from https://doi.org/10.1371/journal.pone.0198131). Only 2% of these belongs to the same cluster. Is it sure that we can ignore the rest? Please discuss this point.

Thank you for raising this issue. Regarding the estimated number of nearby synapses that may contribute to the local K^+ concentration change, and according to our approach in S9 Fig. , the volume around a 10 μm segment with radius (R_1) 1 μm, would be $2 \cdot \pi \cdot R_1 \cdot length \cdot height$. Assuming an extracellular space of 5 μm around the dendrite, this amounts to a volume of $2 \cdot 3.14 \cdot 1\ \mu m \cdot 10\ \mu m \cdot 5\ \mu m = 314\ \mu m^3$. The expected synapse number found in this volume is $\sim$ 300 - 400, assuming the synaptic density of 1-1.3 synapse/μm^3 (Genoud et al., 2004; Calì et al., 2018), i.e. at the same levels as the reviewer suggested. Yet, not all of these synapses are expected to contribute to the extracellular environment of the dendritic segment, as some fraction of the synapses a) are silent (Vardalaki et al., 2022), b) driven by non-visual events (Knöpfel et al., 2019), c) driven by different stimulus orientations or d) display small trial-to-trial correlations (Scholl et al., 2021). Taking into account the role of astrocytes in creating and controlling extracellular micro-environments (Halassa et al., 2007; Chung et al., 2015; Tyurikova et al., 2022), we anticipate that the number of synapses contributing to the $[K^+]_o$ hotspots will be of the order of 10's, as used in this study. Importantly, we cannot rule out that spatiotemporally synchronized activation of synapses located in different dendrites of the same or different neurons could contribute to the $[K^+]_o$ of a small dendritic segment, or, vice versa, that the creation of $[K^+]_o$ hotspots could influence synaptic responses in different dendritic segments. This potential co-activation could either increase the noise levels, or could also promote the synergy between dendrites and/or neurons (e.g. for inputs formed by the same enpassant axon or association of events). We now discuss these alternatives in the discussion section, lines 460-464.

Specific comments:
Figure 1: panel labels are CAPITAL letters, references are small.

We have updated the panel labels in the figure with lowercase letters.

Fig 2c: does the resting potential also change after the elevation of the $[K]_o$? Is this responsible for the shift of the curves before stimulation?

This is correct, for these simulations, we modeled the activity-dependent local increase in $[K^{+}]_o$ as a step function in between events: The first stimulation event was regarded as the control condition with $\Delta E_{K+} = 0\ mV$ and the two subsequent events as the condition for a given E_{K+} shift in the interval $\Delta E_{K+} \in [6 : 18\ mV]$ (described in methods section, lines 676-679). This E_{K+} shift is also visible in the membrane potential of the dendritic segment and we have now clarified this in the caption of Fig. 2c. In addition, we also now show the complete voltage traces for the three stimulation events in S1 and S2 Figs.

Page 6: "we also included a similar test using a reconstructed neuron morphology based on a previously published model, see (Supplementary Figure 4)." -> SF5.

As new Supplementary Figs. were generated as part of the revision processes, we have updated the references accordingly throughout the manuscript.

2 Reviewer 2

Nordentoft and colleagues explore the role of the transient changes in intra- and extracellular potassium concentrations for dendritic (and neuronal) integration of synaptic inputs. Based on a dendritic model and a simplified neuronal model with more realistic morphological structure, the authors show how co-tuned excitatory inputs may elicit stronger outputs when changes in the reversal potential of potassium channels are taken into account. Complementary, the authors show that dynamically controlling this reversal potential can effectively modulate the input/output function of both dendrite (local membrane potential) and neuron (output spikes), revealing an interesting new role for potassium channels. To the best of my knowledge, this is a novel approach to elucidate the role of ionic concentrations (and their dynamics) for neuronal processing. I really like the approach and appreciate the modelling of both analytical, point-dendrite, and morphological neurons. In my view, the results are novel and relevant, and would be of interest to the broad neuroscience community. However, some points should be addressed by the authors to clarify the origins of some of the phenomena presented in their manuscript. For example, the model used by the authors comprises four different potassium channels with distinct dynamics described by activating and inactivating gates, and the role of each of these channels for the modulation on the input/output function is not clear. Additionally, it's not clear whether the effect reported by the authors could also be achieved by strengthening synapses. I elaborate on this and other major and minor points below.

2.1 Major

1) Focus on the visual cortex. As I was reading the manuscript I would often think that the author's approach and results would fit very well the description of many other brain regions rather than visual cortex only which is only mentioned briefly in the discussion section. I think that the manuscript would benefit greatly if the model and results were built in a more general framework from the beginning, using the visual cortex as one of many examples given that other cortices (and even hippocampus if place fields are considered) have, in general, tuning curves.

We agree with the reviewer that our results are relevant to multiple brain regions, potentially providing a general principle on the regulation of input integration in dendrites receiving tuned clustered input. In this work, we used the visual cortex as the framework of study as this is a well-studied region with multiple experimental data that are highly relevant to this study. These include the tuning properties of synaptic inputs (Chen et al., 2013), detailed characterization of dendritic segments receiving inputs with similar or diverse orientation tuning (Wilson et al., 2016; Jia et al., 2010; Iacaruso et al., 2017; Petousakis et al., 2023; Park et al., 2019), *in vivo* experimentally recorded changes in the $[K^+]_o$ in a state-dependent manner, and regulation of the visual responses by these $[K^+]_o$ changes (Rasmussen et al., 2019). Basing our simulations on these experimental data was important, as it provided credibility to our results while describing a previously unknown phenomenon of dendritic integration. Yet, we agree that only including the potential cortex-wide application of our results in the discussion section conceals the impact of our work. To account for this we have now included a discussion of this issue in the introduction (lines 88-94), motivating the general application of our work.

2) Lack of inhibitory synapses. Until the discussion section, the authors refer to the connections in their model simply as "synapses", without specifying their types, and I think it would be important to clarify already in the introduction and beginning of results section that they are only considering the activation

of excitatory synapses. Additionally, I think that a more detailed description of the rationale for not including inhibitory connections is lacking. Why was it not included? Is it because another set of free parameters would have to be explored, which prevents a more detailed description of the phenomena? Would it be possible to consider at least one case in which inhibitory inputs are unspecific?

Thank you for raising this issue. In this work we choose to focus on the K^+ efflux from NMDA excitatory synapses and the effect of clustering of excitatory synapses on dendritic segments, based on the available experimental data and previous modeling work (Chen et al., 2013; Wilson et al., 2016; Jia et al., 2010; Iacaruso et al., 2017; Petousakis et al., 2023; Park et al., 2019; Rasmussen et al., 2019; Shih et al., 2013; Tyurikova et al., 2022). As pointed out by the reviewer, the lack of experimental data regarding inhibition and extracellular K^+ concentration would greatly increase our parameter space and limit the number of conclusions we could draw from this work. We acknowledge that this is a limitation of our work, and we discuss this in lines 464-468. Following the reviewer's point, we have also repeated the simulation of Fig. 2b, while implementing an E_{K^+} shift following the first stimulation event, in the presence of GABAergic inhibition (S1 and S2 Figs.). Results show that non-specific inhibition does not alter the effect of E_{K^+} shift in regulating dendritic integration. Overall, we have made the following changes related to this point: a) Introduction: line 76 (clarification that only excitatory synapses were simulated), b) Results section: lines 225-226 (reference of the non-specific inhibition simulation results), c) method's section: lines 634-637 (description of the simulation including inhibition), and d) S1 and S2 Figs. and equation for $GABA_A$ receptors at S1 Text.

3) Extracellular compared to intracellular volume. The authors consider a fairly small extracellular volume compared to the intracellular one (e.g., Fig. 1g,h) and refer to previous work for this choice. However, as far as I understood, Tønnesen et al. (2018) have shown that the intracellular volume is bigger than the extracellular volume when considering a region with many somas (and adding up all the different neurons). For a single (and small) dendritic compartment (far from the soma), I would expect a much larger extracellular volume compared to the intracellular one. Considering that the extracellular volume (defined by a cylinder with radius R2 and length L) is a free parameter of the model, I would like to suggest checking the values of $\Delta[K^+]_o$ and $\Delta[K^+]_i$, and consequently ΔE_{K^+}, for two extreme conditions: infinite and zero extracellular volumes.

We thank the reviewer for this insightful comment, as it is very important for our results. Tønnesen et al., 2018 report that the volume fraction of the extracellular space is on average $\sim 20\%$ of the total space. Yet, as the reviewer highlights, there is layer-specific heterogeneity with values ranging from 5% to 36%, including the less somata-enriched layers (Fig. 3d, 3e in (Tønnesen et al., 2018)). Importantly, the actual width of the extracellular space has a median value of 0.27 μm (Fig. S3I in (Tønnesen et al., 2018)), with the majority being between 0.1-0.3 μm (Fig. 3g in (Tønnesen et al., 2018)). A recent review (Tønnesen et al., 2023) highlights that although the average width of the extracellular space could be even lower, around 40-80 nm, it can range from small gaps of 10-20 nm to larger spaces in the range of 0.2-0.5 μm. Even for these larger spaces, and considering that the dendritic diameter of the apical and basal dendrites of pyramidal neurons is in general >0.5 μm (Gilman et al., 2017; Benavides-Piccione et al., 2020), the extracellular space is on average smaller than the intracellular one. Following the reviewer's suggestion, we used equation 1 of the main text to evaluate $\Delta[K^+]_o$ in the limit where radius R_2 goes towards R_1 and infinity. Note that R_2 cannot be smaller than R_1 by definition. Given that R_2 is only in the denominator, as it goes towards R_1 (i.e. extracellular space becomes smaller), $\Delta[K^+]_o$ will increase as $\frac{1}{x}$ and becomes

undefined for $R_2 = R_1$. Conversely, $\Delta[K^+]_o$ will go asymptotically towards 0 as R_2 goes towards infinite. We have now added a discussion about this in lines 174-175.

Importantly, following the reviewer's comment, we noticed that we had not highlighted the importance of the extracellular space size in our manuscript, especially considering that the extracellular space itself is a dynamic compartment with timescales ranging from hours to seconds; for example, the extracellular space decreases in response to epileptiform activity. These structural changes can be highly local, as shown by 2-photon glutamate uncaging experiments (Tønnesen et al., 2018). These local structural changes in response to physiologically relevant stimuli may constitute another way for local modulation of neural activity and information processing. Given that this is a very important issue not highlighted in our initial submission, we now discuss it in the discussion section (lines 468-475).

4) Role of individual channels. The authors implement four different potassium channels in their models, each one with distinct activation and inactivation functions according to the Supplementary Material. First, it would be interesting to discuss whether changes in molar concentrations may have an effect in the activation and inactivation functions of these channels - is there any reference for that? Second, I think it would be important to pinpoint the effect of each ion channel. One question that naturally arises is whether the seemingly independence of changes in ΔEK^+ on volume ratio (Supplementary Fig. 4) is due to the combination of the four ion channels or not. This could, e.g., explain the discrepancy with sodium and calcium channels, which seem to follow very similar trends (Supplementary Fig. 4).

Regarding the first point, following literature research we identified two types of potassium channels that are modulated by the molar concentrations of K^+ ions. First, the voltage at which inward-rectifier K^+ (Kir) channels open depends on the $[K^+]_o$ (Hille et al., 1992). Although these channels are mainly found in non-neuronal cell types, they have also been characterized in several neuronal types, mainly in subcortical regions (Hibino et al., 2010). There is also evidence that the slow delayed rectifiers (KCNQ1) voltage-gated K^+ channels can be modulated by the $[K^+]_o$ (Abrahamyan et al., 2023; Larsen et al., 2011). Nevertheless, we are not aware if these cases are applied in the visual cortex studied here; for example, the studies on the modulation of KCNQ1 channels by the $[K^+]_o$ mainly focus on strial marginal cells or the gastrointestinal organs where these channels are abundantly expressed (Abrahamyan et al., 2023). Following the point raised by the reviewer we have included a discussion of the possibility of K^+ channel activation/inactivation modulation by the $[K^+]_o$ changes (lines 456-459). In the case that we have overlooked relevant studies that the reviewer is aware of, we would be glad to further update the discussion section accordingly.

Regarding the second point raised, in our work, the driver of the $[K^+]_o$ changes is the NMDA receptors. This is based on experimental studies showing that the major source contributing to the $[K^+]_o$ changes at the synaptic level creating $[K^+]_o$ hotspots is the K^+ efflux from excitatory synaptic receptors, when compared to the calcium- or voltage-dependent potassium channels (Shih et al., 2013) (Tyurikova et al., 2022). These studies have shown that such postsynaptic NMDA receptor-mediated K^+ efflux is highly localized, pointing to synaptic activity-dependent local $[K^+]_o$ changes acting as a self-modulator of synaptic transmission. Importantly, this comment gives us the chance to clarify our approach, which we consider one of the strengths of this work, not sufficiently highlighted in our initial submission. Specifically, in the S3 Text "Electrochemical potential shifts of ions", and S14 Fig. (4 in our initial submission), we show that the effect of raising the extracellular potassium concentration, irrespective of the source of this rise, is expected to have a greater impact on its reversal potential, compared to changes in Na^+ or Ca^{2+} concentrations. Given this differential effect of K^+, compared to Na^+ or Ca^{2+} when ion con-

centration gradients change, localized, activity-dependent changes in the K^+ concentration, irrespective of the specific channel source, will result in high changes of the E_{K^+}. The differential sensitivity of the K^+ reversal potential compared to the reversal potential of Na^+ and Ca^{2+} ions is a direct effect of the Nernst equation and the high K^+ intracellular concentration, compared to a high extracellular Na^+ and Ca^{2+} concentration.

To highlight the reason for this differential sensitivity and the importance of the extracellular space, we elaborate in the response to reviewers a practical example for the extreme case that the intracellular and extracellular Na^+ and K^+ concentrations are equal (but reversed), i.e. $[Na^+]_o = 145\ mM, [Na^+]_i = 5\ mM$ and $[K^+]_o = 5\ mM, [K^+]_i = 145\ mM$. According to Nernst equation, in this case, $E_{Na^+} = -E_{K^+} = 90.24\ mV$. When the same number of ions move along the intra/extracellular spaces, e.g. of increasing (decreasing) the extracellular concentration of K^+ (Na^+) by $5mM$, and assuming $V_R = 10$ (i.e., $V_i = 10 \cdot V_o$), the new concentrations will be: $[Na^+]_o = 145\ mM - 5\ mM = 140\ mM, [Na^+]_i = 5\ mM + \frac{5}{V_R}\ mM = 5.5\ mM$ and $[K^+]_o = 5\ mM + 5\ mM = 10\ mM, [K^+]_i = 145\ mM - \frac{5}{V_R} = 144.5\ mM$. The new E_{Na^+} will be $86.74\ mV$, corresponding to a $\approx 4\%$ change of the reversal potential and E_{K^+} will be $-71.57\ mV$, corresponding to a $\sim 21\%$ change in the reversal potential. The dependency on volume ratio for the case of Na^+ channels is seen if we set $V_R = 2$ in this practical example; in this case, increasing (decreasing) the extracellular concentration of K^+ (Na^+) by $5\ mM$, results in $[Na^+]_o = 145 - 5 = 140\ mM, [Na^+]_i = 5 + (5/V_R) = 7.5\ mM, [K^+]_o = 5 + 5 = 10\ mM, [K^+]_i = 145 - (5/V_R) = 142.5\ mM$, yielding $E_{Na^+} = 78.43\ mV$, corresponding to a $\sim 13.1\%$ change of the reversal potential and $E_{K^+} = -71.2\ mV$, corresponding to a $\sim 21.1\%$ change in the reversal potential, similar to the E_{K^+} change assuming $V_R = 10$. We have now added a description of this effect in S3 Text. Overall, this differential sensitivity of the E_{K^+} to changes in the K^+ concentration, along with its invariance in the dimensions of the extracellular space, highlights the impact and potential generalization of our work. Neurons are more sensitive to the local, activity-dependent elevations of the extracellular K^+ concentration, because of the basic concentration gradients and geometry along the membrane of dendrites. This sensitivity of the K^+ reversal potential, along with the experimental evidence for localized $[K^+]_o$ hotspots (Shih et al., 2013; Tyurikova et al., 2022) and the *in vivo* experimentally recorded, state-dependent $[K^+]_o$ changes (Kelly and Van Essen, 1974; Singer and Dieter Lux, 1975; Connors et al., 1979, 1982; Syková et al., 1974; Heinemann et al., 1990; Brocard et al., 2013; Amzica et al., 2002; Ding et al., 2016; Rasmussen et al., 2019; Dietz et al., 2023), place this ion as a key regulator of dendritic integration and neuronal processing.

5) Dynamics of EK+. One of the main assumptions is that "1) Local increases in $[K^+]_o$ are proportional to synaptic activity level." If that's the case, I would interpret that ΔE_{K^+} is dynamic and follows the activation of AMPA/NMDA channels (maybe with some slow time constant). For the statistical analysis, assumption "3) $[K^+]_o$ change is stable in space and time along the short dendritic segment." is a valid (and necessary) one, but for the dendritic dynamics this assumption might be too strong. From my understanding, both point-dendrite and morphological neuron were implemented with fixed E_{K^+} for different dendritic segments based on whether the connections onto them are co-tuned or not. I find it to be a very good initial assessment of the dendritic response, but it is ultimately at odds with assumption (1) because inputs are not constant (allowing for definition of co-tuned), which means that ΔE_{K^+} should increase when the target orientation is active, and go to zero otherwise. In Fig. 2c, it's clear that the baseline Vm (when no input is present) is different for different ΔE_{K^+} and, according to assumption (1), they should all start from the same resting state if other orientations were active beforehand. Is there strong evidence for the target orientation being activated consistently with a maximum of 300 ms delay?

What would happen if ΔE_{K^+} is dynamic?

We thank the reviewer for these questions. Indeed, as the reviewer highlights, previous activation of the same dendritic segment is a necessary condition for the regulation of dendritic integration by the $[K^+]_o$ hotspots. The time window for this interaction is of the order of seconds (Benninger et al., 1980; Aitken and Somjen, 1986), due to the slow recovery of $[K^+]_o$. By describing $[K^+]_o$ changes as proportional to synaptic activity levels, we aimed to emphasize exactly this requirement, i.e. that the overall level of activity of a dendritic segment is an important factor for the creation of the $[K^+]_o$ hotspots.
To show how the long time constant of $[K^+]_o$ recovery is applied to our results, and following also Reviewer's 1 point 2, we implemented a simple representation of an extracellular and intracellular environment that we used to investigate the spatial and temporal properties the diffusion of the extracellular potassium and the dynamics of ΔE_{K^+} (S2 Text and S9-13 Figs. 9-13). Please see also our response to Reviewer's 1 point 2. As shown in S10 Fig., and according to the reviewer's intuition, ΔE_{K^+} does follow the dynamics of synaptic activity with a slow time constant. Yet, when comparing ΔE_{K^+} between different stimulus orientations (S10 Fig., top, middle, and bottom panels), or between dendritic segments receiving similarly-tuned (S10 Fig., top panel, yellow trace) and diversely-tuned (S10 Fig., top panel, green trace) inputs, it is visible that there are, on average, long-lasting differences in this cases. These differences persist when manipulating the interval between the stimulus events (S13 Fig.). Given that dendritic integration operates in time scales of a few milliseconds to hundreds of milliseconds in the case of NMDA spikes, we chose to implement this time scale separation due to the different time constants of the described phenomena as it has been successfully applied previously on similar questions (Contreras et al., 2021). Clamping the ΔE_{K^+} to a constant value, allows us to investigate the effect of the different ΔE_{K^+} in isolation from other parameters that would be hard to validate (e.g. strength and kinetics of K^+ pumps - S12 Fig.). This is a simplification of the problem in hand, that we believe that is now justified by our additional simulations and parameter exploration. In addition to the inclusion of the new section and simulation in the S2 Text, we further highlight the necessity, and physiological importance, of previous dendritic activity in lines 411-418 of the main text.
Note that in Fig. 2c the first stimulation event at a specific orientation was regarded as the control condition with $\Delta E_{K^+} = 0\ mV$, and the two subsequent events included an E_{K^+} shift in the interval $\Delta E_{K^+} \in [6 : 18\ mV]$ (described in methods section, lines 629-632), based on the orientation presented in the first stimulus event (i.e. we assumed repetitive activation with the same stimulus). This E_{K^+} shift is also visible in the membrane potential of the dendritic segment (now clarified in the caption of this figure). To make our approach more clear, we have also included a new figure (S1 Fig.) where we show the complete voltage traces for the three stimulation events.

6) Functional consequences. The authors explore the functional consequences of changes in local $[K^+]_o$ by the modulation in postsynaptic firing. These results seem to align very well with the dendritic spike probability (comparing Figs. 2g and 4c) which leads me to the following question. Is the modulated postsynaptic response for greater dendritic distances to the soma (Fig. 4g) solely a consequence of the changes in potassium concentration throughout the dendritic tree (as stated by the authors) or a consequence of a greater local response? In summary, is the postsynaptic response the same for (i) strong synapses and no potassium modulation and (ii) weak synapses and potassium modulation? This also leads to another question regarding synaptic weights below.

Thank you very much for this comment. Following this point, as well as points 7 and 22, we have

performed two comparisons of the strength of synaptic activation and the $[K^+]_o$ change in modulating dendritic excitability: First, we evaluated how varying the average synaptic activity and the number of synapses shapes the dendritic spike threshold, similarly to Fig. 2f. In the new S5 Fig., we see that, as expected, increasing the number of synapses also increases dendritic excitability. Second, we simulated orientation tuning for different strengths of synaptic activation in the abstract neuron model, shown in S8 Fig. The response at the soma is similar to Fig. 4c, as per the reviewer's intuition. We now discuss these plots in lines 349 of the results section, as well as, following point 7 below, in the discussion section, lines 411-417.

7) Parameter set. Eq. 2 describes the approximated combination of parameters ΔE_{K^+}, N, and w, namely, change in potassium reversal potential, number of synapses, and synaptic strength, that allow the local dendrite to trigger dendritic spikes. The authors found a linear relationship for these parameters, which intuitively would mean that distinct combinations of these parameters would give rise to the same results. In this case, what would be the resulting plot in Fig. 2f for different synaptic weight values or number of synapses? If the x-axis of Fig. 2f is synaptic weight, w, would it look equivalent? If that's the case, I would like to suggest elaborating on the benefits of having changes in the potassium reversal potential if, e.g, having strong weights would have the same final result (e.g., in Fig 4).

Indeed, given the linear relationship between the 3 parameters (S3 and S4 Figs.), the effect of increasing the number of synapses has similar effects to ΔE_{K^+} (S5 Fig.). This also has similar functional consequences for orientation tuning (S8 Fig.). This equivalency highlights the benefit of our proposed mechanism: it constitutes an energetically favorable way to increase the gain of local integration with minimum usage of resources, compared to long-term plasticity mechanisms. Importantly, the ΔE_{K^+}-dependent change in gain is transient, operating in time scales of several seconds, selectively boosting the activity of repetitively active sections. As such, $[K^+]_o$ follows the overall activity levels. We have previously shown that $[K^+]_o$ shifts are state-dependent, e.g. on whether the animal is in quiescence or locomoting (Rasmussen et al., 2019), indicating that state-dependent modulation of sensory processing may be supported by $[K^+]_o$ changes following the overall activity levels. We are discussing the above in the discussion section (lines 411-417).

2.2 Minor

8) I would like to suggest to the authors to submit PDFs with lines numbered so it's easier to address specific points during the review process.

We apologize for this oversight in our initial submission. We have now included line numbers in our revised version.

9) Vm is used once in the Introduction, thus I suggest writing "membrane potential".

As we are also using Vm in other places in the manuscript (e.g. caption of Fig. 2 and the methods section), we would prefer to leave the definition here.

10) Page 2: "These dendritic spikes support the nonlinear..., which in turn boost the neuron's input-output function..." Do the authors mean "modulate the neuron's input-output function"? The output can be boosted, but the function can only be modulated (or another synonym for modulation).

We thank the reviewer for spotting this error. We chose the word "alter", as it shows that it changes the function (e.g. from linear summation to sigmoidal with dendritic spikes). The change has been made in line 67.

11) Page 2: "We focus on the organization of synaptic inputs tuned..." These are excitatory synaptic inputs. I would like to suggest clarifying this point.

We have now included the clarification (line 76).

12) Page 2: "... synaptic inputs can attain substantially higher [K+]o changes ... These [K+]o elevations ..." In the first sentence the authors use "higher changes" and later "these elevations". There is a mismatch here.

We have changed the wording in the text (line 82).

13) It was not clear from the first paragraphs how the model was initially implemented. It was more clear after reading the subsequent section which described the point-dendrite model. If I understood correctly, all results presented in Fig. 1 are not taken from the implementation of the membrane potential dynamics, but from a statistical analysis. I would like to suggest clarifying that initially no dynamics were included in the model. I think this is a very interesting and powerful way to analyse such a system, and as so, deserve to be highlighted.

The reviewer is correct on this point (also relevant to comments 14, 24, and 31). In retrospect, we understand that we did not explicitly comment on our approaches in our submitted manuscript, making it difficult for the reader to follow the different models implemented throughout this work. Importantly, we also agree that results from the different levels of abstraction and details greatly increase the power of our results. We have thus restructured our text to make explicit our methodological approaches. Specifically, we have done the following modifications:
1. (Introduction) Lines 79-80: We outline the different types of models implemented.
(Results) Lines 99-100, 103, and 198 now highlight the different approaches implemented.
3. (Methods) Line 492: We changed the title from "Orientation-tuned synapses of dendritic segments" to "Sampling of orientation-tuned synapses of dendritic segments", to show that this is a statistical approach.
4. (Methods) Lines 508-510: We added a comment that we used the same sampling method throughout our models.
5. (Figures) Fig. 1c: We clarify that we did not model membrane dynamics in this approach.

14) Page 3: "... we implemented 10 μm segments ..." Following the point above, the word "implementation" gave me the impression that the model being presented here had membrane potential dynamics. It was also not clear to me whether these were supposed to represent isolated segments (like in the pound-dendrite model) or part of a morphological neuron. I would like to suggest clarifying these points.

Following comments 13, 24, and 31 we have modified our text to highlight our statistical approach in the Introduction, Result, and Method sections, as well as in Fig. 1c caption (see detailed response in comment 13). For ease of the review process, we state here that for this comment, the change is in lines 103.

15) Page 4, Eq. 1: Isn't there a minus sign missing here? Positive changes outside should reflect negative changes inside and vice versa, so both deltas should have opposite signs.

Thank you for pointing this out. As we would like to keep it positive, we have redefined it using the absolute value of $\Delta {K^+}_i$ (equation 1). This is taken into account in equation 2 when calculating the change in the Nernst potential.

16) Page 4: "... by converting the $\Delta[K^+]_o$ into shifts in E_{K+} ..." I would like to suggest adding the equation used to convert one into the other here for clarity, given that this is an important implementation throughout the paper.

Following also comment 33, we have moved this equation to the results section (now equation 2). We have also included a description of how we used this equation to calculate the ΔE_{K+} (lines 189-194).

17) Page 4: "Synaptic inputs were activated by delivering a stimulation train consisting of three events..." The precise implementation was not clear to me. The equations for AMPA and NMDA channels in the Supplementary Material lack additional information for how these activity patterns were implemented. I would like to suggest a more detailed explanation in the Methods and/or Supplementary Material.

Following also comment 41, we have added a description in S1 Text regarding the activation of NMDA and AMPA receptors, as well as how the AMPA and NMDA receptors were stimulated.

18) Page 5, Eq. 2: I could not find the parameter values for α, β and ν. I would assume that β is negative - is it the case? Would that mean that ΔE_{K+} can be negative for very strong weights? How was w chosen?

We would like to apologize for omitting to include the values for the α, β, and ν fitted parameters. We have now included these values as a table in the methods section. We further highlight the cases that could result in negative ΔE_{K+}. For these cases, we assumed to correspond to no E_{K+} shift. Changes were made in lines 647-661. Regarding w, it corresponds to the average synaptic activity, depends on the synaptic tuning curves and synaptic distributions as well as on the presented orientation (Fig. 1d, 1e), and takes values between 0-1. We have now clarified this in the method section, lines 516-517.

19) Page 5: "The current barrier..." Isn't it the "voltage barrier"? The analysis of the system through fixed points is given by checking which values of Vm result in dVm/dt = 0, shown in Fig. 3. The neuron model is defined as dVm/dt = I, so that's why the analysis also works considering the current on the y-axis, but the definition of a fixed point is for dVm/dt. Same for the sentence "... it lowers the current barrier..." Isn't it "voltage barrier" here as well?

We thank the reviewer for pointing this out. We have now changed the wording from current barrier to

voltage barrier in (lines 276 and 302). Please see also our response to comment 28 below for the reasons we chose to use the inverted current values on the y-axis.

20) Page 6: "... segments of the dendritic tree randomly underwent E_{K^+} shifts . . . " Why is it random? One of the assumptions to build the model according to the authors is "1) Local increases in $[K^+]_o$ are proportional to synaptic activity level.". Following this argument, changes in E_{K^+} should not be random, but proportional to the AMPA/NMDA activation in each dendrite. I would like to suggest clarifying this point and including a more detailed implementation in the Methods/Supplementary Material.

By imposing E_{K^+} shifts randomly in dendrites we aimed to take into account previous widespread dendritic activity that could affect the transfer of the depolarization of the stimulated, distal dendrites towards the soma. We understand that this choice reduces the clarity of our work and creates reasonable confusion, without making a substantial contribution to our results. To comply also with the reviewer's comment, we re-simulated the results shown in Fig. 4 without the random E_{K^+} shifts and we now show the results with only the dendritic segments receiving the inputs undergoing E_{K^+} shifts (change made in line 317). We have likewise updated the methods section with this change in protocol (lines 705-707). The results discussed in the manuscript remain the same after this change in the protocol.

21) Page 6: ".. during a synaptic stimulation burst." I would like to suggest including details of the implementation in the Methods/Supplementary Material, e.g., duration of burst, number of spikes, etc.

We have changed the word "burst" to "event" (lines 355, 628), as we think that the latter better describes our stimulation protocol. The stimulation protocol is described in lines 625-628 in the methods section. Please note that a typo declared this interval as 500ms in our previous version which is now corrected (line 627).

22) Page 6: "... due to the local gain of the synaptic input, the E_{K^+}-shifted regimed is attenuated less with distance." If synaptic strengths were stronger at these dendrites, would the effect be the same without shifting E_{K^+}? If that's the case, synaptic plasticity could be optimising the connections so that the effect is larger without the need of E_{K^+} changes.

We thank the reviewer for this insightful comment. Following points 6 and 7, we have evaluated the effect of increasing the number of synapses compared to ΔE_{K^+} in the point-dendrite (S5 Fig.), and in the abstract neuron model (S8 Fig.). We have also included a discussion of these points in lines 411-418. See our response to points 6 and 7 for a more detailed response to this issue.

23) Page 6: "... attenuated with a factor L1/3". How was this value calculated?

We used the chi-square method to fit a power law function to each of the different ΔE_{K^+} shifts. To compare the attenuation for large and no ΔE_{K^+} shift we divided the two fits. This results in a new power law function, with the exponent corresponding to the difference of the large and no ΔE_{K^+} shift exponents, that describes the difference in attenuation in the two conditions. We have added this description in lines 371-371.

24) Fig. 1: If I understood correctly, all results presented in this figure were calculated/estimated with-

out simulating the membrane potential dynamics explicitly. For clarity I would like to suggest writing this explicitly in the caption so that it is easier for the reader to immediately understand the difference between, e.g., Figs. 1 and 2.

As part of comments 13, 14, and 31 we have adjusted our manuscript to highlight our analytical approach. Detailed responses are provided under comment 13. To ease the review process, we state here that for this comment, the change is in the caption of Fig. 1c.

25) Fig. 1, panel c: If I understood correctly, panel c shows an integration of the inputs over a large period in time, and not a snapshot (otherwise the synapses would not represent different orientations). I would like to suggest clarifying this point in the caption.

The reviewer is correct, the synaptic tuning curves are the results of expected activity for a given stimulus orientation (as per Figure 4 of (Chen et al., 2013)). We have changed the description of Fig. 1c caption to increase clarity.

26) Fig. 2, panel h: The y-axes are different for peak and orientation. Starting the orientation axis at zero while starting the peak axis at 0.4 visually amplifies the effect of the changes in peak spike probability. I think it would be more of a fair comparison to use the same axis range for both.

Following the reviewer's recommendation we have modified the second axis to start at 0.

27) Fig. 3, arrows: The arrows in panels a-d are hardly visible. I would like to suggest either removing them or making them more visible.

We have doubled the arrow sizes in Fig. 3 to increase visibility.

28) Fig. 3, current: For the analysis of fixed points, the usual approach is to compare dVm/dt against Vm. This way it's clear that: 1) when the line crosses zero, there is no change (it's a fixed-point); 2) when the line is above zero, changes are positive, so Vm is moving to the right; and 3) when the line is below zero, changes are negative, so Vm is moving to the left. I find it a bit confusing that the authors plotted negative values above positive ones, inverting the usual presentation of fixed-point analysis. I would like to suggest changing the plot, or clarifying this in the text and caption.

We thank the reviewer for this comment. The reason for choosing these axes is that we find the current-voltage relationship more intuitive for the non-experts in dynamic systems, compared to the phase plot of the membrane potential. The format of this figure was inspired by the (Major et al., 2013) analysis (their Fig. 3 and Supplementary Fig. 4) and would like to keep the same style for this plot. To increase clarity, as well as make the Fig. 3a-d plots more intuitive, we have a) altered the text to include a more thorough description of the $\frac{dV_m}{dt}$ and I relation (lines: 271-272), b) included a description of the y-axis in the Fig. 3 caption, and c) we have changed the axes to show current direction (inward/outward) to make the plot more clear.

29) Fig. 4, panel d. Similarly to Fig. 2h: one y-axis starts at zero and the other above zero, highlighting one effect more than the other.

We have altered the frequency axis to start at 0 in Fig. 4d.

30) Page 7: "First, the fine-scale arrangement of orientation-tuned synaptic inputs determines the magnitude of activity dependent $[K^+]_o$ changes..." If I understood the model correctly, this was not a result, but an assumption used to build the model. I would like to suggest rephrasing this sentence to reflect this point.

The reviewer is correct and we have revised this sentence accordingly (lines 379-380).

31) Page 13: The two first sections, if I understood correctly, describe the model without any voltage dynamics. I would like to suggest clarifying this point.

As part also of comments 13, 14, and 24 we have adjusted our manuscript to highlight our approaches. A detailed response is provided under comment 13. To ease the review process, we state here that for this point, the change is in line 492 and lines 508-510.

32) Page 14, Eqs. 6 and 7: As for Eq. 1, isn't there a negative sign missing here?

Thank you for pointing this out, we have changed the equation to include the absolute term of $\Delta[K^+]_i$.

33) Page 14: I would like to suggest putting Eq. 8 in the Results section as well to help the readers understand the implementation.

Following also reviewer's point 16, we have moved Nernst's equation to the results section (now equation 2). We have also described how we convert $\Delta[K^+]_i$ and $\Delta[K^+]_o$ to ΔE_{K+} using Nernst's equation (lines 189-194).

34) Page 15: I would like to suggest including all terms in Eq. 11 for completeness.

We have now included all the terms in line 615 (previous equation 11 is equation 12 in the revised manuscript).

35) Page 15, Eq. 12: The total conductance should include the gating terms (n, m, h, etc). I would like to suggest clarifying this point.

We have added the complete terms in the denominator of Eq. 12 (previous equation 11) and expanded here on the first terms (line 623).

36) Page 15: "The orientation tuning... as we have done previously." I would like to suggest expanding on the details of the implementation for completeness.

We have expanded on our method in line 624-626.

37) Page 15: "... mean synaptic activity (w..." The variable w is not defined for the implementation of

the point-dendritic model nor the morphological model. I would like to suggest defining it.

We have added the definition of w in lines 516-517.

38) Page 16, Eq. 13: What are the values for α, β and ν?

We would like to apologize for not including these values in our initial submission. Following also reviewer's point 18, we have added the fitted values in Table 1 in the methods section, accompanied by a discussion of the fitted values (lines 656-661).

39) Page 18, Eq. 16: The definition of orientation selectivity seems to differ from previous work where it is defined as $1 - CV = |\sum_k \frac{rke^{i\theta_k}}{\sum krk}|$. I would like to suggest double checking this definition.

We thank the reviewer for spotting this. We have updated equation 17 (previous equation 16) with the correct formulation of the equation and we have also updated the OSI values, to be consistent with this formulation (lines 762-764).

40) Page 2: "Common for all intrinsic channels is that they conduct a single ion species." Later the authors define an intrinsic ion channel with mixed ion conductance (HCN). I suggest clarifying this point.

We have clarified this in S1 Text.

41) Page 4: When inputs are active, the AMPA and NMDA receptor dynamics are not clear to me. What is changing in these equations when inputs are present?

Following the reviewer's point, we have added a description of how synaptic receptors were implemented and describe how the temporal dynamics were simulated (S1 Text).

42) Supplementary Fig. 1: I found it hard to see whether the points were indeed on that plane. For clarity I would like to suggest adding 2D plots where one of the variables is fixed. For example, using the y-axis as EK^+, x-axis as w, and different lines for different values of N. This might help to visualise whether a plane is capturing the points well.

We have included the 2D plot in S4 Fig., in addition to the 3D plot (current S3 Fig., previous Supplementary Fig. 1). In this 2D plot, the linear relationship of the 3 parameters is better visualized.

43) Supplementary Fig. 3: I find it interesting that the peak firing-rate decreases as the fraction of similarly-tuned segments increase as I would expect it to be the opposite. It would be interesting to discuss these results and maybe even add to the main manuscript.

Following the reviewers' point 20, we decided to remove this approach from our work, as randomly changing ΔE_{K^+} was not consistent with our assumptions. Our initial aim for imposing E_{K^+} shifts randomly in dendrites was to take into account previous widespread dendritic activity that could affect the transfer of the depolarization of the stimulated, distal dendrites towards the soma. We understand that this choice reduces the clarity of our work and creates reasonable confusion, without making a substan-

tial contribution to our results. To comply with both the reviewer's comments and our assumptions, we repeated these simulations where only the segments receiving input also undergo E_{K^+} shift. In that regard, the referenced figure in this comment is no longer valid, and we have thus decided to remove it, as well as its description from the Results section.

44) Page 9: Isn't the equation for ΔEion with the wrong sign? I would expect it to be after - before, which is how I understood this quantity is being used throughout the paper.

We thank the reviewer for this point which we have now corrected. As we are working with the absolute value of this difference, the results remain the same.

45) Page 10: "... intracellular space having a higher volume than the extracellular." Isn't this only true when considering multiple neurons in the same region (including potentially soma as well)? In other words, given a 3D region, the summed intracellular space is higher than the extracellular one, but is it also true when considering an isolated small dendritic portion?

We thank the reviewer for this insightful comment. We have provided a detailed response to this very important issue under point #3. In short, the width of the extracellular space has a median value of 0.27 μm (Fig. S3I in (Tønnesen et al., 2018)), with the majority being between $0.1 - 0.3$ μm (Fig. 3C in (Tønnesen et al., 2018)). A recent review (Tønnesen et al., 2023) highlights that although the average width of the extracellular space could be even lower, around $40 - 80nm$, it can range from small gaps of $10 - 20nm$ to larger spaces in the range of $0.2 - 0.5$ μm. Even for these larger spaces, and considering that the dendritic diameter of the apical and basal dendrites of pyramidal neurons is in general > 0.5 μm (Gilman et al., 2017; Benavides-Piccione et al., 2020), the extracellular space is on average smaller than the intracellular one. This is now discussed in lines 468-471.

References

Abrahamyan, A., Eldstrom, J., Sahakyan, H., Karagulyan, N., Mkrtchyan, L., Karapetyan, T., Sargsyan, E., Kneussel, M., Nazaryan, K., Schwarz, J. R., Fedida, D., and Vardanyan, V. Mechanism of external K+ sensitivity of KCNQ1 channels. Journal of General Physiology, 155(5), 2023.

Aitken, P. G. and Somjen, G. G. The sources of extracellular potassium accumulation in the CA1 region of hippocampal slices. Brain Research, 369(1-2):163–167, 1986.

Amzica, F., Massimini, M., and Manfridi, A. Spatial buffering during slow and paroxysmal sleep oscillations in cortical networks of glial cells in vivo. Journal of neuroscience, 22(3):1042, 2002.

Benavides-Piccione, R., Regalado-Reyes, M., Fernaud-Espinosa, I., Kastanauskaite, A., Tapia-González, S., León-Espinosa, G., Rojo, C., Insausti, R., Segev, I., and Defelipe, J. Differential Structure of Hippocampal CA1 Pyramidal Neurons in the Human and Mouse. Cerebral Cortex, 30(2):730–752, 2020.

Benninger, C., Kadis, J., and Prince, D. A. Extracellular calcium and potassium changes in hippocampal slices. Brain Research, 187(1):165–182, 1980.

Brocard, F., Shevtsova, N. A., Bouhadfane, M., Tazerart, S., Heinemann, U., Rybak, I. A., and Vinay, L. Activity-dependent changes in extracellular Ca2+ and K+ reveal pacemakers in the spinal locomotor-related network. Neuron, 77(6):1047–1054, 2013.

Calì, C., Wawrzyniak, M., Becker, C., Maco, B., Cantoni, M., Jorstad, A., Nigro, B., Grillo, F., De Paola, V., Fua, P., and Knott, G. W. The effects of aging on neuropil structure in mouse somatosensory cortex—A 3D electron microscopy analysis of layer 1. PLoS ONE, 13(7):1–21, 2018.

Chen, T.-w., Wardill, T. J., Sun, Y., Pulver, S. R., Renninger, S. L., Baohan, A., Schreiter, E. R., Kerr, R. A., Orger, M. B., Jayaraman, V., Looger, L. L., Svoboda, K., and Kim, D. S. Nihms489004-1. 499 (7458):295–300, 2013.

Chung, W.-s., Allen, N. J., and Eroglu, C. Astrocyte & synapse. Cold Spring Harbor Laboratory Press, 7(a020370):1–19, 2015.

Connors, B., Dray, A., Fox, P., Hilmy, M., and Somjen, G. LSD's effect on neuron populations in visual cortex gauged by transient responses of extracellular potassium evoked by optical stimuli. Neuroscience letters, 13(2):147–150, 1979.

Connors, B. W., Ransom, B. R., Kunis, D. M., and Gutnick, M. J. Activity-dependent K+ accumulation in the developing rat optic nerve. Science, 216(4552):1341–1343, 1982.

Contreras, S. A., Schleimer, J. H., Gulledge, A. T., and Schreiber, S. Activity-mediated accumulation of potassium induces a switch in firing pattern and neuronal excitability type. PLoS Computational Biology, 17(5):1–25, 2021.

Dietz, A. G., Weikop, P., Hauglund, N., Andersen, M., Petersen, N. C., Rose, L., Hirase, H., and Nedergaard, M. Local extracellular K+ in cortex regulates norepinephrine levels, network state, and behavioral output. Proceedings of the National Academy of Sciences, 120(40):9, 2023.

Ding, F., O'donnell, J., Xu, Q., Kang, N., Goldman, N., and Nedergaard, M. Changes in the composition of brain interstitial ions control the sleep-wake cycle. Science, 352(6285):550–555, 2016.

Futamachi, K. J., Mutani, R., and Prince, D. A. Potassium activity in rabbit cortex. Brain research, 75 (1):5–25, 1974.

Genoud, C., Knott, G. W., Sakata, K., Lu, B., and Welker, E. Altered Synapse Formation in the Adult Somatosensory Cortex of Brain-Derived Neurotrophic Factor Heterozygote Mice. Journal of Neuroscience, 24(10):2394–2400, 2004.

Gilman, J. P., Medalla, M., and Luebke, J. I. Area-specific features of pyramidal neurons-a comparative study in mouse and rhesus monkey. Cerebral Cortex, 27(3):2078–2094, 2017.

Halassa, M. M., Fellin, T., Takano, H., Dong, J. H., and Haydon, P. G. Synaptic islands defined by the territory of a single astrocyte. Journal of Neuroscience, 27(24):6473–6477, 2007.

Heinemann, U., Lux, H. D., and Gutnick, M. J. Extracellular free calcium and potassium during paroxsmal activity in the cerebral cortex of the cat. Experimental brain research, 27(3-4):237–243, 1977.

Heinemann, U., Schaible, H. G., and Schmidt, R. F. Changes in extracellular potassium concentration in cat spinal cord in response to innocuous and noxious stimulation of legs with healthy and inflamed knee joints. Experimental brain research, 79(2):283–292, 1990.

Hibino, H., Inanobe, A., Furutani, K., Murakami, S., Findlay, I., and Kurachi, Y. Inwardly rectifying potassium channels: Their structure, function, and physiological roles. Physiological Reviews, 90(1): 291–366, 2010.

Hille, B., Hille, B., and Hille, B. Ionic channels of excitable membranes. Sinauer Associates, Sunderland, Mass, 2nd ed. edition, 1992.

Iacaruso, M. F., Gasler, I. T., and Hofer, S. B. Synaptic organization of visual space in primary visual cortex. Nature, 547(7664):449–452, 2017.

Jia, H., Rochefort, N. L., Chen, X., and Konnerth, A. Dendritic organization of sensory input to cortical neurons in vivo. Nature, 464(7293):1307–1312, 2010.

Kelly, J. P. and Van Essen, D. C. Cell structure and function in the visual cortex of the cat. The Journal of physiology, 238(3):515–547, 1974.

Knöpfel, T., Sweeney, Y., Radulescu, C. I., Zabouri, N., Doostdar, N., Clopath, C., and Barnes, S. J. Audio-visual experience strengthens multisensory assemblies in adult mouse visual cortex. Nature Communications, 10(1), 2019.

Larsen, A. P., Steffensen, A. B., Grunnet, M., and Olesen, S. P. Extracellular potassium inhibits Kv7.1 potassium channels by stabilizing an inactivated state. Biophysical Journal, 101(4):818–827, 2011.

Major, G., Larkum, M. E., and Schiller, J. Active properties of neocortical pyramidal neuron dendrites. Annual review of neuroscience, 36:1–24, 2013.

Park, J., Papoutsi, A., Ash, R. T., Marin, M. A., Poirazi, P., and Smirnakis, S. M. Contribution of apical and basal dendrites to orientation encoding in mouse V1 L2/3 pyramidal neurons. Nature communications, 10(1), 2019.

Petousakis, K. E., Park, J., Papoutsi, A., Smirnakis, S., and Poirazi, P. Modeling apical and basal tree contribution to orientation selectivity in a mouse primary visual cortex layer 2/3 pyramidal cell. eLife, 12:1–28, 2023.

Poolos, N. P., Mauk, M. D., and Kocsis, J. D. Activity-evoked increases in extracellular potassium modulate presynaptic excitability in the CA1 region of the hippocampus. Journal of neurophysiology, 58(2):404–416, 1987.

Rasmussen, R., Nicholas, E., Petersen, N. C., Dietz, A. G., Xu, Q., Sun, Q., and Nedergaard, M. Cortex-wide changes in extracellular potassium ions parallel brain state transitions in awake behaving mice. Cell reports, 28(5):1182–1194, 2019.

Scholl, B., Thomas, C. I., Ryan, M. A., Kamasawa, N., and Fitzpatrick, D. Cortical response selectivity derives from strength in numbers of synapses. Nature, 590(7844):111–114, 2021.

Shih, P. Y., Savtchenko, L. P., Kamasawa, N., Dembitskaya, Y., McHugh, T. J., Rusakov, D. A., Shigemoto, R., and Semyanov, A. Retrograde synaptic signaling mediated by K+ efflux through postsynaptic NMDA receptors. Cell reports, 5(4):941–951, 2013.

Singer, W. and Dieter Lux, H. Extracellular potassium gradients and visual receptive fields in the cat striate cortex. Brain research, 96(2):378–383, 1975.

Syková, E., Rothenberg, S., and Krekule, I. Changes of extracellular potassium concentration during spontaneous activity in the mesencephalic reticular formation of the rat. Brain research, 79(2):333–337, 1974.

Tønnesen, J., Inavalli, V. V., and Nägerl, U. V. Super-resolution imaging of the extracellular space in living brain tissue. Cell, 172(5):1108–1121, 2018.

Tønnesen, J., Hrabětová, S., and Soria, F. N. Local diffusion in the extracellular space of the brain. Neurobiology of Disease, 177(December 2022), 2023.

Tyurikova, O., Shih, P. Y., Dembitskaya, Y., Savtchenko, L. P., McHugh, T. J., Rusakov, D. A., and Semyanov, A. K+ efflux through postsynaptic NMDA receptors suppresses local astrocytic glutamate uptake. Glia, 70(5):961–974, 2022.

Vardalaki, D., Chung, K., and Harnett, M. T. Filopodia are a structural substrate for silent synapses in adult neocortex. Nature, 612(7939):323–327, 2022.

Wilson, D. E., Whitney, D. E., Scholl, B., and Fitzpatrick, D. Orientation selectivity and the functional clustering of synaptic inputs in primary visual cortex. Nature neuroscience, 19(8):1003–1009, 2016.
