# Supplementary material for: Local changes in potassium ions regulate input integration in active dendrites
Source: PLoS Biol. 2024 Dec 4;22(12):e3002935. doi: 10.1371/journal.pbio.3002935 (PMC11649091; doi:10.1371/journal.pbio.3002935)
Supplement: Attachment — Submitted filename: Response_to_reviewers_Rev2.docx [file pbio.3002935.s024.docx]

PLOS Biology Response to Reviewers

**Local changes in potassium ions regulates input integration in active dendrites**

Malthe S. Nordentoft, Naoya Takahashi, Mathias S. Heltberg, Mogens H. Jensen, Rune N. Rasmussen, and Athanasia Papoutsi

Correspondence:

*rune.rasmussen90@gmail.com*

[*papoutsi@imbb.forth.gr*](mailto:papoutsi@imbb.forth.gr)

Black text = Reviewers’ comments

Purple text = Authors’ responses

We would like to thank the reviewers for the feedback to our revised manuscript. We believe that the reviewer’s comments have increased the clarity and the impact of our work. Below is the response to each point raised.

Reviewer 1

In this paper the authors study the consequence of localized changes in extracellular potassium concentration on dendritic excitability and neuronal responses in abstract visual cortical neuron models. In their study they did not model explicitly the changes in the extracellular K+ concentration but assumed that it is elevated specifically in some dendritic segments. The effect of the elevated [K+] was then modeled as an increase in the reversal potential for the K+ ions for the duration of the simulations (~ 1s) that influences all K+ currents including voltage sensitive and NMDA currents.

I my previous review I raised a point regarding a fundamental assumption of the paper: that such large changes in [K+] can remain localised to a given dendritic segment. To support the claim of the authors I suggested to implement a simple simulation that combines modelling [K+] dynamics along an extended dendritic branch with realistic synaptic inputs. I argued that this simulation could reveal the potential spatial and temporal scale of the fluctuations in the [K+] concentration. I appreciate that the authors implemented such a simulation and reported the resulting changes in the K+ reversal potential. Even though I have some further comments regarding the details of these simulation, I found it already insightful: it demonstrated that within this model, changes in K+ reversal potential a localised within 30um and last for a few 100 ms. Although these simulations indicate spatio-temporally more dynamic changes in K+ reversal than that used throughout the paper, they are qualitatively similar, raising the possibility that the proposed mechanisms might actually contribute to structured fluctuations in dendritic excitability in vivo.

Before publishing the paper I would like to ask to consider a few minor changes:

- Consider showing [K+] and not just reversal potential in the simulations in S10-S14.

Following the reviewer’s comment, we have included Δ[K^+^]_o_ values over time (three additional bottom panels in S10 Fig.). Δ[K^+^]_o_ lies within the interval [1:5 mM], as per Fig. 1. As Δ[K^+^]_o_ closely follows ∆*E*_K_^+^ we decided to show these values only in S10 Fig. Please note that for S10-S13 Figs. and for the point dendrite model we have optimized the parameters (synaptic strength) for a longer window of synaptic activations (λ=80ms). This was done to better match the delayed peak changes seen in the frequency stimulation protocol (S15 Fig., following comment). As results are similar and conclusions are the same we did not modify the stimulation protocol in Fig. 4. All changes in the parameters and results are highlighted with purple in the S1, S2 text, methods and results sections respectively.

- I suggest to use rate-modulated Poisson spike train (inhomogenous Poisson process) according to a slowly changing stimulus orientation. I do not see the motivation behind the choice of the Poisson distribution for the spike delay used throughout the paper - it does not correspond to neither in vivo nor in vitro stimulation of these cells. Instead, I suggest to simulate a potential in vivo spike train by first generating a trajectory of stimulus orientation, then calculating the firing rate of each neuron as a function of orientation and finally sampling spikes from a Poisson process.

In the new S15 Fig. we have followed the reviewer’s recommendation for the stimulation of the synapses. Specifically, for a given stimulus orientation we calculated the input frequency of each synapse based on the presented stimulus and the synapse’s orientation preference (i.e. different number of spikes for synapses with preferred versus non-preferred inputs). Following we sampled spikes from a Poisson process using this input frequency as its mean. We have further increased the simulation time for this figure, to showcase the return to baseline after ~2sec of the stimulation. In that regard, stimuli presented sequentially with intervals > 2sec (trajectory of stimuli) would be similar to sequential simulations. We have thus omitted to perform a single simulation with a trajectory of stimuli that would be difficult to visualize. The new S15 Fig. shows a similar dynamics with the results shown in S10 Fig. Specifically, the peak ∆*E*_K_^+^ is reached within ~200ms, with the largest shift in *E*_K_^+^ is seen at the segment receiving similarly-tuned synapses. ∆*E*_K_^+^ is modulated by the stimulus orientation, mainly in the segment receiving similarly tuned inputs, whereas the diversely-tuned segments sustain a fixed ∆*E*_K_^+^ irrespective of the stimulus orientation, as per Fig. 1g. In addition, with the frequency modulation approach we observed an even higher compartmentalization of Δ[K^+^]_o_ potentially due to the spread of the input spikes and subsequent introduction of slower time scales.

Regarding the motivation for the 3 stimulation events and time intervals for synaptic activation, we envisage that our results show a generic mechanism of dendritic activity modulation throughout the cortex. In that regard, orientation preference was used as a framework of study but we would also like to keep our approach in the main text as generic as possible. For this reason we chose to use a stimulation protocol consisting of 3 events of activation, with the first stimulation event acting as the trigger of ∆*E*_K_^+^, and the two subsequent events acting as the experimental condition for a given *E*_K_^+^ shift. For each stimulation event, instead of activating all synapses synchronously, we varied their synaptic activation intervals. Following reviewer’s comment we have improved on our approach, by using a longer window of synaptic activations to better match the delayed pick changes seen in the frequency stimulation protocol. Still, we fully acknowledge that our approach lacks temporal resolution and we consider this work as a first step for characterizing the role of K^+^ hotspots in modulating dendritic integration. We further elaborate on this limitation of our work in the discussion section.

- Start the simulations from an approximate steady-state of the [K+]. Now the reversal potential increases even after distributed stimulation, which makes the interpretation of the results less clear. In the case of the Poisson process stimulus the steady state (more precisely, the long term average of the [K+]) will depend on the input rates and the parameters of the K/Na pump, but can be calculated analytically or numerically.

In our description of the simulation of the spatial and temporal properties of K^+^ diffusion (S2 text) we used the term “baseline” for [K^+^]_o_ to refer to the resting, steady-state [K^+^]_o_. As the reviewer correctly highlights, this [K^+^]_o_ is used as the starting point. We agree that the term “baseline” was ambiguous and we have thus included a description in S2 text, both in the first paragraph describing our general approach as well as when describing the Na^+^/K^+^ pump. Note that Δ[K^+^]_o_ is also anticipated in segments receiving diversly tuned inputs but lower from that of the similarly-tuned case, and not modulated by the stimulus orientation (Fig. 1g,h). In addition, in the S15 Fig. we show that, in the absence of any stimulus, [K^+^]_o_ slowly returns to the steady-state (Δ[K^+^]_o_ towards zero), indicating that the long term average of Δ[K^+^]_o_ will be zero in the absence of synaptic activity.

- S10: - Is this a single simulation or the average of many simulations? If I interpret the methods correctly, each segment is divided into nodes of area 0.01 um2. Do the curves here show the average across the nodes that belong to the same segment? Can you provide information about K-dynamics within the segments? Can you add the input spikes to the graph?

Presented data in S10-S13 Figs. are the indicative examples of a single simulation. We now clarify this in each figure, by stating that example traces are shown. Indeed, the curves correspond to the average ∆[K^+^]_o_ and ∆*E*_K_^+^ across all the nodes that belong to a segment. This is discussed in the last paragraph of S2 Text. Following reviewer’s suggestion, we have included raster plots to S10 and S15 Figs.

Regarding the K^+^-dynamics within the segments, we show in the response to reviewers the Δ[K^+^]_o_ dynamics in some randomly selected spatial points within the segment.

Simulation parameters correspond to the ones of S10 Fig. and data shown are for the segment receiving similarly tuned input and for 0° stimulus orientation. Note the overall similar dynamics of the different points in the relative small spatial scale. Given the large number of supplementary figures and as the spatial unit used throughout this study is 10μm, we decided to keep this figure in the response to the reviewers. Future work could further highlight the role of ∆[K^+^]_o_ in a more fine-scale spatial profile, potentially influencing single-synapse EPSPs.

- S12: increasing K_dec also affects the kinetic of the responses, but its effect (on the dynamics and the reversal potential) could depend on the steady state. This is another reason why I believe that these simulations should be started from the steady state.

As part of the third comment of the reviewer we have updated the S2 text to clarify that simulations start from resting, steady-state [K^+^]_o_. Please note that, as per Halnes et al., 2013, the inward flux *j*_K_^+^ due to the Na^+^/K^+^ pump only depends on the concentration difference away from steady state.

- S2 Text, Eq4; line 635, line 720: "The orientation tuning of the individual synapses was achieved by scaling the AMPA and NMDA conductance according to the synapse's tuning curve". Are these parameters really exchangeable? The strength of the synapse is a postsynaptic variable whereas its activation probability as a function of the stimulus (i.e., tuning curve) is presynaptic? The consequence of this hybrid solution is that it is not clear whether the simulations presented in the manuscript correspond to the in vivo situation (different number of spikes for synapses with preferred versus non-preferred inputs) or the in vitro situation (probably similar weights).

Following the second comment of the reviewer, we have implemented a presynaptic mechanism (input rate) for orientation tuning. Results are shown in S15 Fig. and a detailed response on this approach is under the second comment. In short, the main findings remain the same between the two approaches, with the main difference being the spatial spread of Δ[K^+^]_o_ that is more restricted in the frequency-modulated approach. The weight modulation was motivated by similar previous work (Park et al., 2019; Petousakis et al., 2023), where the resulting tuning curves were heavily validated on experimental data.

- This simple model simulates diffusion in essentially 1D (along the branch - the 2nd dimension is a special because of the periodic boundary conditions). Diffusion can have qualitatively different properties in 3D, and this could be discussed.

In our study we assumed a small extracellular environment. Consequently, the timescale needed for the system to be “well mixed” in this (radial) direction is magnitudes shorter than the time it takes to diffuse in the longitudinal directions. To simplify our model, we therefore chose to collapse the third dimension down to the two that span the surface of the dendrite. We have added a small discussion on the 3D diffusion in the S2 Text.

Finally, I far as I understand the model presented in Shih et al, 2013 (Fig 2.), the changes in [K+] remain localised to within 200 nm from the synaptic cleft. This spatial scale is even smaller than the scale assumed here and would not allow cooperativity between NMDA receptors targeting the same dendritic segment. Please discuss this discrepancy.

We thank the reviewer for this comment that gave us the chance to go back to the model of the Shih et al, 2013 paper. Indeed, although the temporal profile of ∆[K^+^]_o_ is of the same order (hundreds of milliseconds) the spatial scale is quite different, even though the value of the K^+^ diffusion coefficient (1um^2^/ms) is similar to ours (0.76um^2^/ms). Yet, as the publication is not accompanied by the model code, we are not confident that in the referenced model, the spatial diffusion of K^+^ ions was simulated in detail. Specifically, the authors state in the methods section that they set K^+^ concentration to [K^+^]_o_ (2.5 mM) in distances (radii) without receptor activation (i.e. they may have not accounted for spillover of K^+^ in the non-activated distances in the synaptic cleft). This is important as they implemented a “central” release of glutamate (activation of synapses initially occurs only within ~20nm at the center of the synapse) and glutamate is diffused from this release site to nearby radii (equation 5 in their methods section). This indicates that the spatial profile seen in Fig. 2 might be due to the diffusion of the glutamate and not due to the diffusion of the [K^+^]_o_. Please note that a wider glutamate release in the synaptic cleft (i.e. not only in the 20 nm center of the synapse) would also increase the reported spatial diffusion scale of Shih et al. As we are not confident regarding the actual implementation of Shih et al., and given that the equation and parameters for the K^+^ diffusion are similar in both works, we decided to omit this discussion from the manuscript as it would create confusion without increasing clarity.

Regarding the argument for ignoring [K+] changes caused by neighbouring segments: Vardalaki estimates that 25% of synapses are silent. Non-visually driven cells are also active, just their activity is not modulated by the visual stimulus. Cells tuned to a different orientation are not necessarily silent, they just fire with a lower rate (see for example Niell and Stryker, J Neurosci. 2008 for a quantitative spike metrics in the mouse visual cortex - I'm not aware of similar data from ferret). Furthermore, out of the 30-40 segments within 5um some could actually receive a clustered input tuned to a different orientation that would elevate the local K even if the target segment receives non-preferred input. Furthermore, the simulations with 1D diffusion (S10-14) showed that changes in [K+] can reach up to 30um. To me these considerations suggest that synapses on other segments can influence local [K], and if these changes are not correlated (e.g., state dependent changes during running versus still), then it will largely average out. This possibility should be mentioned in the discussion.

We thank the reviewer for highlighting this issue. Indeed, we also agree that synapses on other segments can influence the local [K^+^]_o_. We had thus included a text in the discussion section in our revised manuscript stating that synapses surrounding the dendritic segments may also participate or be affected by the local [K^+^]_o_ changes. This co-activation of synapses/clusters in nearby dendrites could either increase the noise levels or locally promote the synergy between synapses, dendrites, and neurons. If the reviewer has any other suggestion regarding this issue, we would be glad to include it.

Reviewer 2

The authors have made substantial improvements to the manuscript, and I am grateful for their thoughtful and detailed responses. I very much enjoyed reading the updated version. The complementary material added to the manuscript has strengthened the main results. I have three major comments remaining (and some minor comments that might help improve the clarity of the manuscript).

Major

1) Averaging of dendritic responses. In figs. 2, S1, and S2, the authors report averages over simulation trials, which may obscure a very interesting effect that is depicted in fig. 2g,h. In some trials, e.g., in fig. 2b, for 22.5 degrees difference, some of the grey lines show dendritic spikes while others don't. Because of that, the averages in fig. 2d might be showing intermediate values from when a spike is generated or not. Rather than plotting the average with SEM, I would like to suggest plotting the individual data points of each simulation alongside the line connecting the averages. Furthermore, SEM can also vanish for a very large number of simulations (or samples), and it would be more informative to show individual points, especially considering that the distributions may be multi-modal. It's difficult to interpret a mean with very low SEM (high confidence) if the mean does not reflect the behaviour that is most likely to occur but the intermediate behaviour between two opposite behaviours. Additionally, I think it's worth expanding the discussion about the effect of increased reliability of dendritic spikes caused by shifts in EK+, which is currently very short.

We agree with the reviewer’s concern for not showing the individual datapoints and we have now included in Fig. 2d. Following this addition it is now visible that ∆*E*_K_^+^ increases both the dendritic spike probability and dendritic spike duration. This effect is also visible by the individual traces shown S1 and S2 Figs. Following this comment, we have also modified our summary of results in the Introduction and Discussion sections to highlight this dual effect of ∆*E*_K_^+^ and we have added a respective sentence in the S1 and S2 figure captions.

Please note that, as part of our response to reviewer 1, for simulations in S10-S13 Figs. and for the point dendrite model we have optimized the parameters (synaptic strength) for a longer window for synaptic activations (λ=80ms) to account for the delayed pick changes seen in the frequency stimulation protocol (S15 Fig.). Figs. 2 and S1-5 are updated with the results with these parameters for direct comparison. As results are similar and conclusions are the same we did not modify the stimulation protocol in Fig. 4. All changes in the parameters and results are highlighted with purple in the S1, S2 text, methods and results sections respectively.

2) Equivalence between ΔEK+ changes and synaptic strength (and number of synapses). The new sentences in line 262 "This gain in the dendritic output was similar to the one expected from increasing the number of synapses (S5 Fig.)." and line 352 "This amplification was similar to the one expected by increasing the number of synapses (S8 Fig.)." reflect an important point, which is discussed in more details in the discussion section (lines 414-430). The first two sentences (lines 262 and 352), in my opinion, downplay the importance of the modulation of neuronal excitability through modulation of EK+. In other words, to get stronger weights, long-term potentiation has to occur just once (or until the high value is reached), resulting in long-lasting effects. The authors state that (line 414) "Compared to long-term plasticity mechanisms, [K+]o-dependent dendritic integration modulation requires minimum usage of resources.", but that's not the case if plasticity has to occur once to increase the weight to the optimal values. The temporal aspect of EK+ modulation is the key point here, as highlighted by the authors in the discussion section. I would like to suggest emphasising this point already in lines 262 and 352.

We thank the reviewer for highlighting this point. Indeed, the transient, selective boosting of activity in dendrites is the key feature of the ∆*E*_K_^+^ modulation. We have now included a short phrase in the respective results (sentences referenced by the reviewer) to already highlight this effect in these sections, before reaching the discussion.

3) Inhibition. I appreciate the inclusion of an example of the dendritic response when inhibition is present. However, a closer look at the parameter values indicates that inhibition is very weak compared to excitation. Thus, the conclusion that (line 227) "This effect persisted in the presence of non-specific inhibitory (GABAA) input (S2 Fig.)" is too strong. To support such a claim, the authors should test the limits of the model, by either increasing the strength of the inhibitory synapses, their firing-rate, and/or number. I would expect a very strong shunting effect from strong inhibitory inputs.

Following this comment we have updated S2 Fig. to show results with gradually increasing inhibitory conductance and inhibitory stimulation frequency, up to the point that dendritic spikes fail to be initiated in the point dendrite model. The ∆*E*_K_^+^-induced differences are diminished only in the latter case. We thank the reviewer for this suggestion as the information content of the data shown now in this figure has considerably improved.

Minor

4) Lines 175/176: "... increases as 1/R …" -> R is not defined. For R2=R1, ΔEK+ is undefined, as correctly pointed by the authors. However, I still find it useful to mention that it diverges as R2 approaches R1. This indicates that ΔEK+ varies from 0 to infinity.

We have modified the equation to omit the dummy variable R and we have added that ∆[K^+^]_o_ approaches infinity with 1/(R_1_-R_2_).

5) Line 177: "By multiplying … regime with the …" -> Shouldn't "with" be replaced by "by"?

Correction done.

6) Line 187: "... a technique which averages in space and time and likely …" -> I think there is a word missing right after "averages". What is this technique averaging?

The technique averages the measured [K^+^]_o_. We have added a clarification in the respective sentence.

7) Line 196: "... above the baseline" -> Isn't zero the baseline for ΔEK+? If not, I would like to suggest clarifying which baseline is being referred to here.

With the term “baseline” we were referring to the *E*_K_^+^ at rest. We have added a clarification in the respective sentence.

8) Line 225: "EK+ shifts caused a linear broadening of dendritic spikes (Fig. 2c, 2d, S1 Fig.);" Fig. 2d seems to portrait a nonlinear relationship between duration and ΔEK+ (maybe quadratic?). To claim a linear relationship, the authors should add the line to the plot. Otherwise, I would suggest deleting the word "linear" from the sentence.

We chose to delete the word “linear” from the respective line, as indeed the data could also be characterized by a non-linear relationship.

9) Line 253: The average synaptic activity (w) is not well defined for the point-dendrite neuron. For the statistical analysis (Fig. 1), it's clear that it normalised to the maximum response (the plot shows a.u., but isn't it normalised?). I would like to suggest avoiding arbitrary units here. Is it normalised by the maximum activity? In this case, what's the maximum activity? Referring to the methods would suffice. The methods may be improved with the inclusion of a few more details about it as well.

We have expanded in the methods section on the definition of the average synaptic activity (w) for the point-dendrite model, to indicate that it directly refers to Fig. 1d, e and that it was used in the point-dendrite to multiply the synaptic conductances (Methods section, “Point-dendrite model”). We have further included a reference in the respective results line (now line 250) to the methods section. Regarding Fig. 1, the activity of individual synapses, shown in Figs. 1c, d, is normalized to the maximum activity and we have updated the figure caption of Fig. 1c to explicitly state this. Consequently, the average synaptic activity, shown in Fig. 1e, will also be within the [0,1] range. Finally, the reviewer is correct regarding the “a.u.” and we have removed it from Figs. 1c, d e and Fig. 2e.

10) Line 259: "Using equation (3) we estimated the probability …" -> This sentence suggests that the probability of generating a dendritic spike comes directly from eq 3, but from the methods (line 681) I understood that the probability was estimated based on simulations. I would like to suggest clarifying this point.

For these data we used a combination of the statistical approach seen in Fig. 1, and the fit gained from eq. 3, to predict if a certain random sample of a similarly tuned segment would generate a spike, given the ∆*E*_K_^+^ and the stimulus orientation. Specifically, to estimate the probability of dendritic spike for a given presented orientation, we sampled the number of synapses and single-synapse orientation preferences as per Fig. 1b, resulting in the average synaptic activity per orientation, as in Fig. 1d, e, in 10^4^ segments. For each segment, we determined the needed ∆*E*_K_^+^ for each orientation to generate a dendritic spike based on eq. 3 (eq. 3 was derived from the biophysical, point-dendrite simulations). By comparing the needed ∆*E*_K_^+^ with a set ∆*E*_K_^+^ values (e.g. 0, 6, 10, 18mV) we identified if a dendritic spike would be generated and calculated dendritic spike probability over all the 10^4^ segments. The benefit of this approach is that it allows for the investigation of a lot of intermediate orientation values, in addition to high sampling. We have restructured and refined our method description in the respective methods section (“Linear dendritic spike function”) to increase clarity on this approach.

11) Line 275: ".. I-V curve using dVm/dt=I/C" -> The negative sign is missing, as it should be -I/C for the plots in fig 3 to reflect the correct stability of the fixed points.

Correction done.

12) Line 276: "down-stable" is not clearly defined. I interpreted it as the stable fixed-point for the membrane potential being in the down-state (hyperpolarized), opposite to the up-stable from the up-state. It may be worth defining the term "down-stable".

The reviewer is correct and we now clarify down-stable in the text.

13) Line 307: "... prolongs the duration of the up- and bistable states …" -> This sentence can be more precise. It prolongs the duration for which the system is described by up- and bistable states. The state of the dendrite is just one, whether it's constant, decreasing, or increasing in value depends on the input (dVm/dt=-I/C). The dendritic dynamics is described by dVm/dt which comprises fixed-point(s).

Correction done.

14) Line 310: Is "underlined" correct in this sentence?

As “can be predicted” also conveys our message, we removed the "underlined".

15) Line 314: Additional to "boost dendritic spike generation" the authors may add the change in probability of spike generation (see point 1).

We have added the phrase “can boost the reliability and duration of dendritic spikes” that better describes our results.

16) Line 463: Is "to or by" correct in this sentence?

Correction done.

17) Eq. 10: The sum symbol may not be necessary given that Iext and Iint incorporate all external and intrinsic currents.

We agree, given the definition of the Iext and Iint we provided in the text. We have also included the “A” in the phrase: “The latter is multiplied by the surface area, A, …”

18) Eq. 11: Vm should be subscript in the exp argument.

Correction done.

19) Eq. 11: The left-hand side should be Vm rather than dVm given that the update for this method is: Vm(t+dt) = V∞(t) + (Vm(t) - V∞(t))exp(-dt/τVM(t)). dVm indicates an infinitesimal difference (after - before), while eq 11 uses the solution of the differential equation when all but Vm are assumed to be constant between t and t+dt.

Thank you for this correction, we have now updated the eq. 11.

20) Eq. 12: What does the sum symbol indicate? The equation in the supplementary material describing synaptic currents has gsyn, B, A, and Mg(V), which seems to be missing in Eq. 12.

The symbol indicates the summation over the different types of synapses (AMPA, NMDA and GABA_A_). We have included this information in the description of equation 12. For consistency, we have further changed the gNMDA, gAMPA and gGABA_A_ to the bar versions, in S1 Text, equations 2,3.

21) Line 691: dVm/dt = -I/C -> minus sign also missing.

Correction done.

22) Line 722: Is lambda the average interval between events? To the best of my knowledge, the parameter for a Poisson distribution is the frequency of events, which is usually defined as lambda. This point is just a clarification for the authors to update the text if they find it useful.

We have implemented a time delay for synaptic activation following a stimulus event, where the delay is computed using a poisson distribution, with mean/spread lambda. For clarity, we always include the unit of lambda (ms), to show that we use it in the time domain. Following also the comment 2 of reviewer 1, we have performed a new simulations (S15 Fig.) that show that the frequency and timing produce similar results.

23) Fig. 1h: The y-axis labels of both plots are different. Aren't they supposed to represent the same variable?

We thank the reviewer for spotting this. Indeed, the 2 plots represent the same variable (both referring to the target orientation). We have updated the y-axis label by removing the “activity factor”.

24) Fig. 2b: The caption mentions a dotted line indicating the threshold to identify spikes. This line is not there or it's barely visible.

Thank you for spotting this, it was from a previous version of the figure. We have removed the respective line from the figure legend.

25) Fig. 2c: Would it be possible to include x- and y-axes to this plot with the dotted line at -30 mV to exemplify how the points in fig. 2d are calculated?

We have added the -30mV line.

26) Fig. 2f: The separation of ΔEK+ = 0 from the rest creates a box that looks like the colour map (which is on the right of the panel), especially given that the colours change like the colour map. Would it be possible to change it to avoid confusion to the reader?

We have moved the values for ∆*E*_K_^+^ = 0 closer to the rest of the plot. We would still like to keep it separate, as these values correspond to the control values (no *E*_K_^+^ change).

27) Fig. 3a-d: The values on the y-axis are missing.

We have added the values on the y-axis.

28) Fig. 3a-d: The arrowheads are bigger in the updated manuscript, but some of them are very distorted, e.g., the blue arrowhead between -20 and 0 mV in panel d. Would there be a way to have the arrowheads without distorting them? It's difficult to see them in many parts of the plot.

Arrows now fixed!

29) Supplementary Figs.: In some of the plots, the units are inside square brackets, but the main figures have parentheses. Parenthesis is the standard format according to SI.

We have updated the figures in SI, to include a consistent use of parenthesis instead of brackets to indicate units.

30) S1 Text: Temperature in Kelvin does not have the degree symbol.

Corrected in S1 text.

31) S1 Text: NMDA conductance is negative, but it has to be positive if conductance is defined as the inverse of the resistance. The sign problem can be solved by removing the negative sign from the Mgblock(Vm) function.

Thank you for this comment, as obviously the conductance cannot be negative. We have made the correction in S1 Text.
